# Efficacy of traditional Chinese medicine decoctions combined with conventional therapy for pediatric asthma: a network meta-analysis

**DOI:** 10.3389/fphar.2026.1780354

**Published:** 2026-06-25

**Authors:** Yan Zhao, Meina Li, Ai Ai, Xin He, Xingyu Liu, Weiwei Tian, Yingying Tan

**Affiliations:** 1 School of Basic Medical Sciences, Shaanxi University of Chinese Medicine, Xianyang, China; 2 School of Clinical Medicine, Shaanxi University of Chinese Medicine, Xianyang, China; 3 Pediatrics, The First Hospital of Yulin, Yulin, Shaanxi, China

**Keywords:** clinical efficacy, network meta-analysis, pediatric asthma, TCM decoction, TCM formulae

## Abstract

**Objective:**

This study aimed to explore the potential comparative effectiveness of various traditional Chinese medicine (TCM) decoctions plus conventional biomedical treatment (CBT) versus CBT alone in pediatric asthma using a Bayesian network meta-analysis (NMA).

**Methods:**

Seven databases were systematically searched for randomized controlled trials (RCTs) published up to 24 March 2025 that evaluated TCM decoctions in the treatment of pediatric asthma. The Bayesian NMA was conducted using R 4.4.1 and Stata 15.1. Risk of bias was assessed using the Cochrane tool (Review Manager 5.4.1), and interventions were tentatively ranked by surface under the cumulative ranking curve (SUCRA).

**Results:**

A total of 21 RCTs involving 2,068 patients and 9 distinct TCM decoctions were included. The risk of bias was high or unclear in all studies included, with particular concerns regarding allocation concealment and blinding. Compared with CBT alone, Qing Qi Hua Tan Tang (QQHTT) + CBT significantly improved forced expiratory volume in one second (FEV1) (mean difference [MD] 1.20; 95% credible interval [CrI] [1.10, 1.40]). Ma Xing Shi Gan Tang (MXSGT) + CBT significantly increased peak expiratory flow (PEF) (MD 12.00; 95% CrI [8.00, 17.00]). According to SUCRA, QQHTT + CBT (SUCRA = 99.9%) and MXSGT + CBT (SUCRA = 97.1%) ranked highest for FEV1 and PEF.

**Conclusion:**

Sensitivity analyses confirmed the stability of the results regarding FVC and IgG, with Ma Xing Shi Gan Tang He Su Ting Wan (MXSGTHSTW) + CBT and Xiao Qing Long Tang (XQLT) + CBT which was ranked first in all analyses. For FEV1, the top-ranked interventions (QQHTT, Da Qing Long Tang [DQLT], MXSGTHSTW) remained in the top three across all analyses, with minor changes in ranking attributable to differences in inclusion criteria across studies. For PEF, the rankings in sensitivity analysis 1 were influenced by a reduction in the number of eligible treatments. For IgE, no decoction showed statistically significant differences, and the rankings were sensitive to the conditions of the analysis. Although QQHTT and MXSGT demonstrated statistical advantages in this NMA, serious methodological limitations exist in the included studies, particularly the lack of blinding and allocation concealment. Moreover, the inability to fully ensure the comparability of TCM decoctions means that these findings should be regarded as preliminary, hypothesis-generating and exploratory.

**Systematic Review Registration:**

identifier CRD420251120217.

## 1 Introduction

Pediatric asthma is a heterogeneous illness characterized by chronic airway inflammation and airway hyperresponsiveness. Clinically, it manifests as recurrent wheezing, coughing, chest tightness, and difficulty in breathing. Globally, pediatric asthma is the most prevalent among children aged 6–7 years, followed by adolescents aged 12–16 years (Nandu Krishnan et al., 2022). The prevalence of asthma among Chinese children aged 3–7 years is 14.6% (Pediatric Professional Committee of the Chinese Medical Education Association and Asthma Collaborative Group of the Respiratory Group PBotCMAPRWCotR, 2025). According to incomplete statistics, asthma afflicts approximately 150 million children worldwide, and the prevalence is still on the rise (Wang and Liu, 2020). As stated in the Global Initiative for Asthma guidelines, current clinical treatment primarily involves anti-inflammatory, anti-asthmatic, and antispasmodic therapies, predominantly utilizing bronchodilators, corticosteroids, and theophylline-based medications (AsthmaGINA GIf, 2023). However, long-term use of these therapies may lead to oral fungal infections and recurrent respiratory infections. Consequently, it is imperative to find safe alternative therapies to reduce symptoms such as airway inflammation in pediatric asthma patients and enhance the efficacy of clinical treatment.

Traditional Chinese medicine (TCM), with a lower incidence of adverse reactions, is often regarded as a safe alternative. TCM has a long history of application for asthma treatment, particularly in compound prescriptions such as decoctions. To date, multiple meta-analyses have indicated that TCM decoctions can relieve inflammatory responses, reduce airway hyperresponsiveness, and improve clinical outcomes in children with asthma (Wu and Lyu, 2025; Wang et al., 2025; Zeng et al., 2022). These decoctions alleviate symptoms of asthma by reconciling internal organs, dispelling wind and dampness, and invigorating qi to consolidate superficies. Among the numerous decoctions, the widely used and representative classical prescriptions that demonstrate clear efficacy in managing the acute attack of pediatric asthma include Ma Xing Shi Gan Tang (MXSGT), Xiao Qing Long Tang (XQLT), She Gan Ma Huang Tang (SGMHT), and Qing Qi Hua Tan Tang (QQHTT) (Cai et al., 2020; Li et al., 2017; Wang L. et al., 2020; Wang S. N. et al., 2021). A traditional meta-analysis published in 2021 demonstrated the efficacy of XQLT for alleviating the clinical symptoms of cough variant asthma and improving lung function in children. However, it did not directly compare different TCM decoctions and included a limited number of studies with high heterogeneity (Wang Y. L. et al., 2021). Another meta-analysis in 2022 distinguished between cold-type and heat-type asthma by differences in the pattern of syndrome. However, it solely focuses on ranking interventions by overall response rate and fails to discuss the advantages of the prescriptions in detail based on specific pathological indicators (e.g., IgE, FVC) (Zhang et al., 2022).

Consequently, the specific differences in therapeutic effects among various TCM decoctions in patients with pediatric asthma remain unclear, and the optimal treatment regimen remains remain defined. Bayesian network meta-analysis (NMA) enables a comprehensive evaluation of the relative efficacy of multiple treatment regimens by integrating data from direct and indirect comparisons and calculating the surface under the cumulative ranking curve (SUCRA). While conventional meta-analyses have consistently demonstrated that TCM combined with conventional biomedical treatment (CBT) significantly optimizes pulmonary function indicators (e.g., FEV1, FVC, PEF) (Du et al., 2025; Ling et al., 2025) and immune markers (e.g., IgE, IgG) (Han and Zhu, 2009; Ji et al., 2025), our NMA sought to further clarify the comparative advantages of specific prescriptions and support the synergistic role of these interventions in asthma management. Thus, this NMA evaluated the efficacy of different TCM decoctions in treating pediatric asthma and compared them with CBT. The findings are expected to fill the gaps in existing research and inform clinical decision-making for treating pediatric asthma with TCM.

## 2 Materials and methods

This meta-analysis was reported in accordance with the Preferred Reporting Items for Systematic Reviews and Meta-Analyses (PRISMA) guidelines (Hutton et al., 2015) and was registered in the International Prospective Register of Systematic Reviews (PROSPERO; registration number: CRD420251120217).

### 2.1 Specification for characterization and reporting of traditional Chinese medicine preparations

This study is a secondary analysis (NMA) of published randomized controlled trials (RCTs). Therefore, we were unable to obtain original data on the batches of the botanical raw material, including morphological identification, chemical fingerprint profiles, quantitative data on marker metabolites, and detailed parameters regarding the decoction process. All information regarding the composition of the decoctions (including specific botanical drugs and dosages) was extracted solely from the original articles.

Undeniably, the lack of original data posed a significant limitation, as it fundamentally prevented us from verifying whether the actual chemical compositions of the TCM decoctions across different studies were comparable. Although we assumed that decoctions with the same name share similar core ingredients, we recognized that unrecorded differences in botanical sources, processing methods, decocting procedures, and decoction modifications based on syndrome differentiation may introduce significant heterogeneity and potentially violate the transitivity assumption of NMA. This limitation has been explicitly addressed in the Discussion section, and a complete list of Consensus-based Phytochemical Characterisation of Medicinal Plant preparations (ConPhyMP) entries is provided (Supplementary Tables S1, S2).

#### 2.1.1 Preparation and verification of decoctions

All included decoctions were prepared using the traditional decoction method and administered orally, at one dose per day, divided into two or three separate intakes. According to the *Pharmacopoeia of the People’s Republic of China* (2025 edition), the 14th Five-Year Plan textbook *Formulas of Chinese Medicine*, *Youyou Jicheng*, and *Yifang Kao*, the standard compositions, core marker metabolites, and comparisons with those reported in the included studies are presented in Supplementary Table S3. Among the included original studies, 57% (12/21) had compositions fully consistent with the standard decoctions, and 100% (21/21) reported the dosage ranges for crude medicinal materials.

This study strictly adhered to the International Code of Botanical Nomenclature and performed taxonomic verification for all traditional Chinese medicinal materials used in the included studies, as shown in Supplementary Table S4. The Latin binomials of all medicinal materials and their corresponding plant species were verified against the *Pharmacopoeia of the People’s Republic of China* (2025 edition) and the Medicinal Plant Names Services (MPNS) database of the Royal Botanic Gardens, Kew (https://mpns.kew.org/mpns-portal/). The medicinal parts used complied with the requirements of the *Pharmacopoeia of the People’s Republic of China* (2025 edition).

#### 2.1.2 Compliance with laws and regulations

All prescriptions included in this study are classical and famous decoctions recorded in the 2025 edition of the *Pharmacopoeia of the People’s Republic of China*. These decoctions comply with the Traditional Chinese Medicine Law of the People’s Republic of China as well as relevant plant quarantine regulations, and do not entail the collection of cross-border species.

#### 2.1.3 Assessment of comparability

According to the theory of TCM decoctions, the monarch and minister botanical drugs directly target the core pathogenesis, whereas the addition or subtraction of assistant or guiding botanical drugs represents routine clinical adjustments (Wang Z. B. et al., 2024). These adjustments aimed at tailoring treatment to the individual without altering the fundamental therapeutic direction of the decoction.

This principle has significant implications for NMAs. If studies reporting the same decoction name actually used formulations with different monarch or minister botanical drugs, incorporating them under the same treatment node may obscure true differences in pharmacological effects and violate the transitivity assumption. To address this issue, we established the following comparability criteria: two decoctions are considered comparable only when their monarch and minister botanical drugs are completely consistent with the standard decoction specified in the *Pharmacopoeia of the People’s Republic of China* (2025 edition). If the monarch and minister botanical drugs were substituted, omitted, or fundamentally altered, the study was considered to represent a different therapeutic strategy and was excluded from the main analysis. A comparison of the complete composition of decoctions in each included study with the corresponding standard decoctions is presented in Supplementary Table S3.

After applying the criteria to the 24 eligible RCTs, we identified 3 studies with changes in core ingredients. One study on Liu Jun Zi Tang (LJZT) replaced the monarch botanical drug, ginseng, with *radix pseudostellariae*. One study on Ren Shen Wu Wei Zi Tang (RSWWZT) omitted the minister botanical drug *Atractylodes macrocephala* and added medicated leaven, barley malt, and *Lycium barbarum*. One study on XQLT omitted the minister botanical drug *Cinnamon twig* and added *Platycodonis Radix*. These three RCTs were excluded from the main analysis. Finally, 21 studies were included.

Although we defined comparability based on the consistency of the monarch and minister botanical drugs in the decoction according to the theory of TCM decoctions, we acknowledge that this is only an imperfect surrogate measure. A significant amount of heterogeneity remains unexplained, including differences in the quality of TCM botanical drugs, batch-to-batch variability, processing technology, and decocting procedures (e.g., extraction time, temperature, and solid-to-liquid ratio). These unmeasured factors may fundamentally violate the transitivity assumption of NMA. Therefore, this critical limitation must be fully considered when interpreting the results of this study.

### 2.2 Inclusion criteria

Inclusion criteria were as follows: (i) Population: children meeting the diagnostic criteria for pediatric asthma; (ii) Interventions: Any TCM decoction administered in combination with CBT, with or without decoction modifications that preserve the monarch and minister botanical drugs as defined by standard prescriptions; (iii) Comparison: CBT; (iv) Outcomes: pulmonary function (FEV1, PEF, and FVC) and asthma-related immune markers (immunoglobulin E (IgE) and immunoglobulin G (IgG)). Primary outcomes were FEV1 and PEF, while secondary outcomes comprised FVC, IgE, and IgG. All studies were selected based on asthma-related outcomes, rather than infection parameters. (v) Study design: RCTs.

### 2.3 Exclusion criteria

Exclusion criteria were as follows: (i) studies on pediatric asthma with concomitant major medical conditions (e.g., cardiac, cerebral, hepatic, or renal impairment); (ii) studies in which the interventions involved other types of Chinese commercial polyherbal preparations (CCPP) or decoctions combined with other therapies (e.g., acupuncture or massage); (iii) studies lacking a control group or utilizing only self-control designs; and (iv) studies with ineligible designs or those with variables that could not be converted to binary variables, such as cohort studies, animal studies, or clinical experience reports.

### 2.4 Literature search

Seven databases were searched, including Weipu Chinese Scientific and Technical Journal Database (VIP), Wanfang Data Knowledge Service Platform (Wanfang Data), China National Knowledge Infrastructure (CNKI), PubMed, Embase, Cochrane Library, and Web of Science. The search strategy was developed using combinations of subject headings and free-text terms. The search terms applied in the Chinese databases included: child, pediatric, asthma, wheezing, and shortness of breath. The search terms applied in the English databases included: child, children, pediatrics, asthma, bronchial, decoction, tang, and fang. The search covered publications from the inception of the databases to 24 March 2025. The search strategies for all databases are detailed in Supplementary Table S5.

### 2.5 Literature screening and data extraction

Based on the predefined eligibility criteria, two researchers (Yan Zhao and Meina Li) independently conducted the literature screening. First, all retrieved records were imported into EndNote 20 software to remove duplicates. Next, ineligible articles were excluded based on title and abstract screening. Finally, the remaining articles were assessed for eligibility via a full-text review. Any disagreements between the two researchers were resolved through consultation with a third researcher (Xin He). Data were extracted from the included studies independently by two researchers (Yan Zhao and Meina Li) using a pre-designed data extraction form. Any discrepancies were resolved through discussion with a third researcher (Xin He). Basic information extracted included the article title, authors, publication year, source, origin of cases, country or region, multi-center or single-center design, total number of cases, number of participants in the experimental and control groups, age, disease duration, interventions, controls, and outcome measures. The included studies were also categorized based on the codes of the intervention protocols involved.

### 2.6 Quality assessment

The Cochrane risk of bias tool was applied to evaluate the methodological quality of each included study. Two researchers (Yan Zhao and Meina Li) independently assessed the studies across seven domains: random sequence generation, allocation concealment, blinding of participants and personnel, blinding of outcome assessors, incomplete outcome data, selective reporting, and other biases. The studies were categorized as having an unclear risk, low risk, or high risk of bias. The assessment results were cross-checked, and any discrepancies were resolved through discussion or consultation with a third researcher (Xin He). Figures were generated using RevMan 5.4.

### 2.7 Statistical methods

**FIGURE 1:**
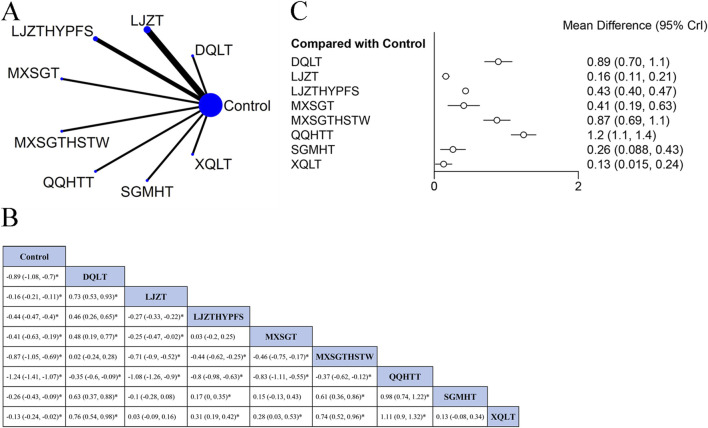
Network diagram and meta-analysis results. **(A)** Network diagram for FEV1; Note: The dots represent different interventions, and the dot size indicates the sample size for the intervention. The line thickness reflects the number of studies directly comparing the two interventions connected by the line. The results showed that no closed loops existed for any outcome measures. Thus, local inconsistency testing was not feasible. **(B)** Relative effects of various TCM decoctions on FEV1. Note: All data are given as mean difference and 95% credible interval. Data on comparisons between treatment groups should be read from left to right. The estimated effect for a TCM decoction is shown in the intersection of the column and the row representing the decoctions. Significant results are marked with*. **(C)** The forest plot visualizes the mean difference and 95% credible interval (CrI) for each intervention relative to the Control group. The central vertical line represents the zero effect line. The results indicate that the majority of the point estimates (e.g., DQLT, LJZT, MXSGTHSTW, QQHTT) are located to the right of the zero line, suggesting that these interventions are generally associated with positive improvements (e.g., in FEV1) compared to the Control. Specific numerical estimates and corresponding 95% CrIs are detailed on the right side of the plot.

**FIGURE 2:**
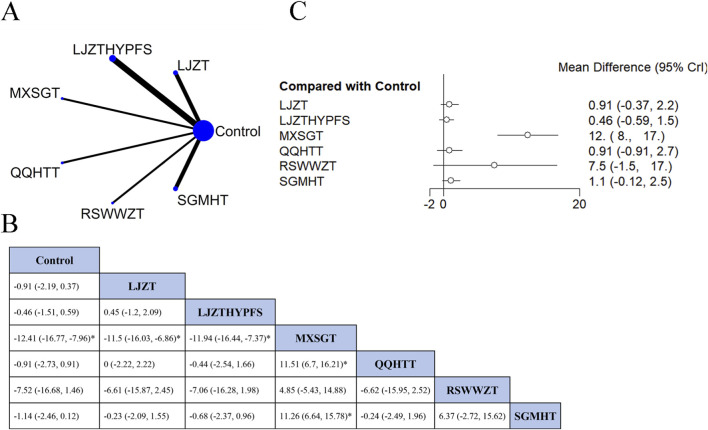
Network diagram and meta-analysis results. **(A)** Network diagram for PEF. Note: The dots represent different interventions, and the dot size indicates the sample size for the intervention. The line thickness reflects the number of studies directly comparing the two interventions connected by the line. The results showed that no closed loops existed for any outcome measures. Thus, local inconsistency testing was not feasible. **(B)** Relative effects of different TCM decoctions on PEF. Note: All data are given as mean difference and 95% credible interval. Data on comparisons between treatment groups should be read from left to right. The estimated effect for a TCM decoction is shown in the intersection of the column and the row representing the decoctions. Significant results are marked with*. **(C)** The forest plot displays the mean difference and 95% credible interval (CrI) for each intervention compared with the Control group. The central vertical line indicates the zero effect line. Notably, while most comparisons such as LJZT and QQHTT show modest effects, the interventions MXSGT and RSWWZT exhibit exceptionally large point estimates and extremely wide 95% CrIs (e.g., 12. [8., 17.] and 7.5 [-1.5, 17.]), indicating high uncertainty or wide variability in effect sizes. Specific numerical values are listed on the right.

**FIGURE 3:**
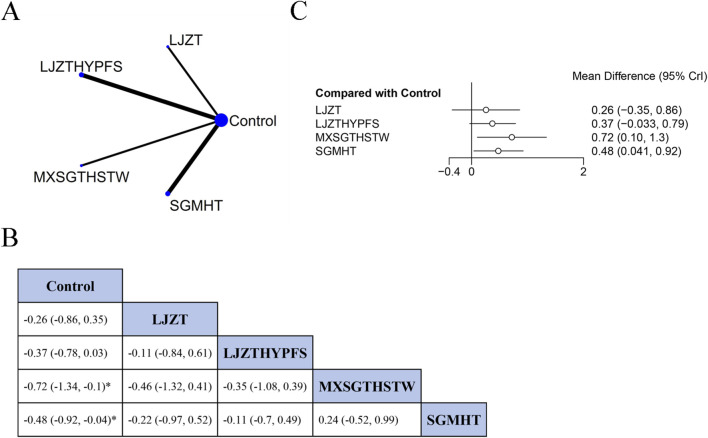
Network diagrams and meta-analysis results. **(A)** Network diagram for FVC. Note: The dots represent different interventions, and the dot size indicates the sample size for the intervention. The line thickness reflects the number of studies directly comparing the two interventions connected by the line. The results showed that no closed loops existed for any outcome measures. Thus, local inconsistency testing was not feasible. **(B)** Relative effects of different TCM decoctions on FVC. Note: All data are given as mean difference and 95% credible interval. Data on comparisons between treatment groups should be read from left to right. The estimated effect for a TCM decoction is shown in the intersection of the column and the row representing the decoctions. Significant results are marked with*. **(C)** The forest plot illustrates the mean difference and 95% credible interval (CrI) for the interventions compared with the Control group. The vertical line at zero signifies no difference. The results show that MXSGTHSTW and SGMHT are significantly more effective than Control, as their 95% CrIs do not cross the zero line. Conversely, the 95% CrIs for LJZT and LJZTHYPFS cross the zero line, indicating no statistically significant difference compared to Control. Specific numerical values are detailed on the right.

**FIGURE 4:**
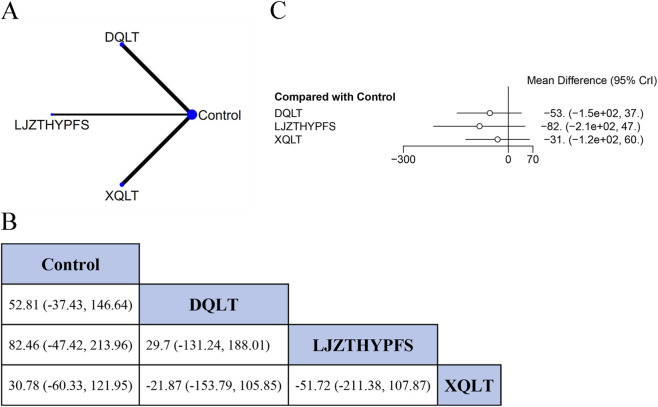
Network diagram and meta-analysis results. **(A)** Network diagram for IgE. Note: The dots represent different interventions, and the dot size indicates the sample size for the intervention. The line thickness reflects the number of studies directly comparing the two interventions connected by the line. The results showed that no closed loops existed for any outcome measures. Thus, local inconsistency testing was not feasible. **(B)** Relative effects of different TCM decoctions on IgE. Note: All data are given as mean difference and 95% credible interval. Data on comparisons between treatment groups should be read from left to right. The estimated effect for a TCM decoction is shown in the intersection of the column and the row representing the decoctions. Significant results are marked with*. **(C)** The forest plot displays the mean difference and 95% credible interval (CrI) for each intervention relative to the Control group. The vertical line at zero indicates the null effect. In contrast to most other figures, here, all point estimates (DQLT, LJZTHYPFS, XQLT) are located to the left of the zero line, with negative values (e.g., -53, -82, -31), suggesting that these interventions are associated with a numerical decrease in the measured outcome compared to Control. The specific numerical values and their 95% CrIs are listed on the right.

**FIGURE 5:**
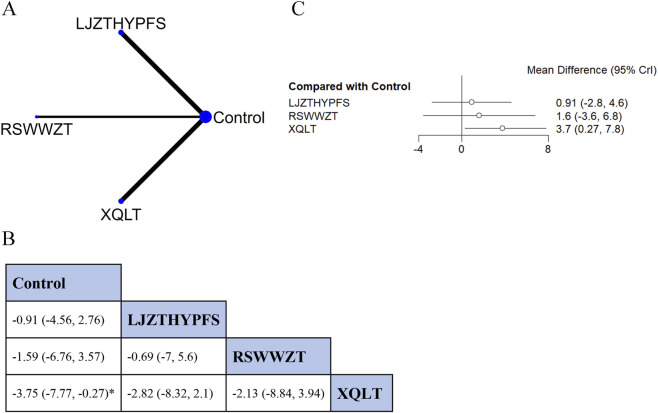
Network diagram and meta-analysis results. **(A)** Network diagram for IgG. Note: The dots represent different interventions, and the dot size indicates the sample size for the intervention. The line thickness reflects the number of studies directly comparing the two interventions connected by the line. The results showed that no closed loops existed for any outcome measures. Thus, local inconsistency testing was not feasible. **(B)** Relative effects of different TCM decoctions on IgG. Note: All data are given as mean difference and 95% credible interval. Data on comparisons between treatment groups should be read from left to right. The estimated effect for a TCM decoction is shown in the intersection of the column and the row representing the decoctions. Significant results are marked with*. **(C)** The forest plot visualizes the mean difference and 95% credible interval (CrI) for the interventions compared with the Control group. The vertical line represents the zero effect line. The results show that the majority of the 95% CrIs (for LJZTHYPFS and RSWWZT) cross the zero line, indicating no statistically significant difference. However, the intervention XQLT shows a significant difference, as its 95% CrI is located entirely to the left of the zero line with negative values. Specific numerical estimates are detailed on the right.

In this study, a Bayesian NMA was conducted using JAGS 4.3.1 in an R 4.4.1 environment. Given the potential clinical heterogeneity among trials regarding disease stage, specific decoctions of botanical drugs, treatment duration, and concomitant medications, a random-effects model was *a priori* selected for all outcomes, even when the statistical heterogeneity (I^2^) was low. The analysis focused primarily on continuous outcomes, with effect sizes expressed as mean differences (MDs). For dichotomous outcomes, hazard ratios (HRs) were pooled. Compliance with the transitivity assumption was qualitatively assessed by comparing potential effect modifiers (age, asthma stage, disease severity, TCM syndrome differentiation, conventional biomedical treatment regimens, and duration of intervention) across groups; the distribution of these modifiers in the included studies is detailed in Supplementary Table S6. No systematic violations were found. Since the evidence networks for all outcomes exhibited a star-shaped structure centered on conventional treatment with no closed loops (Figures 1A, 2A, 3A, 4A, 5A), it was impossible to test for local inconsistency using the node-splitting method. Therefore, all main analyses were conducted using the consistency model. Moreover, global consistency was assessed using the difference in the Deviance Information Criterion (DIC) between the consistency and inconsistency models. For all outcomes that could be fitted to the inconsistency model, the DIC difference was less than 5, supporting the consistency assumption.

NMA diagnostics and R code are provided in Supplementary Material 2. For continuous variables, MD served as the effect size. In cases of inconsistent units, the standardized mean difference (SMD) was used. All effect sizes were presented with 95% credible intervals (CrIs). For all continuous outcomes, the MD was calculated as: MD = (TCM + CBT) – CBT. For FEV1, FVC, PEF, and IgG, higher values indicate better improvement. A positive MD indicated a benefit of the TCM combined therapy. For IgE, lower values indicated better improvement. A negative MD indicated a benefit of the TCM combined therapy. A 95% CrI not crossing 0 indicated statistical significance. SUCRA values were employed to assess the relative ranking of different TCM decoctions across study indicators. A higher SUCRA value indicated a greater probability that the intervention was the most effective (Wang Z. B. et al., 2024). This allowed for a probability ranking of efficacy indicators among interventions to identify the optimal intervention. To evaluate potential publication bias in the included studies, comparison-adjusted funnel plots were constructed. The consistency and inconsistency models were compared utilizing the DIC. Since the DIC difference was <5, suggesting good consistency, the consistency model was applied.

In clinical practice, TCM decoctions are typically used for individualized treatment by adding or subtracting assistant and guiding botanical drugs based on the patient’s constitution, while retaining the core monarch and minister botanical drugs of classical decoctions. To reflect the actual TCM treatment patterns, the main analysis of this study included all eligible studies, in which the decoctions used encompassed both strictly standardized decoctions and modified versions that retained the core decoction composition. Within this analytical framework, each therapeutic decoction in the NMA does not represent a fixed chemical formulation, but rather a clinical treatment strategy, that is, the therapeutic principles embodied by the decoction and its rationally modified versions that have been clinically validated.

To assess the robustness of the main analysis results, we conducted two sensitivity analyses. Sensitivity analysis 1 included only RCTs using strictly standardized decoctions (without any addition, subtraction, or substitution of botanical drugs), aiming to assess whether decoction modifications affect effect estimates and rankings. Sensitivity analysis 2 re-included three RCTs (Wang L. et al., 2020; Ji et al., 2025; Zhao et al., 2020) that were excluded from the main analysis due to substitutions of monarch or minister botanical drugs, aiming to quantify the impact of the exclusion on the main analysis results. For each sensitivity analysis, all NMA models were rerun using the same statistical methods as in the main analysis. Model convergence was assessed using the Gelman-Rubin diagnostic statistic (R-hat), with R-hat <1.05 considered sufficient convergence. The DIC values across analyses were compared to evaluate model fit.

## 3 Results

### 3.1 Results of literature screening

**FIGURE 6:**
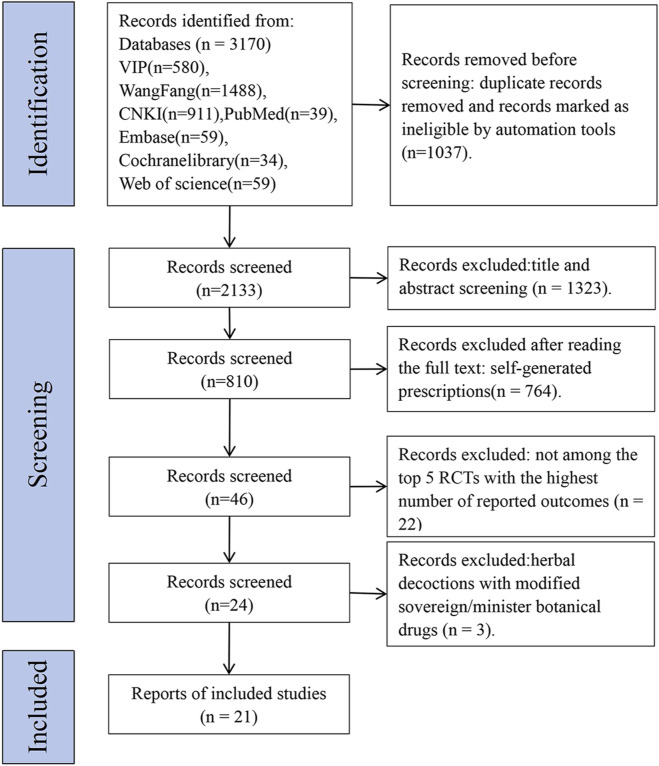
Literature screening process.

The databases were searched for published studies on TCM decoctions for treating asthma in children. The detailed study selection procedure is depicted in the PRISMA flowchart in Figure 6. Initially, 3,170 articles were identified. After removing 1,037 duplicates, the titles, abstracts, and full texts of the remaining articles were further evaluated based on the eligibility criteria. After eliminating studies on self-formulated prescriptions and RCTs with changes in monarch or minister botanical drugs, 21 studies were ultimately included (Figure 6).

### 3.2 Basic characteristics of included studies

All 21 included studies (Zhao et al., 2020; Gao and Huo, 2020; Zhang, 2022; Jin, 2021; Xiao, 2017; Liu et al., 2019; Li and Yang, 2010; Liu and Lu, 2021; Fang, 2022; Liu et al., 2021; Wang, 2014; Song et al., 2016; Zhou et al., 2021; Li, 2021; Liang, 2022; Zhang, 2024; Ding, 2022; Liu et al., 2023; Lu et al., 2017; Dong, 2021; Yuan, 2021) were double-arm trials, involving a total of 2,068 patients, with 1,034 in the experimental groups and 1,034 in the control groups. Each trial enrolled between 46 and 200 patients, with a mean age ranging from 1.8 to 11.2 years. A total of 9 TCM decoctions were evaluated, including Da Qing Long Tang (DQLT) (3 studies), Liu Jun Zi Tang He Yu Ping Feng San (LJZTHYPFS) (or Yu Ping Feng San He Liu Jun Zi Tang, YPFSHLJZT) (6 studies), Liu Jun Zi Tang (LJZT) (2 studies), Ma Xing Shi Gan Tang (MXSGT) (1 study), Ma Xing Shi Gan Tang He Su Ting Wan (MXSGTHSTW) (1 study), RSWWZT (2 studies), She Gan Ma Huang Tang (SGMHT) (2 studies), Xiao Qing Long Tang (3 studies), and Qing Qi Hua Tan Tang (QQHTT) (1 study). The basic characteristics of the included studies are presented in Supplementary Table S6.

### 3.3 Quality assessment of included studies

**FIGURE 7:**
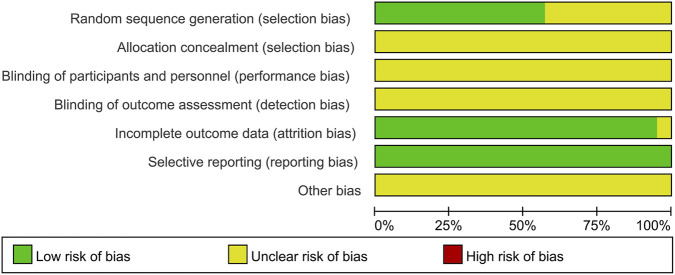
Items of risk of bias assessment for each incorporated study on TCM decoctions used to treat pediatric asthma.

**FIGURE 8:**
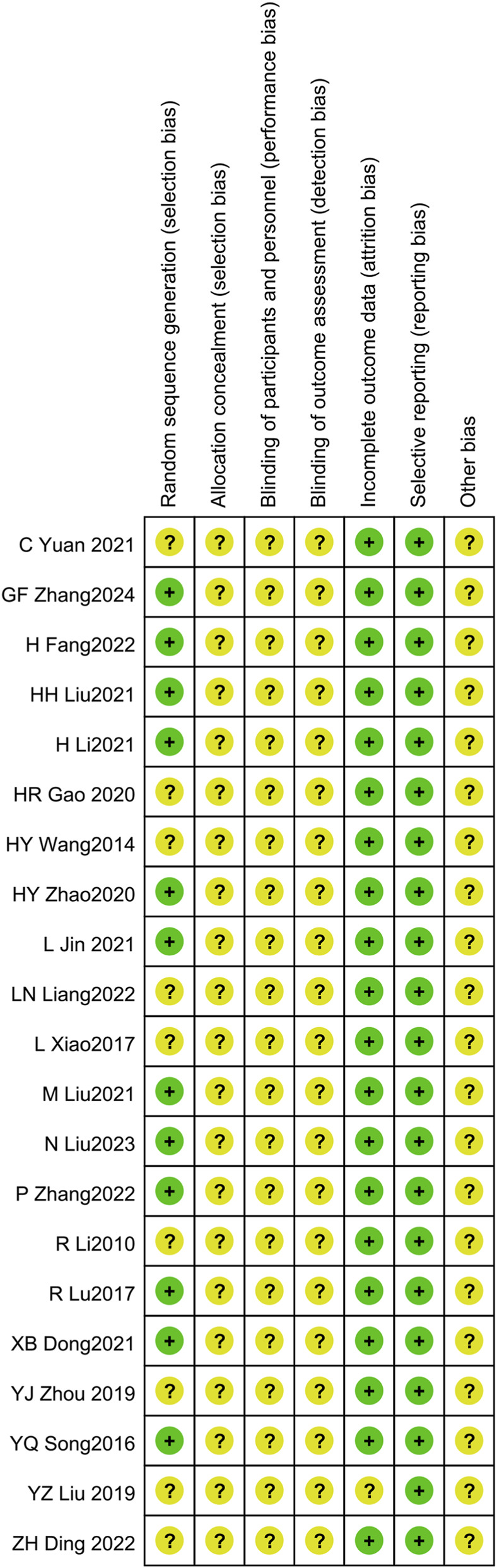
Summary of risk of bias assessment.

Among the 21 included RCTs (Zhao et al., 2020; Gao and Huo, 2020; Zhang, 2022; Jin, 2021; Xiao, 2017; Liu et al., 2019; Li and Yang, 2010; Liu and Lu, 2021; Fang, 2022; Liu et al., 2021; Wang, 2014; Song et al., 2016; Zhou et al., 2021; Li, 2021; Liang, 2022; Zhang, 2024; Ding, 2022; Liu et al., 2023; Lu et al., 2017; Dong, 2021; Yuan, 2021), 13 (Zhao et al., 2020; Zhang, 2022; Jin, 2021; Liu and Lu, 2021; Fang, 2022; Liu et al., 2021; Song et al., 2016; Zhou et al., 2021; Li, 2021; Zhang, 2024; Liu et al., 2023; Lu et al., 2017; Dong, 2021) described a valid random sequence generation method and were classified as having a low risk of bias for random sequence generation. The remaining 8 studies (Gao and Huo, 2020; Xiao, 2017; Liu et al., 2019; Li and Yang, 2010; Wang, 2014; Liang, 2022; Ding, 2022; Yuan, 2021) merely mentioned “randomization” without further details and were classified as having an unclear risk of bias for random sequence generation. Regarding incomplete outcome data, one study (Gao and Huo, 2020) reported loss to follow-up but did not discuss its potential impact, and was therefore classified as having an unclear risk. All other studies were classified as having a low risk of bias for incomplete outcome data. In terms of selective reporting, all studies reported results consistent with their analysis protocols and were thus classified as having a low risk of bias. No study explicitly described allocation concealment methods; therefore, all were judged as having an unclear risk of bias in this domain. None of the studies mentioned the blinding of outcome assessors; therefore, all studies were classified as having an unclear risk of bias for this domain. None of the studies mentioned other factors that could systematically affect the validity of the study results; therefore, all studies were classified as having an unclear risk of bias for other biases. The risk of bias assessment for the included studies is illustrated in Figures 7, 8.

### 3.4 NMA results of TCM decoctions for treating pediatric asthma

#### 3.4.1 FEV1

FEV1 was reported in 11 RCTs involving 8 TCM decoctions (Figure 1A). According to the NMA results depicted in Figures 1B,C, DQLT + CBT (MD 0.89; 95% CrI [0.70, 1.10]), Liu Jun Zi Tang He Yu Ping Feng San (LJZTHYPFS) + CBT (MD 0.43; 95% CrI [0.40, 0.47]), LJZT + CBT (MD 0.16; 95% CrI [0.11, 0.21]), MXSGT + CBT (MD 0.41; 95% CrI [0.19, 0.63]), Ma Xing Shi Gan Tang He Su Ting Wan (MXSGTHSTW) + CBT (MD 0.87; 95% CrI [0.69, 1.10]), SGMHT + CBT (MD 0.26; 95% CrI [0.088, 0.43]), XQLT + CBT (MD 0.13; 95% CrI [0.015, 0.24]), and QQHTT + CBT (MD 1.20; 95% CrI [1.10, 1.40]) were more effective in increasing FEV1 than CBT alone.

#### 3.4.2 PEF

PEF was reported in 10 RCTs involving 6 TCM decoctions (Figure 2A). According to the NMA results depicted in Figures 2B,C, MXSGT + CBT (MD 12.00; 95% CrI [8.00, 17.00]) was more effective in increasing PEF than CBT alone.

#### 3.4.3 FVC

FVC was reported in 6 RCTs involving 4 TCM decoctions (Figure 3A). According to the NMA results depicted in Figures 3B,C, MXSGTHSTW + CBT (MD 0.72; 95% CrI [0.10, 1.30]) and SGMHT + CBT (MD 0.48; 95% CrI [0.04, 0.92]) demonstrated greater efficacy in increasing FVC than CBT alone.

#### 3.4.4 IgE

IgE was reported in 5 RCTs involving 3 TCM decoctions (Figure 4A). According to the NMA results depicted in Figures 4B,C, TCM + CBT showed no statistically significant difference in reducing IgE levels compared to CBT alone.

#### 3.4.5 IgG

IgG was reported in 6 RCTs involving 3 TCM decoctions (Figure 5A). According to the NMA results depicted in Figures 5B,C, XQLT + CBT performed better in elevating IgG levels (MD 3.75; 95% CrI [0.25, 7.74]) than CBT alone.

### 3.5 Analysis of cumulative probability results for different TCM decoctions in treating pediatric asthma

**FIGURE 9:**
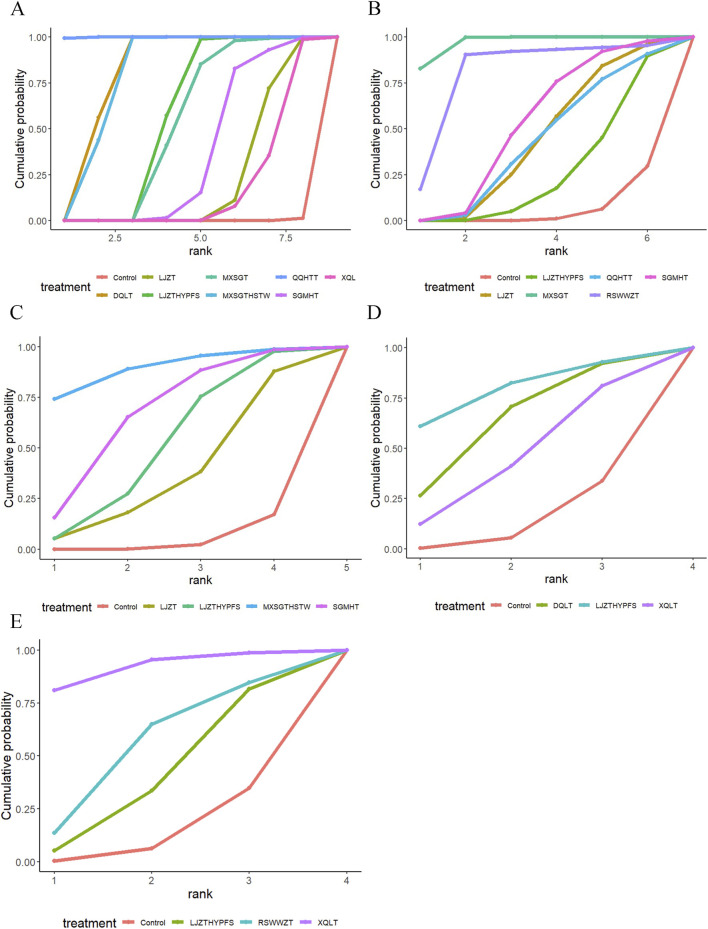
SUCRA plots for outcome measures of TCM decoctions in treating pediatric asthma **(A)** FEV1 **(B)** PEF **(C)** FVC **(D)** IgE **(E)** IgG.

According to the SUCRA values of different decoctions, QQHTT + CBT (SUCRA = 99.9%) ranked highest for FEV1. MXSGT + CBT (SUCRA = 97.1%) ranked highest for PEF. MXSGTHSTW + CBT (SUCRA = 89.4%) ranked highest for FVC. LJZTHYPFS + CBT (SUCRA = 78.8%) ranked first in the potential to reduce IgE. XQLT + CBT (SUCRA = 91.6%) ranked highest for IgG (Table 1; Figure 9).

**TABLE 1:** Efficacy ranking of different decoctions for treating pediatric asthma across outcome measures.

Treatment	FEV1		PEF		FVC		IgE		IgG	
	SUCRA/%	Rank	SUCRA/%	Rank	SUCRA/%	Rank	SUCRA/%	Rank	SUCRA/%	Rank
Control	0.18	9	6.22	7	4.87	5	13.19	4	13.9	4
DQLT	82.05	2	NR	-	NR	-	63.13	2	NR	-
LJZT	22.96	7	44.14	4	37.32	4	NR	-	NR	-
LJZTHYPFS	57.02	4	26.42	6	51.43	3	78.82	1	40.1	3
MXSGT	52.98	5	97.11	1	NR	-	NR	-	NR	-
MXSGTHSTW	80.50	3	NR	-	89.40	1	NR	-	NR	-
QQHTT	99.93	1	42.80	5	NR	-	NR	-	NR	-
RSWWZT	NR	-	80.52	2	NR	-	NR	-	54.4	2
SGMHT	36.57	6	52.79	3	66.97	2	NR	-	NR	-
XQLT	17.81	8	NR	-	NR	-	44.86	3	91.6	1

NR: not reported; DQLT, Da Qing Long Tang; LJZT, Liu Jun Zi Tang; LJZTHYPFS, Liu Jun Zi Tang He Yu Ping Feng San; MXSGT, Ma Xing Shi Gan Tang; MXSGTHSTW, Ma Xing Shi Gan Tang He Su Ting Wan; QQHTT, Qing Qi Hua Tan Tang; RSWWZT, Ren Shen Wu Wei Zi Tang; SGMHT, She Gan Ma Huang Tang; XQLT, Xiao Qing Long Tang. In sensitivity analysis 1 (including standard decoctions only), QQHTT could not be evaluated because the only study included used a modified decoction. For FEV_1_, DQLT and MXSGTHSTW then maintained the top ranks (Supplementary Table S7).

### 3.6 Consistency and publication bias in studies on TCM decoctions for treating pediatric asthma

**FIGURE 10:**
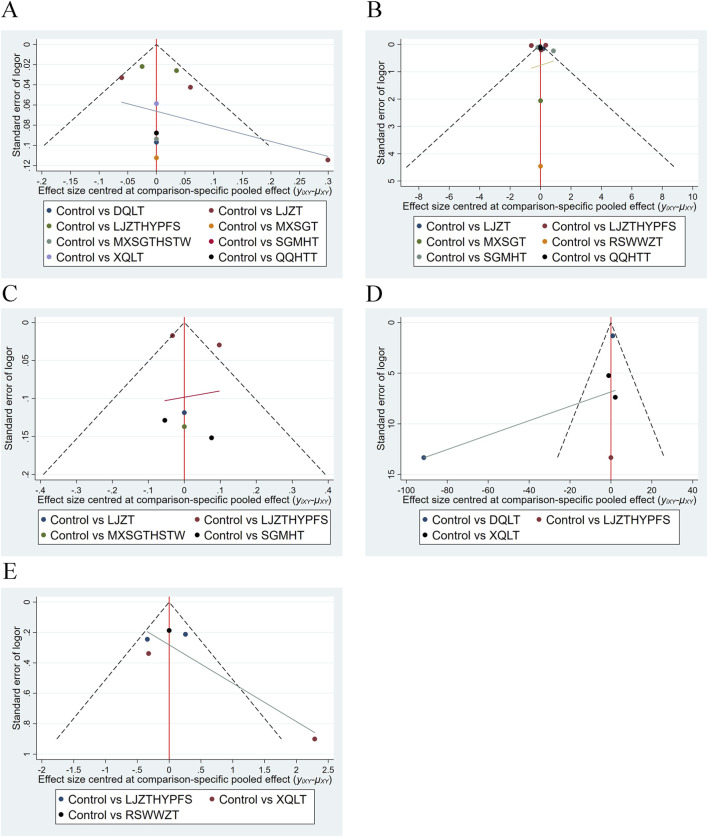
Funnel plots for outcome measures of TCM decoctions in treating pediatric asthma. **(A)** FEV1; **(B)** PEF; **(C)** FVC; **(D)** IgE; **(E)** IgG.

As noted in the Methods section, no closed loops were present. Comparison-adjusted funnel plots were generated for each outcome measure. The dots represented the included studies (Figure 10). The funnel plots were asymmetrical, with some dots lying outside or below the 95% CrI. This suggested potential publication bias or small-study effects among the included studies.

### 3.7 Sensitivity analysis

#### 3.7.1 FEV1

The estimated effects (MDs) for FEV_1_ were highly consistent across the three analyses. For the six treatments with data in all three analyses (DQLT, LJZT, LJZTHYPFS, MXSGTHSTW, SGMHT, and XQLT), the point estimates and CIs for the MDs showed virtually no variation (Supplementary Table S7). The SUCRA ranking showed that the top three interventions, QQHTT, DQLT, and MXSGTHSTW, consistently ranked in the top three across all analyses (Supplementary Table S8). QQHTT ranked first in both the main analysis (SUCRA = 99.9%) and sensitivity analysis 2 (99.9%). Its efficacy could not be estimated in sensitivity analysis 1 because the only study involving QQHTT used a modified decoction and was therefore excluded under the strict inclusion criteria for standard decoctions. This suggested that QQHTT exhibited some stable effects in improving FEV_1_. The slight changes in the ranking of DQLT and MXSGTHSTW (swapping first and second place in sensitivity analysis 1) did not affect the overall conclusion that these three decoctions are relatively effective in improving FEV_1_.

#### 3.7.2 PEF

Compared with FEV_1_, the number of eligible treatments for PEF varied significantly across analyses. Particularly in sensitivity analysis 1, only three treatments had estimable data (LJZT, LJZTHYPFS, SGMHT) (Supplementary Table S7). Therefore, the SUCRA rankings of the treatments retained in sensitivity analysis 1 were artificially inflated due to the lack of competing treatments (e.g., LJZTHYPFS ranked first with a SUCRA of 86.7%) (Supplementary Table S8), which does not reflect a true improvement in relative efficacy. MXSGT + CBT consistently ranked first in the main analysis (SUCRA = 97.1%) and sensitivity analysis 2 (SUCRA = 93.5%), while RSWWZT + CBT maintained the second position in both analyses, supporting the stability of this finding.

#### 3.7.3 FVC

The two treatments, MXSGTHSTW and SGMHT, had complete data in all three analyses, and the estimated MDs were identical or nearly identical across analyses. For instance, MXSGTHSTW had an MD of 0.72 in all three analyses (Supplementary Table S7) and completely consistent SUCRA rankings (Supplementary Table S8). MXSGTHSTW + CBT ranked first in all three analyses (SUCRA range: 89.4%–98.1%), followed by SGMHT + CBT (SUCRA range: 62.0%–67.0%). These findings indicated high stability in the conclusions regarding FVC.

#### 3.7.4 IgE

The IgE outcome exhibited high sensitivity to changes in analysis conditions. DQLT ranked second in the main analysis (SUCRA = 63.1%), first in sensitivity analysis 1 (94.6%), and third in sensitivity analysis 2 (52.7%). LJZTHYPFS ranked first in the main analysis (78.8%), second in sensitivity analysis 1 (72.1%), and first in sensitivity analysis 2 (65.3%) (Supplementary Table S8). Although these two treatments consistently maintained the top two positions, their ranking order was unstable. This instability may be attributed to sensitivity analysis 1, in which the reduced number of eligible treatments artificially inflated the SUCRA value for DQLT. Furthermore, no treatment showed a statistically significant difference compared to CBT alone in any analysis (Supplementary Table S7). These results suggest that the evidence regarding IgE reduction remains uncertain.

#### 3.7.5 IgG

The IgG outcome exhibited the highest stability among all outcomes. XQLT + CBT consistently ranked first in all three analyses, with SUCRA values of 91.6% (main analysis), 100% (sensitivity analysis 1), and 91.6% (sensitivity analysis 2). RSWWZT + CBT consistently ranked second (SUCRA: 54.4%, 50.0%, and 54.4%, respectively) (Supplementary Table S8). XQLT also showed consistent MD estimates (range: 3.70–5.40) with overlapping CIs (Supplementary Table S7).

#### 3.7.6 Model convergence and fit

All models across all analyses demonstrated sufficient convergence (Supplementary Material 2), with R-hat <1.05 for all parameters. DIC values varied across analyses, with some differences exceeding 5, particularly the difference between sensitivity analysis 1 and the main analysis. These differences were attributed to the significant reduction in sample size in sensitivity analysis 1, rather than a violation of the consistency assumption, as confirmed by the stable model convergence diagnostics.

### 3.8 Safety and adverse reactions

Among the 21 included RCTs, 33% (7/21) reported adverse reactions. Of these 7 RCTs, 50% (4/7) documented general adverse reactions, with no statistically significant differences between the two groups. As Rong Lu et al. (Zhang, 2024). Noted, no evident adverse reactions were observed in either group. The control group reported 2 cases of mild headache, while the observation group had 1 case of mild headache and 2 cases of transient palpitations, all of which improved without special intervention. The difference in the incidence of adverse reactions between the two groups, 5.88% (2/34) versus 8.57% (3/35), was not statistically significant (χ^2^ = 0.186, P = 0.667).

## 4 Discussion

Pediatric asthma is increasingly prevalent worldwide, imposing a heavy burden (Ferrante and La Grutta, 2018). TCM treatment is stage-based and addresses underlying deficiencies in the lung, spleen, and kidney alongside manifest symptoms (Zhang et al., 2023; Li and Zhu, 2012). CBT commonly involves medications to control the disease, such as inhaled corticosteroids (Zhao et al., 2015; Laurence and Brunton, 2011; Asthma GIf, 2021). TCM demonstrates significant efficacy in alleviating symptoms, reducing asthma attacks, and lowering the dose of conventional medications, with particularly evident effects during consolidation treatment in the remission phase (Zhang et al., 2024). Therefore, this NMA included 21 RCTs to evaluate the comprehensive efficacy (level of disease control, improvement of pulmonary function, and reduction of recurrence rate) and safety of 9 commonly used TCM regimens for treating pediatric asthma. It was intended to guide the combination of TCM and biomedicines to optimize clinical medication strategies for pediatric asthma.

The analysis results indicated that compared to CBT alone, QQHTT + CBT (SUCRA = 99.9%) ranked first in the probability of being the optimal intervention for increasing FEV1. MXSGTHSTW + CBT (SUCRA = 89.4%) ranked first in the probability of being the optimal intervention for improving FVC. LJZTHYPFS + CBT (SUCRA = 78.8%) ranked first in the probability of being the optimal intervention for reducing IgE. XQLT + CBT (SUCRA = 91.6%) ranked first in the probability of being the optimal intervention for increasing IgG. MXSGT + CBT (SUCRA = 97.1%) ranked first in the probability of being the optimal intervention for increasing PEF.

QQHTT + CBT demonstrated the potentially best efficacy in increasing FEV1. A 2024 review by Luo et al. also noted that QQHTT used to treat asthma could enhance FEV1 levels (Wang L. et al., 2024). The conclusion of our study (QQHTT + CBT may be optimal for improving FEV1) differs from the conclusion of a 2022 meta-analysis by Zhang et al. (Zhang et al., 2022) on acute asthma attacks in children, which concluded that MXSGT is optimal. This discrepancy does not represent a contradiction but stems from differences in the disease stage and TCM syndrome patterns among the patients included in the two studies. Zhang et al.'s study explicitly focused on acute asthma attacks, where the core pathogenesis is the stagnation of qi in the lung. The key to treatment lies in rapidly dispersing lung qi, relieving asthma, and resolving bronchospasm. Thus, MXSGT, with ephedra as the monarch botanical drug, demonstrates outstanding efficacy in rapidly improving FEV1. In contrast, the articles included in our study likely encompassed more patients in the chronic persistent stage or protracted stage, where the core pathogenesis involves phlegm-heat obstructing the lung. In such patients, the primary pathological manifestations are chronic airway inflammation and obstruction by mucus. For this condition, QQHTT, employing the method of clearing heat and resolving phlegm, offers a sustained effect by reducing inflammation and phlegm secretion, thereby substantially improving baseline FEV1 levels. This contrast precisely corroborates treatment based on syndrome differentiation in TCM. MXSGT excels at treating the acute obstruction of qi, while QQHTT is good at clearing chronic phlegm. Each decoction addresses distinct clinical stages of asthma, collectively forming a complete treatment regimen. QQHTT, primarily composed of *Scutellaria baicalensis* roots, *Trichosanthes kirilowii* seeds, *Citrus reticulata* peel, and *Glycyrrhiza uralensis*, is good at clearing heat, resolving phlegm, dispersing lung qi, and relieving wheezing. Modern research has indicated that baicalin exhibits significant anti-inflammatory and immunomodulatory properties. By inhibiting the signaling pathways of c-Jun N-terminal kinase (JNK/c-Jun), Toll-like receptor 4 (TLR4), and nuclear factor-κB (NF-κB), it reduces macrophage activation, thereby alleviating pulmonary inflammation. This mechanism relieves bronchospasm, improves airway ventilation function (Ge, 2022), and enhances FEV1 values. Glycyrrhizin in *Glycyrrhiza uralensis* (Qi et al., 2023; Hocaoglu et al., 2011) inhibits the signaling pathways of NF-κB and signal transducer and activator of transcription 6 (STAT6), reduces the adhesion of eosinophils and the synthesis of IL-5, and thus alleviates airway inflammation. Its antitussive and expectorant effects help maintain airway moisture and reduce irritation. By directly targeting the release of inflammatory mediators and airway hyperresponsiveness, it serves as an excellent prescription for alleviating pulmonary ventilation impairment in the acute phase (Zhu, 2020).

MXSGT + CBT was potentially the most effective in improving PEF. MXSGT uses gypsum as the monarch botanical drug to clear lung heat, ephedra as the minister botanical drug to disperse lung qi and relieve wheezing, and apricot kernel and *Glycyrrhiza uralensis* as assistant botanical drugs to relieve coughing and resolve phlegm, representing a basic prescription for treating lung-heat cough and asthma. The ephedra-apricot kernel pair within the decoction relaxes airway smooth muscle by increasing cAMP levels and upregulating the cAMP/cGMP ratio (Li et al., 2023). It rapidly elevates PEF and thus improves the patency of proximal large airways (Yuan et al., 2021; Pellegrino et al., 2005). The synergistic combination of plant metabolites rapidly alleviates spasm and narrowing of the central airway, resulting in higher airflow velocity during early expiration. This increased airflow velocity directly manifests as a significant increase in PEF values and is clinically important for monitoring and alleviating the acute symptoms and daytime variations of symptoms in children with asthma.

Regarding secondary outcomes, MXSGTHSTW + CBT was potentially the most effective in enhancing FVC. This combination relaxes bronchial smooth muscle via the cyclic adenosine monophosphate (cAMP)-protein kinase A (PKA)-cAMP response element-binding protein (CREB) pathway. Furthermore, the cardiac glycoside metabolites of *Semen lepidii* enhance pulmonary circulation and reduce pulmonary edema, thereby elevating FVC (Kim et al., 2018; Hwang et al., 2021; Wang H. H. et al., 2020; Bejček et al., 2021; Pietrobon, 2002; Fuyuno et al., 2018; Tian et al., 2014; Statler and Kohli, 2025; Lic et al., 2018). It should be noted that the NMA did not show statistically significant differences in IgE reduction for any decoction compared to CBT alone. However, based on the relative ranking (SUCRA), LJZTHYPFS + CBT may show relatively the greatest potential in reducing serum IgE, which is hypothesized to be related to its immunomodulatory effects. This decoction strengthens the spleen, consolidates the foundation, and regulates immune responses, potentially aiding in allergy control by inhibiting inflammatory pathways such as NF-κB (Liu and Lu, 2021; Tao et al., 2017; Kale et al., 2010; Qin et al., 2025; He et al., 2025; Du et al., 2021). Nonetheless, this ranking should be interpreted with caution in the absence of statistical significance, and further research is needed to confirm this effect. XQLT + CBT was likely the best in elevating serum IgG levels. Its active metabolites, such as the 2-undecanone from *Pinellia ternata*, alleviate oxidative stress and inflammation by activating the nuclear factor erythroid 2-related factor 2 (Nrf2) - heme oxygenase-1 (HO-1)/quinone oxidoreductase 1 (NQO-1) pathway, thereby enhancing humoral immunity (Zhu et al., 2021).

The sensitivity analyses conducted in this study provided important evidence to support the stability of the main analysis results. For FVC and IgG, the top-ranked interventions (MXSGTHSTW + CBT and XQLT + CBT, respectively) maintained their positions across all three analyses, suggesting high reliability of these results. For FEV_1_, the top three decoctions (QQHTT, DQLT, and MXSGTHSTW) consistently ranked at the forefront, although their relative order showed slight sensitivity to the inclusion of decoctions with modifications. This suggested that while the exact ranking among these three may depend on specific formulations, it also supported the finding that they were indeed superior to other decoctions in improving FEV_1_.

For PEF, the apparent instability in rankings observed in sensitivity analysis 1 was caused by a significant reduction in the number of eligible treatments, which artificially inflated the SUCRA values of the remaining interventions. This finding underscores the importance of interpreting sensitivity analyses in the context of data availability, rather than relying solely on ranking order. For IgE, the absence of statistically significant differences across all analyses, coupled with unstable rankings, suggests that the immunomodulatory effects of TCM decoctions on IgE levels remain uncertain and need to be further investigated.

Differences in DIC across analyses (exceeding 5 for some comparisons) were attributed to variations in sample size and data sparsity, rather than violations of model assumptions. Consistently excellent convergence diagnostics across all analyses (R-hat <1.05 for all) supported this explanation, confirming that the Markov Chain Monte Carlo chains were stable and well-mixed in all analyses.

To our knowledge, this meta-analysis is the first to comprehensively evaluate TCM decoctions by comparing the effects of different TCM decoctions on inflammatory and immune responses in pediatric asthma. This study provides important references for developing optimal treatment strategies for pediatric asthma. However, certain limitations remain. First, the methodological quality of included studies is suboptimal. Most studies did not specify the detailed randomization process, and allocation concealment and blinding were universally unreported. We acknowledge that this severely limits the strength of the evidence. However, excluding all such studies would have resulted in zero eligible trials and introduced selection bias. We therefore retained them but prioritized objective outcome measures (spirometry, laboratory tests), which are less susceptible to bias. Second, the comparability of TCM decoctions across studies is a key concern. Although we assumed that decoctions sharing the same name represent comparable interventions, variations in composition due to syndrome differentiation could potentially violate the transitivity assumption. Our sensitivity analysis indicates that pooling modified and standard decoctions may bias estimates for some specific outcomes. Therefore, our findings should be interpreted as exploratory, and future head-to-head RCTs using strictly standardized decoctions with detailed phytochemical characterization are needed. Third, all included RCTs had short intervention cycles (primarily acute-phase treatment), lacking long-term follow-up data on consolidation therapy in the remission phase. Therefore, it was impossible to evaluate the effects of TCM decoctions on the long-term recurrence rate of asthma, progressive decline in lung function, cumulative dosage of steroids, and safety outcomes (e.g., incidence of adverse reactions). Fourth, the SUCRA-based ranking results should be interpreted with extreme caution. The NMA contained no closed loops for any outcome, indicating that there was no opportunity to compare direct and indirect evidence for any comparison. In addition, some interventions were reported in only one or two small-scale studies, which significantly limits the stability and reliability of the rankings. The rankings provided in this study are therefore preliminary and exploratory only and should not be used as the sole basis for clinical decision-making. Further large-scale head-to-head RCTs are needed to confirm the relative efficacy of different TCM decoctions. Finally, all included studies were Chinese studies, which restricts the applicability of the findings to other populations.

The findings of this NMA, while constrained by the methodological limitations of the primary evidence, delineate several priority areas for future investigation. First, the SUCRA-based probability rankings, particularly those suggesting differential advantages of specific decoctions for distinct clinical endpoints, require validation through large-scale, multicenter, double-blind RCTs. Second, the absence of statistically significant IgE reduction across all interventions underscores the need for mechanistic studies to elucidate whether the immunomodulatory effects of TCM decoctions in pediatric asthma operate primarily through pathways independent of IgE-mediated allergy. Third, the pronounced heterogeneity in intervention duration observed among included studies (ranging from 3 days to 6 months) highlights a critical knowledge gap: the optimal timing and duration of adjunctive TCM therapy relative to disease stage (acute exacerbation versus remission). Finally, as the field moves toward precision medicine, future research should incorporate multi-omics approaches to identify biomarkers predictive of differential response to specific TCM decoctions. Such efforts would not only strengthen the evidence base for integrative pediatric asthma management but also advance the broader goal of evidence-based personalization in traditional medicine.

## 5 Conclusion

In summary, this NMA suggests that the combination of TCM decoctions with CBT holds potential for improving lung function and immune markers in children with asthma. The main analysis identified QQHTT + CBT and MXSGT + CBT as the most effective interventions for improving FEV_1_ and PEF, respectively. Sensitivity analyses supported the stability of the results for FVC (for which MXSGTHSTW + CBT consistently ranked first) and IgG (for which XQLT + CBT consistently ranked first). For FEV_1_, the top three decoctions (QQHTT, DQLT, and MXSGTHSTW) maintained their leading positions in both the main analysis and sensitivity analysis 2. In sensitivity analysis 1, QQHTT could not be estimated because only standard decoctions were included, but DQLT and MXSGTHSTW maintained the top two positions. Despite minor fluctuations in rankings, the findings remain reasonably reliable. Regarding IgE, no decoction achieved a statistically significant reduction in IgE, warranting further investigation. These findings provide a valuable evidence base for personalized TCM treatment of pediatric asthma. However, because of serious methodological limitations in the included studies—particularly the absence of blinding and allocation concealment, coupled with unresolved heterogeneity—the current findings represent only preliminary, hypothesis-generating results. They do not yet provide a basis for clinical recommendations. Given this, the most important conclusion of this study is not to endorse any particular decoction, but rather to highlight the urgent need for multicenter, large-scale, rigorously designed RCTs that use strictly standardized and chemically characterized TCM decoctions in the future. These trials must include comprehensive chemical characterization of the decoctions and strict blinding to confirm the true efficacy of any TCM decoction in treating pediatric asthma.

## Data Availability

The original contributions presented in the study are included in the article/Supplementary Material, further inquiries can be directed to the corresponding author.
