# Supplementary material for: Efficacy of traditional Chinese medicine decoctions combined with conventional therapy for pediatric asthma: a network meta-analysis
Source: Front Pharmacol. 2026 Jun 25;17:1780354. doi: 10.3389/fphar.2026.1780354 (PMC13346251; doi:10.3389/fphar.2026.1780354)
Supplement: Supplementary file 1 [file Supplementaryfile1.pdf]

ConPhyMP checklist of information for reporting plant material and its initial processing [1,2] (relevant for all studies on medicinal and food plants including extract types A, B, and C)

SECTION/TOPIC	ITEM NO.	CHECKLIST ITEM	YES	NO	NOT APPLICABLE	PAGE NO., IF ANY
Title and abstract	1	A clear and concise title including an informative abstract and balanced summary.	✓			Page 1-2
Description of the botanical drug and taxonomic authentication	2	Botanical or morphological authentication of the plant material (desirable is a combination with DNA barcoding, e.g., PCR, RFLP, genome sequencing) and the information must be included in a separate section of Material and Methods, if applicable, combined with the information required under item 3:	✓			ary Table S4
Description of the extract and extraction process	3	A separate section in Material and Methods, covers the relevant information on the material investigated, including the full species name(s), authorites and family; e.g. *Salvia miltorrhiza* Bunge [Lamiaceae; *Salviae miltorrhizae radix et rhizoma*], and on the processing and extraction of the crude drug including the traditional processing of the material used medicinally (fumigation, steaming, roasting, cooking, frying, etc.).	✓			Tables S3-S4
Documentation of the legal basis for collection and processing	4	Full compliance with the Nagoya protocol, CITES, and all associated treaties including phytosanitary regulations.	✓			Page 8
Description of product characteristics, in case of a finished (commercial) product	5	Information on the characteristics of the commercial products including batch number and date of production/best by information and regulatory status.			✓	N/A(secondar

Note: Please also include here the following information about your submitted manuscript:

Name of the journal:	Frontiers in Pharmacology
Date of the enquiry:	2026-04-16
Title of the manuscript:	Efficacy of Traditional Chinese Medicine Decoctions Combi
List of the authors:	Yan Zhao, Meina Li, Ai Ai, Xin He, Xingyu Liu, Weiwei Tian, Yi

[1] Please acknowledge/cite this as follows: Heinrich M, Jalil B, Abdel-Tawab M, Echeverria J, Kulić Ž, McGaw LJ, et al. Best Practice in the chemical characterisation of extracts used in pharmacological and toxicological research—The ConPhyMP—Guidelines. Frontiers in Pharmacology. 2022;13:953205. https://doi.org/10.3389/fphar.2022.953205

[2] We strongly recommend reading this checklist in conjuncton with ConPhyMP 2022 explanaton and elaboraton for important clarifications on all items. If relevant, we also recommend after reading Heinrich et al. (2020) Best practice in research—Overcoming common challenges in phytopharmacological research. Journal of Ethnopharmacology. 2020;246:112230. https://doi.org/10.1016/j.jep.2019.112230

Supplementary Table S2 ConPhyMP-checklists-table-2a

ConPhyMP checklist of items for conducting and reporting analytical methods[1,2] relevant for extract type A (for species or botanical drugs covered in a monograph in one of the national or regional pharmacopoeias)

SECTION/TOPIC	ITEM NO.	CHECKLIST ITEM	YES	NO	NOT APPLICABLE	PAGE NO., IF ANY
Type of extract	1	A – Confirm that the species or botanical drug under investigation is covered in a monograph in one of the national or regional pharmacopoeias.	✓			s S3 and S4.
Preferred/main methods for extract characterisation/ chemical analysis	2	Compliance with pharmacopoeial standards to be followed:				
		(a) The description of the active ingredients in the botanical drug (if known) or analytical marker compounds as defined.	✓			arks column.
		(b) An analysis as defined in the monograph is needed if the extract has not been supplied with a certificate.			✓	s conducted.
		(c) If the preparation was purchased, the manufacturer and certificate of analysis need to be included.			✓	al products.
		Including either the preferred or alternative approaches for characterisation:				
		(a) Triple chemical fingerprinting methods, each with one or more detection parameters.		✓		discussion.
		(b) Quantification of at least two marker compounds (unless this is not feasible, evidence needs to be provided), and justification of the choice of markers (if applicable).		✓		arch. Ditto.
Alternative methods for extract characterisation/chemical analysis	3	(a) Single chemical fingerprinting method with at least three different detection parameters (i.e., altered detection parameters, like TLC/ HPTLC with different staining reagents and/or UV excitation wave-lengths, HPLC-DAD/LCDAD with different wavelengths). The same applies to coupling MS or NMR to chromatographic techniques.		✓		al research.
		(b) Quantification of at least two marker compounds (unless this is not feasible, evidence needs to be provided), and justification of the choice of markers (if applicable).		✓		al research.
Use of reference standards	4	(a) Direct overlay of the chromatogram of the sample with that of an officially specified reference standard (if applicable).			✓	a available.
		(b) Chromatographic fingerprinting: Direct overlay of the chromato-gram of the sample with that of official reference standards of the powdered plant material or the dry extract from the plant material.				
Comparison of different extracts/ samples of the same plants	5	(a) Direct comparison of the chromatographic/spectroscopic system and/or scoring system for "similarity" to be followed.			✓	a available.

Note: Please also include here the following information about your submitted manuscript:

Name of the journal:	Frontiers in Pharmacology
Date of the enquiry:	2026-04-17
Title of the manuscript:	Efficacy of Traditional Chinese Medicine Decoctions Combi
List of the authors:	Yan Zhao, Meina Li, Ai Ai, Xin He, Xingyu Liu, Weiwei Tian, Yi

[1] Please acknowledge/cite this as follows: Heinrich M, Jalil B, Abdel-Tawab M, Echeverria J, Kulić Ž, McGaw LJ, et al. Best Practice in the chemical characterisation of extracts used in pharmacological and toxicological research—The ConPhyMP—Guidelines. Frontiers in Pharmacology. 2022;13:953205. https://doi.org/10.3389/fphar.2022.953205

[2] We strongly recommend reading this checklist in conjuncton with ConPhyMP 2022 explanaton and elaboraton for important clarifications on all items. If relevant, we also recommend after reading Heinrich et al. (2020) Best practice in research—Overcoming common challenges in phytopharmacological research. Journal of Ethnopharmacology. 2020;246:112230. https://doi.org/10.1016/j.jep.2019.112230

Supplementary Table S3 Standard Composition, Key Metabolites, and Details of Original Studies Reporting the Included TCM Decoction

Formula Name	Standard Composition	Key Marker metabolites	Core ingredients (monarch and minister herbs)	Study ID	Modifications (Additions/Subtractions),	Are core ingredients retained	Consistency of the Reported Formula with the Standard Prescription
Da Qing Long Tang (DQLT)	*Ephedra Herb, Cinnamon Twig, Licorice Root, Bitter Apricot Seed, Gypsum, Fresh Ginger, Jujube Fruit*	ephedrine, pseudoephedrine, cinnamaldehyde, liquiritin, glycyrrhizic acid, amygdalin, calcium sulfate dihydrate, and 6-gingerol.	Monarch herb: *Ephedra Herb*; minister herb: *Cinnamon twig, Gypsum*	1	No modification	Y	100%(3/3)
				2	No modification	Y	
				3	Modifications based on syndrome differentiation and treatment are detailed in Supplementary Table S2.	Y	
Liu Jun Zi Tang He Yu Ping Feng San (LJZTHYPFS)	*Tangerine Peel, Pinellia Tuber, Poria, Licorice Root, Ginseng, White Atractylodes Rhizome, Saposhnikovia Root, Astragalus Root*	Liquiritin, Glycyrrhizic Acid, Hesperidin, Poria β-(1→3)-Glucan (expressed as anhydrous glucose), Ginsenoside Rg1, Ginsenoside Re, Ginsenoside Rb1, Prim-O-glucosylcimifugin, Cimifugin, 3′-O-Angeloylhamaudol, Calycosin-7-O-β-D-glucoside	Monarch herb: *Ginseng, Astragalus Root*; minister herb: *White Atractylodes Rhizome*	4	No modification	Y	100%(6/6)
				5	Modifications based on syndrome differentiation and treatment are detailed in Supplementary Table S2.	Y	
				6	No modification	Y	
				7	Modifications based on syndrome differentiation and treatment are detailed in Supplementary Table S2.	Y	

				11	Modifications based on syndrome differentiation and treatment are detailed in Supplementary Table S2.	Y	
				24	No modification	Y	
Liu Jun Zi Tang (LJZT)	*Tangerine Peel, Pinellia Tuber, Poria, Licorice Root, Ginseng, White Atractylodes Rhizome*	Liquiritin, Glycyrrhizic Acid, Hesperidin, Poria β-(1→3)-Glucan (expressed as anhydrous glucose), Ginsenoside Rg1, Ginsenoside Re, Ginsenoside Rb1	Monarch herb: *Ginseng*; minister herb: *White Atractylodes Rhizome*	8	No modification	Y	100%(3/3)Since Study 10 was not included in the main analysis, it was excluded from the calculations.
				9	No modification	Y	
				10	Replaced Ginseng with Pseudostellaria Root.	N	
Ma Xing Shi Gan Tang (MXSGT)	*Ephedra Herb, Bitter Apricot Seed, Licorice Root, Gypsum*	Ephedrine, Pseudoephedrine, Liquiritin, Glycyrrhizic Acid, Amygdalin, Calcium sulfate dihydrate	Monarch herb: *Ephedra Herb, Gypsum*; minister herb: *Bitter Apricot Seed*	12	Modifications based on syndrome differentiation and treatment are detailed in Supplementary Table S2.	Y	100%(1/1)
Ma Xing Shi Gan Tang He Su Ting Wan (MXSGTHSTW)	*Ephedra Herb, Bitter Apricot Seed, Licorice Root, Gypsum, Semen Lepidii, Perilla Fruit*	Ephedrine, Pseudoephedrine, Liquiritin, Glycyrrhizic Acid, Amygdalin, Calcium sulfate dihydrate, Quercetin-3-O-β-D-glucopyranosyl-7-O-β-D-gentiobioside, Rosmarinic acid	Monarch herb: *Ephedra Herb, Gypsum, Perilla Fruit*; minister herb: *Bitter Apricot Seed, Semen lepidii*	13	No modification	Y	100%(1/1)

Ren Shen Wu Wei Zi Tang (RSWWZT)*	*Ginseng, White Atractylodes Rhizome, Poria, Schisandra Fruit, Ophiopogon Root, Licorice Root*	Liquiritin, Glycyrrhizic Acid, Poria β-(1→3)-Glucan (expressed as anhydrous glucose), Ginsenoside Rg1, Ginsenoside Re, Ginsenoside Rb1, Schisandrin, Ophiopogon Total Saponins	Monarch herb: *Ginseng*; minister herb: *White Atractylodes Rhizome*	15	Added Pinellia Tuber, Tangerine Peel.	Y	50%(1/2) Since Study 17 was not included in the main analysis, it was excluded from the calculations.
				16	No modification	Y	
				17	Added Medicated Leaven 15 g, Barley Malt 15 g, Goji Berry 15 g; removed White Atractylodes Rhizome.	N	
She Gan Ma Huang Tang (SGMHT)	*Belamcanda Rhizome, Ephedra Herb, Fresh Ginger, Asarum Herb, Aster Root, Coltsfoot Flower, Jujube Fruit, Pinellia Tuber, Schisandra Fruit*	Ephedrine, Pseudoephedrine, 6-Gingerol, Schisandrin, Irisflorentin, Asarinin, Shionone, Tussilagone	Monarch herb: *Belamcanda Rhizome, Ephedra Herb*; minister herb: *Asarum Herb*, *Pinellia Tuber.*	18	Added Perilla Fruit. Modifications based on syndrome differentiation and treatment are detailed in Supplementary Table S2.	Y	50%(1/2)
				19	No modification	Y	
Xiao Qing Long Tang (XQLT)	*Ephedra Herb, White Peony Root,*	Ephedrine, Pseudoephedrine, Cinnamaldehyde, Liquiritin,	Monarch herb: *Ephedra Herb, Cinnamon Twig*; minister herb: *Dried Ginger*	21	No modification	Y	66.7%(2/3)Since Study 20
				22	Modifications based on	Y	

	Asarum Herb, Dried Ginger, Licorice Root, Cinnamon Twig, Schisandra Fruit, Pinellia Tuber	Glycyrrhizic Acid, Schisandrin, Asarinin, Paeoniflorin, 6-Gingerol			syndrome differentiation and treatment are detailed in Supplementary Table S2.		was not included in the main analysis, it was excluded from the calculations.
				23	No modification	Y	
				20	Added Cinnamon Twig, removed Platycodon Root.	N	
Qing Qi Hua Tan Tang (QQHTT)*	*Tangerine Peel, Bitter Apricot Seed, Immature Bitter Orange, Scutellaria Root, Trichosanthes Seed, Poria, Bile Arisaema, Pinellia Tuber*	Amygdalin, Hesperidin, Poria β-(1→3)-Glucan (expressed as anhydrous glucose), Synephrine, Baicalin, 3,29-Dibenzoylkarounitriol	Monarch herb: *Bile Arisaema*; minister herb: *Trichosanthes Seed, Scutellaria Root, Pinellia Tuber*	14	Replaced Immature Bitter Orange with Bitter Orange; added Thunberg Fritillary Bulb, Charred Hawthorn Fruit, Areca Peel, Licorice Root.	Y	0%(0/1)

Notes: Except for the compound decoctions marked with an asterisk (*), the standard compositions of all formulations are derived from the *Formulas of Chinese Medicine* (10th Edition), the national "14th Five-Year Plan" textbook for higher education. (RSWWZT)* is sourced from *Youyou Jicheng* (A Compilation of Pediatric Essentials) by Chen Fuzheng, Qing Dynasty; (QQHTT)* is sourced from *Yifang Kao* (An Examination of Medical Formulas) by Wu Kun, Ming Dynasty. In clinical practice, Codonopsis pilosula (Dangshen) is routinely substituted for Panax ginseng (Renshen).

Supplementary Table S4 Taxonomic authentication information of the included medicinal materials

Chinese Pinyin	Latin binomial name of the drug*	Botanical scientific name**	Pharmacopoeial official part used
Ma huang	*Ephedrae Herba*	*Ephedra sinica Stapf/Ephedra intermedia Schrenk & C.A.Mey./Ephedra equisetina Bunge.*	Dried herbaceous stem
Gui zhi	*Cinnamomi Ramulus*	*Neolitsea cassia (L.) Kosterm.*	Dried twig
Gan cao	*Glycyrrhizae Radix et Rhizoma*	*Glycyrrhiza uralensis Fisch./Glycyrrhiza inflata Bat./Glycyrrhiza glabra L.*	Dried root and rhizome
Ku xingren	*Armeniacae Amarum Semen*	*Prunus armeniaca L.var.ansu Maxim./Prunus sibirica L./Prunus mandshurica (Maxim.)Koehne/Prunus armeniaca L.*	Dried ripe seed
Shi gao	*Gypsum fibrosum*	N/A	Mineral material
Sheng jiang	*Zingiber officinale Roscoe*	*Zingiber officinale Rosc.*	Fresh rhizome
Gan jiang	*Zingiberis Rhizoma*	*Zingiber officinale Rosc.*	Dried rhizome
Da zao	*Jujubae Fructus*	*Ziziphus jujuba Mill.*	Dried ripe fruit
Chen pi	*Citri Reticulatae Pericarpium*	*Citrus reticulata Blanco*	Dried mature pericarp
Ban xia	*Pinelliae Rhizoma*	*Pinellia ternata (Thunb.) Makino*	Dried tuber
Fu ling	*Poria*	*Poria cocos (Schwein.) F.A.Wolf*	Dried sclerotium
Ren shen	*Ginseng Radix et Rhizoma*	*Panax ginseng C.A. Mey.*	Dried root and rhizome
Bai zhu	*Atractylodis macrocephalae rhizoma*	*Atractylodes macrocephala Koidz.*	Dried rhizome
Fang feng	*Saposhnikoviae Radix*	*Saposhnikovia divaricata(Turcz.ex Ledeb.)Schischk.*	Dried root
Huang qi	*Astragali Radix*	*Astragalus Mongholicus Bunge*	Dried root
Ting lizi	*Descurainiae Semen/Lepidii semen*	*Descurainia sophia(L.)Webb. ex*	Dried ripe seed

		Prantl./Lepidium apetalum Willd.	
Zi suzi	*Perillae fructus*	*Perilla frutescens(L.)Britton*	Dried ripe fruit
Jie zi	*Sinapis Semen*	*Sinapis alba L./× Brassarda juncea (L.) Su Liu & Z.H.Feng*	Dried ripe seed
Lai fuzi	*Raphani Semen*	*Raphanus raphanistrum subsp.sativus(L.)Schmalh.*	Dried ripe seed
Da zaojiao	*Gleditsiae sinensis fructus*	*Gleditsia sinensis Lam.*	Dried ripe fruit
Di long***	*Pheretima;Pheretima aspergillum(E. Perrier)/Pheretima vulgaris Chen/Pheretima guillelmi(Michaelsen)/Pheretima pectinifera Michaelsen**	N/A	Dried body (after removal of viscera)
Jiang can***	*Bombyx Batryticatus; The 4th to 5th instar larvae of Bombyx mori Linnaeus are infected (or artificially inoculated) with Beauveria bassiana (Bals.) Vuillant, resulting in their death.*	N/A	Dried body (after removal of viscera)
Wu weizi	*Schisandrae chinensis fructus*	*Schisandra chinensis (Turcz.) Baill.*	Dried ripe fruit
Mai dong	*Ophiopogonis radix*	*Ophiopogon japonicus(Thunb.)Ker-Gawl.*	Dried root tuber
She gan	*Belamcandae rhizoma*	*Iris domestica (L.) Goldblatt & Mabb.*	Dried rhizome
Xi xin	*Asari radix et rhizoma*	*Asarum heterotropoides F.Schmidt/Asarum sieboldii Miq.*	Dried root and rhizome
Zi wan	*Asteris radix et rhizoma*	*Aster tataricus L.f.*	Dried root and rhizome
Kuan donghua	*Farfarae flos*	*Tussilago farfara L.*	Dried flower bud
Bai shao	*Paeoniae radix alba*	*Paeonia lactiflora Pall.*	Dried root
Dang gui	*Angelicae sinensis radix*	*Angelica sinensis(Oliv.)Diels*	Dried root
Zhi shi	*Aurantii fructus immaturus*	*Citrus × aurantium L./Citrus × aurantium*	Dried immature fruit

		f.aurantium	
Huang qin	*Scutellariae radix*	*Scutellaria baicalensis Georgi*	Dried root
Gua louzi	*Trichosanthis semen*	*Trichosanthes kirilowii Maxim./Trichosanthes rosthornii Harms*	Dried ripe seed
Dan nanxing	*Arisaema cum bile*	*Arisaema amurense Maxim./Arisaema erubescens (Wall.) Schott/Arisaema heterophyllum Blume*	Processed product (prepared from the tuber of Arisaema spp. mixed or fermented with animal bile)
Dan shen	*Salviae miltiorrhizae radix et rhizoma*	*Salvia miltiorrhiza Bunge*	Dried root and rhizome
Tao ren	*Persicae semen*	*Prunus persica(L.)Batsch/Prunus davidiana(Carrière) Franch.*	Dried ripe seed
Chi shao	*Paeoniae radix rubra*	*Paeonia lactiflora Pall./Paeonia veitchii Lynch*	Dried root
Bai zhi	*Angelicae dahuricae radix*	*Angelica dahurica(Fisch. ex Hoffm.)Benth.&Hook. f.ex Franch. & Sav./Angelica dahuricavar. formosana (H.Boissieu) Yen*	Dried root
Shen qu***	*Massa Medicata Fermentata*		Fermented processed product
Shan zhuyu	*Corni fructus*	*Cornus officinalis Siebold & Zucc.*	Dried ripe sarcocarp (with seed removed)
Mai ya	*Hordei fructus germinatus*	*Hordeum vulgare L.*	Germinated dried ripe fruit (caryopsis)
Shan zha	*Crataegi fructus*	*Crataegus pinnatifida var. pinnatifida/Crataegus pinnatifida Bunge*	Dried ripe fruit
Long gu***	*Os Dracons*	*N/A*	Fossilized bone / Fossilized ivory (for "Wuhua Longgu")

Mu li***	*Crassostreae concha;Crassostrea gigas(Thunberg)/Crassostrea talienwhanensis(Crosse)/Crassostrea ariakensis(Wakiya)*	N/A	Dried shell
Zhe beimu	*Fritillariae thunbergii bulbus*	*Fritillaria thunbergii Miq.*	Dried bulb
Chuan beimu	*Fritillariae cirrhosae bulbus*	*Fritillaria cirrhosa D. Don/Fritillaria unibracteata P.K.Hsiao & K.C.Hsia/Fritillaria przewalskii Maxim./Fritillaria delavayi Franch.ex Batalin/Fritillaria taipaiensis P.Y.Li/Fritillaria unibracteata var. wabuensis (S.Y.Tang & S.C.Yueh) Z.D.Liu, Shu Wang & S.C.Chen*	Dried bulb
Yi yiren	*Coicis semen*	*Coix lacryma-jobi L. var. mayuen(Roman.)Stapf*	Dried ripe kernel (with hull and testa removed)
Ji neijin***	*Galli gigerii endothelium corneum;Gallus gallus domesticus Brisson*	N/A	Dried inner lining of the gizzard
Tai zishen	*Pseudostellariae radix*	Pseudostellaria heterophylla*(Miq.)Pax ex Pax*	Dried root tuber
Jin yinhua	*Lonicerae japonicae flos*	Lonicera japonica *Thunb.*	Dried flower bud or the flower with the initial opening
Lian qiao	*Forsythiae fructus*	Forsythia suspensa*(Thunb.)Vahl*	Dried fruit
Gua loupi	*Trichosanthis pericarpium*	Trichosanthes kirilowii *Maxim./*Trichosanthes rosthornii *Harms*	Dried mature pericarp
Hong hua	*Carthami flos*	*Carthamus tinctorius L.*	Dried flower
Gou qizi	*Lycii fructus*	Lycium barbarum *L.*	Dried ripe fruit
Ju hong	*Citri exocarpium rubrum*	Citrus reticulata *Blanco*	Dried exocarp of the mature fruit
Chan	*Cicadae*	N/A	Sloughed

tui***	*periostracum*;Cryptotympana atrata*(Fabricius)*		exoskeleton of the nymph
Jie geng	*Platycodonis radix*	Platycodon grandiflorum*(Jacq.)A. DC.*	Dried root
Niu bangzi	*Arctii fructus*	Arctium lappa *L.*	Dried ripe fruit
Xuan shen	*Scrophulariae radix*	Scrophularia ningpoensis *Hemsl.*	Dried root
Bo he	*Menthae haplocalycis herba*	*Mentha canadensis L.*	Dried aerial parts
Zi suye	*Perillae folium*	Perilla frutescens*(L.)Britton*	Dried leaf
Xin yi	*Magnoliae flos*	Magnolia biondii *Pamp.*/Magnolia denudata *Desr.*/Magnolia sprengeri *Pamp.*	Dried flower bud
Sang baipi	*Mori cortex*	Morus alba *L.*	Dried root bark
Yu xingcao	*Houttuyniae herba*	Houttuynia cordata *Thunb.*	Dried aerial parts
Cang erzi	*Xanthii fructus*	Xanthium sibiricum *Patr.*	Dried ripe fruit with involucre
Bin lang	*Arecae semen*	Areca catechu *L.*	Dried ripe seed

Notes: * Sourced from the Chinese Pharmacopoeia (2025 Edition); ** Sourced from the Medicinal Plant Names Services (MPNS); *** Denotes a mineral, animal-derived drug, or processed product.

Supplementary Table S5 Search Strategy

VIP

Search Strategy	
#1	题名或关键词=(儿童+幼儿+小儿+未成年人+儿科)
#2	题名或关键词=(哮喘+哮病+气喘)
#3	题名或关键词=(汤+散)
#4	摘要=(随机对照+随机分组+随机)
#5	#1AND#2AND#3AND#4

Wanfang

	Search Strategy
#1	题名或关键词=(儿童 OR 幼儿 OR 小儿 OR 未成年人 OR 儿科)
#2	题名或关键词=(哮喘 OR 哮病 OR 气喘)
#3	题名或关键词=(汤 OR 散)
#4	摘要=(随机对照 OR 随机分组 OR 随机)
#5	#1AND#2AND#3AND#4

CNKI

	Search Strategy
#1	主题=(儿童+幼儿+小儿+未成年人+儿科)
#2	主题=(哮喘+哮病+气喘)
#3	主题=(汤+散)
#4	摘要=(随机对照+随机分组+随机)
#5	#1AND#2AND#3AND#4

Pubmed

	Search Strategy
#1	TS=(Child[MeSH Terms]) OR (((Child[Title/Abstract]) OR (Children[Title/Abstract])) OR Search (Pediatrics[Title/Abstract]))
#2	TS=(Asthma[MeSH Terms]) OR (((Asthmas[Title/Abstract]) OR (Asthma[Title/Abstract])) OR (Bronchial Add to History Asthma[Title/Abstract]))
#3	TS=((Decoction[Title/Abstract] OR (tang[THle/Abstract[]) OR (fang[Title/Abstract])
#4	#1AND#2AND#3

Embase

	Search Strategy
#1	TS=(‘child’/exp)OR(‘child’:ab.ti)OR(‘children’:ab.ti)OR (‘pediatrics’:ab.ti)
#2	TS=(‘asthma’/exp)OR(‘asthma’:ab.ti)OR(‘asthmas’:ab.ti)OR (‘asthma,bronchial’:ab.ti)OR(‘bronchial asthma’:ab.ti)

#3	TS=(‘decoction’:ab.ti)OR(‘tang’:ab.ti)OR (‘fang’:ab.ti)
#4	#1AND#2AND#3

Cochrane Library

	Search Strategy
#1	TS=MeSH descriptor:[Child] explode all trees
#2	TS=(Child):ti,ab,kwOR(Children):ti,ab,kwOR(Pediatrics):ti,ab,kw
#3	#1OR#2
#4	TS=MeSH descriptor: [Asthma] explode all trees
#5	TS=(Asthma):ti,ab,kw OR (Asthmas):ti,ab,kw OR (Asthma,Bronchial):ti,ab,kw OR (Bronchial Asthma):ti,ab,kw
#6	#4OR#5
#7	TS=(Decoction):ti,ab,kw OR (tang):ti,ab,kw OR (fang):ti,ab,kw
#8	#3AND#6AND#7

Web of Science

	Search Strategy
#1	TS=(Child) OR TS=(Children) OR TS=(Pediatrics)
#2	TS=(Asthma) OR TS=(Asthmas)OR TS=(Bronchial Asthma)
#3	TS=(Decoction) OR TS=(tang) ORTS=(fang)
#4	#1AND#2AND#3

Supplementary Table S6 Basic characteristics of included studies on TCM decoctions for treating pediatric asthma

	Study	Year	Sample size	Gender (M/F)	Mean age (years)	Asthma staging (severity classification);<br>TCM syndrome differentiation	CBT regimen	Intervention	Total treatment duration (day)	Outcomes
1	HYZhao	2020	DQLT: 34<br>Control: 34	DQLT: 18/16<br>Control: 19/15	DQLT: (6.87±1.70)<br>Control: (6.93±1.72)	NR (mild to moderate; excluding severe cases; specific proportion: NR)<br>TCM syndrome differentiation: Exterior cold with interior heat	All patients received standard oxygen therapy, antiasthmatic drugs, antiallergic agents, antimicrobial drugs, and fluid and electrolyte replacement therapy. Nebulized therapy was administered using a mixture of 250 μg of ipratropium bromide and 0.05 mg of beclomethasone dipropionate suspension per dose, with each session lasting 15 minutes, three times daily.	DQLT comprised 6 g each of ephedra and licorice root, 9 g each of cassia twig, bitter apricot kernel, and fructus jujubae, 20 g of gypsum, and 15 g of ginger. The ingredients were decocted with water to 200 mL. The warm decoction was taken in the morning and evening.	10	F23, F21, F9, F10
2	HRGao	2020	DQLT: 27<br>Control: 27	DQLT:14/13<br>Control:12/15	DQLT:(5.6±1.5)<br>Control:(6.6±1.2)	NR (NR)<br>TCM syndrome differentiation: Exterior cold with interior heat	Oral treatment with erythromycin and azithromycin was administered:	DQLT comprised 6 g of ephedra, 6 g of licorice root, 9 g of cassia twig, 9 g of bitter apricot kernel,	60	F11, F3, F13

							erythromycin 10 mg per dose, twice daily; azithromycin 5 mg per dose, twice daily. Subsequently, nebulized treatment with Ventolin was administered, using 1 mL of Ventolin mixed with 3 mL of 0.9% sodium chloride solution.	20 g of gypsum, 15 g of ginger, and 9 g of fructus jujubae. The ingredients were decocted with water, and the decoction was taken twice a day. One dose was taken every other day.			
3	PZhang	2022	DQLT: 44<br>Control: 45	DQLT:24/20<br>Control:21/24	DQLT:(8.40±1.17)<br>Control:(8.37±1.12)	NR (NR)<br>TCM syndrome differentiation: Exterior cold with interior heat	Nebulized therapy was administered using budesonide suspension mixed with saline solution at 2 mL per dose, twice daily.	DQLT comprised 6–10 g of honey-processed Ephedra (with stems removed), 15 g of gypsum, 10 g each of cassia twig (peeled), honey-roasted licorice root and apricot kernel, 8 g of ginger, and 10 fructus jujubae. Additionally, 10 g each of descurainia Semen and perilla seed were added according to the disease condition to address the	28	F23, F21	F11,

								phlegm-turbidity syndrome. For patients with blood stasis, 5 g each of *Salvia miltiorrhiza* root, peach kernel, and red peony root was added. For patients with healthy qi deficiency, 5 g each of *Astragalus membranaceus* root, *Angelica sinensis* root, bighead atractylodes rhizome, and *Angelica dahurica* root were added. The ingredients were decocted with water twice to obtain a 300 mL decoction. The decoction was taken 3 times a day, with 100 mL each time.		
4	LJin	2021	LJZTHYPFS: 50<br>Control: 50	LJZTHYPFS: 26/24<br>Control: 27/23	LJZTHYPFS: 6.73±1.65<br>Control: 6.43±1.15	Chronic persistent stage (NR)<br>TCM syndrome differentiation: Lung and Spleen Qi Deficiency	Montelukast sodium was administered as a single daily dose at the following doses: patients aged < 6	Basic treatments, including asthma-relieving, oxygen inhalation, and	90	F3, F4, F5, F19

years: 4 mg per dose; patients aged 6–10 years: 5 mg per dose; patients aged > 10 years: 10 mg per dose, once daily.

anti-infective therapies, were administered, followed by LJZTHYPFS combined with montelukast sodium. The administration method and dosage of montelukast sodium were identical to those of the control group. LJZTHYPFS was prepared by decocting 9 g each of citrus reticulata peel, divaricate saposhnikovia root, pinellia ternata, radix astragali, atractylodes rhizome, poria cocos, and radix codonopsis, and 6 g of licorice root with water. One dose was prepared per day, and the warm decoction was taken in the morning and evening.

5	LXiao	2017	LJZTHYPFS: 100<br>Control: 100	LJZTHYPFS: 62/38<br>Control: 57/43	LJZTHYPFS: 8.5±1.4<br>Control: None	NR (NR)<br>TCM syndrome differentiation: Lung and Spleen Qi Deficiency	Salmeterol/fluticasone powder for inhalation was administered at 1 puff per dose, twice daily.	LJZTHYPFS comprised 9 g of radix astragali, 6 g of radix codonopsis, 6 g of atractylodes rhizome, 6 g of poria cocos, 3 g of citrus reticulata peel, 3 g of pinellia ternata, 3 g of divaricate saposhnikovia root, and 3 g of licorice root. Medicated leaven was added for patients with poor appetite. Fructus corni was added for patients with excessive perspiration. The ingredients were decocted with water to prepare 1 dose per day and the warm decoction was taken twice daily, in the morning and evening, respectively.	180	F41, F19, F26, F27
6	YZLIU	2019	LJZTHYPFS: 49<br>Control: 48	LJZTHYPFS: 24/25<br>Control: 28/19	LJZTHYPFS: 8.20±0.26<br>Control: 7.60±0.29	Chronic persistent stage (NR)<br>TCM syndrome differentiation: Lung and Spleen Qi Deficiency	Budesonide for inhalation was administered at 200 μg per dose once daily. Patients were asked to	In addition to the control treatment, LJZTHYPFS was administered, which comprised 9 g each of	90	F3, F4, F5, F11, F16, F28, F29

							rinse the mouth after each inhalation. The treatment duration was 6 months.	radix codonopsis, radix astragali, poria cocos, atractylodes rhizome, pinellia ternata, citrus reticulata peel, and divaricate saposhnikovia root, and 6 g of licorice root. The ingredients were decocted with water to prepare 1 dose per day, and the warm decoction was taken twice daily, in the morning and evening, respectively.		
7	RLi	2010	LJZTHYPFS: 30<br>Control: 31	LJZTHYPFS: 19/11<br>Control: 18/13	LJZTHYPFS: (22.43±10.59) months<br>Control: (21.87±9.72) months	Clinical remission stage (NR)<br>TCM syndrome differentiation: Lung and Spleen Qi Deficiency	Budesonide aerosol (metered-dose inhaler) was administered for inhalation at a dose of 100 μg twice daily (morning and evening)	In addition to budesonide inhalation, LJZTHYPFS was administered in the remission phase, which comprised 9–12 g of radix astragali, 6–9 g of radix codonopsis, 6–9 g of atractylodes rhizome, 6–9 g of poria cocos, 3–6 g of citrus reticulata	30–60	F1, F2, F41, F42, F43, F17, F18, F39, F40, F27

peel, 3–6 g of pinellia ternata, 3–6 g of divaricate saposhnikovia root, and 3 g of licorice root. Medicated leaven, fructus hordei germinatus, and scorched hawthorn fruit were added for patients with poor appetite. Fructus corni, dragon bones, and concha ostreae were added for patients with malacosarcosis and excessive perspiration. The ingredients were decocted with water to prepare 1 dose per day, and the warm decoction was taken twice daily, in the morning and evening, respectively. One course of treatment lasted for 10 days. Patients received 1 to 2

								courses within one month, with a total of 3 to 6 courses administered. During asthma attacks, both groups additionally received terbutaline inhalation (Bricasol inhaler, 1 spray each time, 2–4 times daily). Patients with obvious infections were also given anti-infective therapies, usually oral or intravenous administration of ribavirin at 10 mg/(kg·d). Patients with signs of bacterial infection additionally received antibiotics. Patients with serious symptoms were required to be hospitalized.		
8	MLiu	2021	LJZT: 60<br>Control: 60	60/60	9.5±1.6/(8.5±1.3	NR (NR)<br>TCM syndrome differentiation:	Montelukast sodium tablets were administered	In addition to the basic treatment with	90	F3, F4, F11, F19, F21,

						Lung and Spleen Qi Deficiency	once daily at 1 tablet (4 mg) per dose.	montelukast sodium tablets, LJZT was administered, which comprised 9 g of ginseng, 9 g of poria cocos, 9 g of atractylodes rhizome, 9 g of pinellia ternata, 9 g of citrus reticulata peel, and 6 g of licorice root. The ingredients were decocted with water to prepare 1 dose per day, and the decoction was taken twice daily, in the morning and evening, respectively.		F31, F32
9	HFang	2022	LJZT: 40<br>Control: 40	LJZT: 24/16<br>Control: 17/23	LJZT: 8.76±1.71<br>Control: 8.37±1.27	NR (NR)<br>TCM syndrome differentiation: Lung and Spleen Qi Deficiency	Oral treatment with montelukast sodium tablets was administered at 4 mg per dose once daily.	In addition to the control treatment, LJZT was administered, which comprised 9 g each of atractylodes rhizome, ginseng, processed pinellia ternata, poria cocos, and citrus reticulata peel, and 6 g of	90	F3, F4, F11, F19, F21, F22, F32

								licorice root. The above ingredients were mixed with 400 mL of water and decocted to 300 mL as a dose, which was taken separately in the morning and evening.		
10*	GJSha	2025	LJZT: 30<br>Control: 30	LJZT: 14/16<br>Control: 13/17	LJZT: 8.75±1.72<br>Control: 8.36±1.26	Clinical remission stage (NR)<br>TCM syndrome differentiation: Lung and Spleen Qi Deficiency	Montelukast sodium was administered orally at 4 mg per dose once daily.	In addition to the control treatment, LJZT was administered, which comprised 9 g each of citrus reticulata peel, processed pinellia ternata, atractylodes rhizome, poria cocos, and radix pseudostellariae, and 6 g of licorice root. The above ingredients were mixed with 400 mL of warm water and decocted to obtain a 300 mL decoction as a dose, which was taken separately in the morning and evening.	30	F4, F23, F11, F12, F17, F18, F39, F19

11	HHLiu	2021	LJZTHYPFS: 90<br>Control: 92	LJZTHYPFS: 53/37<br>Control: 52/40	LJZTHYPFS: 7.70±2.02<br>Control: 7.72±2.04	Chronic persistent stage (NR)<br>TCM syndrome differentiation: Lung and Spleen Qi Deficiency	Budesonide aerosol was administered at 2 puffs per dose once daily.	In addition to control treatment, LJZTHYPFS was administered, which comprised 15 g of radix astragali, 10 g of divaricate saposhnikovia root, 10 g of radix codonopsis, 10 g of poria cocos, 10 g of atractylodes rhizome, 10 g of citrus reticulata peel, 10 g of pinellia ternata, and 5 g of licorice root. For children with excessive phlegm, 10 g each of bulbus fritillariae thunbergii and semen coicis, and 6 g of semen raphanin were added. For children with a deficiency of yin, 10 g each of medicated leaven and endothelium corneum gigeriae galli were added. The	180	F3, F4, F5, F6, F11, F16, F19

								ingredients were decocted with water. One dose of the warm decoction was taken separately in the morning and evening each day for 6 months.		
12	HYWang	2014	MXSGT: 30<br>Control: 30	MXSGT: 19/11<br>Control: 17/13	MXSGT: 7.5±1.9<br>Control: 8.1±2.3	NR (mild 13.3%; moderate 61.7%, severe 25.0%)<br>TCM syndrome differentiation: Heat-type asthma	Patients were given nebulized therapy with budesonide, oral treatment with ketotifen, and treatment with bronchodilators such as bronchial spasmolytics, β2-agonists, and theophyllines (discontinued after symptoms and signs resolved).	The basic formula comprised 6 g of ephedra, 9 g of apricot kernel, 24 g of gypsum, and 6 g of licorice root. Treatment was based on pattern differentiation. For patients with severe fever, coughing, and expectoration, 5 g each of Flos lonicerae, Fructus forsythiae, and scutellaria baicalensis root were added. For patients with yellow and thick phlegm, 10 g of bulbus fritillariae cirrhosae and 5 g each of pericarpium	14	F3, F4, F12, F19

trichosanthis, semen lepidii, and fructus perillae were added. For patients with severe sore throat, 10 g of Belamcanda Rhizoma was added. For patients with an evident history of allergy, 5 g each of white mustard seed, flos carthami, pheretima, and roasted fructus perillae were added. The ingredients were decocted with water to obtain a 200–250 mL decoction as a dose in one day, which was taken in three divided doses in the morning, at noon, and in the evening. Three doses were taken per week. One course of treatment lasted 7 days. After 2 courses, the efficacy was assessed.

13	YQSong	2016	MXSGTHSTW: 50<br>Control: 50	MXSGTHSTW: 24/26<br>Control: 22/28	MXSGTHSTW: 6.5±1.1<br>Control: 6.8±1.0	Acute attack stage (mild 17.0%, moderate 69.0%, severe 14.0%)<br>TCM syndrome differentiation: Heat-type asthma	Oxygen inhalation therapy, phlegm-resolving therapy, antiasthmatic therapy, and fluid and electrolyte replacement therapy were given, along with appropriate antibiotic intervention in the presence of infection in the patient.	The prescription comprised 18 g of gypsum, 10 g of apricot kernel, 6 g of ephedra, 5 g of licorice root, 3–6 g of semen lepidii, and 3–6 g of fructus perillae. One dose was prepared and taken in three divided doses within a day. The patients were monitored for vital signs and drug adverse reactions during the treatment.	3–5	F3, F5, F19, F20, F30
14	CYuan	2021	QQHTT: 100<br>Control: 100	QQHTT: 54/46<br>Control: 59/41	QQHTT: 5.21±1.18<br>Control: 5.37±1.13	NR (non-critical)<br>TCM syndrome differentiation: Heat-type asthma	Montelukast sodium chewable tablets were orally administered at 4 mg per dose for children aged < 5 years and 5 mg per dose for children aged ≥ 5 years once daily.	QQHTT was administered orally, which comprised 10 g of poria cocos, 10 g of Scutellariae Radix, 10 g of apricot kernel, 9 g of whole trichosanthes fruit, 6 g of arisaema cum bile, 6 g of citrus reticulata peel, 6 g of bulbus fritillariae thunbergii, 6 g of	7	F3, F4, F6, F19, F24

								alum-processed pinellia ternata tuber, 3 g of scorched hawthorn fruit, 3 g of Areca catechu, 3 g of Fructus Aurantii Immaturus, and 3 g of licorice root. The above ingredients were added with water and decocted. The dregs were filtered out to obtain 200 mL of decoction. One dose of the warm decoction was taken separately in the morning and evening. The children received the decoctions continuously for 1 week and then came back to the hospital for re-examination, and the efficacy was assessed then.		
15	LZhou	2021	RSWWZT: 24<br>Control: 22	RSWWZT: 15/9<br>Control: 13/9	RSWWZT: 11.2±5.4<br>Control: 10.4±4.5	NR (NR)<br>TCM syndrome differentiation: Spleen and Lung Qi Deficiency	All patients received inhaled corticosteroids (ICS) via nebulization. If	The treatment group was additionally administered with	90	F3, F4, F19, F25, F36, F37

							wheezing worsened, bronchodilator was administered via nebulization, supplemented with cough-suppressing and phlegm-resolving therapies. Antimicrobial agents were added in the presence of bacterial infection.	RSWWZT comprising 4–8 g of ginseng, 3–6 g of atractylodes rhizome, 5–10 g of poria cocos, 3 g of licorice root, 5–10 g of radix ophiopogonis, 3 g of schisandra, 3–6 g of pinellia ternata, and 3–6 g of citrus reticulata peel. The ingredients were decocted with water to obtain approximately 10 mL of decoction as a dose each day. The warm decoction was taken separately in the morning and evening.		
16	HLi	2021	RSWWZT: 51<br>Control: 51	RSWWZT: 24/27<br>Control: 25/26	RSWWZT: 5.25±1.08<br>Control: 5.49±1.15	NR (NR)<br>TCM syndrome differentiation: Lung and Spleen Qi Deficiency	Budesonide suspension was administered at 0.5–1 mg per dose twice daily via nebulization using single-use nebulizers manufactured by Nanchang Aomei Medical Devices Co., Ltd., with	In addition to the control treatment, RSWWZT was administered, which comprised 3 g each of ginseng, Yunnan white poria, and Hangzhou radix ophiopogonis, 4.5 g of rinsed atractylodes	60	F41, F42, F43, F19, F35

							oxygen flow rate set at 5–8 L/min and each nebulization session lasting 10–15 minutes.	rhizome, 1.5 g of Fructus schisandrae chinensis, 2.4 g of honey-fried licorice root, 3 fructus jujubae, and 3 slices of ginger. The ingredients were decocted with water to 200 mL as a dose each day, which was taken twice daily, in the morning and evening, 100 mL each time.		
17*	ZMing	2024	RSWWZT: 40<br>Control: 40	RSWWZT: 21/19<br>Control: 22/18	RSWWZT: 5.69±1.52<br>Control: 5.48±1.54	Clinical remission stage (Control group: 30.0%/35.0%/35.0%; Observation group: 32.5%/35.0%/32.5%)<br>TCM syndrome differentiation: Lung and Spleen Qi Deficiency	Prednisone was administered orally at 30 mg per dose once daily.	The observation group received RSWWZT + control (prednisone). RSWWZT comprised 6 g of honey-fried licorice root, 9 g of ginseng, 10 g of schisandra, 15 g of Massa Medicata Fermentata, 15 g of radix ophiopogonis, 15 g of Hordei Fructus Germinatus, 15 g of Lycii Fructus, and 15 g	90	F3, F4, F13, F9, F25, F46, F45, F20, F30

								of poria cocos. The ingredients were decocted with water to obtain 200 mL of decoction as a dose, which was taken separately in the morning and evening each day. Both groups received the treatment for 3 months.		
18	LNLiang	2022	SGMHT: 38<br>Control: 38	SGMHT: 22/16<br>Control: 23/15	SGMHT: 6.65±0.77<br>Control: 6.64±0.78	Acute attack stage (NR)<br>TCM syndrome differentiation: Cold-type asthma	Montelukast sodium was administered orally every night at bedtime at 4 mg per dose for children aged 3–5 years and 5 mg per dose for children aged 6–14 years.	The observation group additionally received tailored SGMHT, which comprised 9 g each of ephedra, belamcanda, ginger, and pinellia ternata, 6 g each of flos farfarae, fructus perillae, and radix asteris, 3 g each of herba asari and schisandra, and 7 fructus jujubae. For patients with exterior cold and interior fluid retention, SGMHT may be	28	F4, F5, F11, F13, F38

combined with XQLT. For patients with an adverse rise of qi and phlegm surge, bitter apricot kernel, Citri Exocarpium Grandis, semen lepidii, and white mustard seed were added. For patients with coughing, wheezing, and shortness of breath, radix paeoniae alba was added. For patients with itchy throat or dry throat, periostracum cicadae, radix platycodonis, and fresh licorice root were added. For patients with a sore throat, fructus arctii, radix scrophulariae, and herba menthae were added. For patients with symptoms of allergic rhinitis, perilla leaf, flos magnoliae, and

								divaricate saposhnikovia root were added. For patients with irritating coughing, honey-roasted white mulberry root bark, and scutellaria baicalensis root were added. The dosage was determined based on the disease condition and adjusted for body weight. The ingredients were decocted with water to obtain a 100 mL decoction as a dose, which was orally taken twice separately each day. One course of treatment lasted for 28 days.		
19	GFZhang	2024	SGMHT: 52<br>Control: 52	SGMHT: 28/24<br>Control: 31/21	SGMHT: 4.27±1.15<br>Control: 4.45±1.26	NR (NR)<br>TCM syndrome differentiation: Cold-type asthma	Conventional biomedical treatments such as oxygen therapy, fluid and electrolyte replacement therapy, and cough and asthma relieving therapy	The observation group received control treatment plus SGMHT, which comprised 15 g of belamcanda, 15 g of pinellia ternata, 15 g of	14	F3, F4, F5, F6, B10, F15, F8, F19, F30

							were administered. Specifically, budesonide suspension was administered at 0.5 mg per dose via nebulization twice daily. Montelukast sodium tablets were administered at 4 mg per dose for children aged < 5 years and 5 mg per dose for children ≥ 5 years once daily.	radix asteris, 15 g of flos farfarae, 12 g of ginger, 10 g of ephedra, 7 fructus jujubae, 6 g of schisandra, and 3 g of herba asari. The ingredients were decocted with water to obtain 200 mL decoction as a dose, which was taken separately in the morning and evening each day.		
20*	SWLiu	2003	XQLT: 30<br>Control: 30	XQLT: 16/14<br>Control: 17/13	XQLT: 7.55±2.57<br>Control: 7.62±2.59	Acute attack stage (mild 43.3%–46.7%, moderate 53.3%–56.7%, excluding severe cases)<br>TCM syndrome differentiation: Cold-type asthma	Aminophylline was administered at 4 mg per kilogram of body weight once every 8 hours.	XQLT comprised 6 g each of ephedra, dried ginger, roasted licorice root, Platycodonis Radix, and schisandra, 10 g each of peony root and pinellia ternata, and 3 g of herba asari. Children aged 3–7 years took 1 dose daily, and children aged 7–12 years took 1.5 doses daily, both orally in 3–4 divided doses.	5	F14, F19, F21, F23, F24

								One course of treatment consisted of 5 days.		
21	ZHDing	2022	XQLT: 42<br>Control: 42	XQLT: 21/21<br>Control: 21/21	XQLT: (21.2±0.9) months<br>Control: (21.4±2) months	NR (excluding severe and critical cases)<br>TCM syndrome differentiation: Cold-type asthma	Symptomatic treatment was given, including fluid replacement, oxygen inhalation, nebulization, anti-inflammatory therapy, and antiasthmatic therapy with salbutamol.	The prescription comprised 3 g of herba asari, 9 g of pinellia ternata (rinsed), 6 g of licorice root (roasted), 3 g of schisandra, 3 g of dried ginger, 6 g of cassia twig (peeled), 9 g of ephedra (with stem removed), and 9 g of peony root. The ingredients were decocted with water to obtain 1 dose of decoction daily. The warm decoction was taken separately in three divided doses, with 35 mL each time.	5	F3, F12, F19, F35
22	NLiu	2023	XQLT: 44<br>Control: 44	XQLT: 28/16<br>Control: 27/17	XQLT: 4.14±0.42<br>Control: 4.13±0.63	NR (NR)<br>TCM syndrome differentiation: Cold-type asthma	Montelukast sodium chewable tablets were administered orally at bedtime at 5 mg per dose once daily.	The prescription comprised 6 g of roasted licorice root, 3 g of ephedra, 8 g of radix paeoniae alba, 5 g of	21	F23, F41, F43, F25, F44, F9, F12, F34

Salmeterol/fluticasone powder for inhalation was administered at 25 μg per inhalation twice daily.

cassia twig, 5 g of pinellia ternata, 8 g of schisandra, 3 g of herba asari, and 5 g of dried ginger. If the child experienced severe wind-cold, 8 g of divaricate saposhnikovia root was added. If the child had profuse, sticky phlegm, 8 g of radix platycodonis, 5 g of herba houttuyniae, and 5 g of flos lonicerae were added. If the child had yang deficiency with internal cold, 5 g of fructus arctii and 5 g of fructus xanthii were added. If the child was irritable and thirsty, 8 g of gypsum was added. The ingredients were decocted with water to obtain a 100 mL decoction as a dose,

									which was taken twice separately. One course of treatment lasted for 1 week. Both groups received 3 courses of treatment continuously.		
23	RLU	2017	XQLT: 35<br>Control: 34	XQLT: 21/14<br>Control: 22/12	XQLT: 9.16±2.85<br>Control: 9.47±2.71	Acute attack stage (mild: approximately 58%; moderate: approximately 42%; severe: excluded)<br>TCM syndrome differentiation: Cold-type asthma	Administer Seretide, inhalation twice daily.	1	The observation group was additionally administered with XQLT, which comprised 5 g of roasted ephedra, 8 g of cassia twig, 10 g each of fried radix paeoniae alba, ginger-processed pinellia ternata, and schisandra, 6 g each of rinsed dried ginger and roasted licorice root, and 2 g of herba asari. The granules were soaked in an appropriate amount of warm water to make the decoction. The decoction was taken twice daily. One course of treatment	7	F3, F23, F41, F42, F5, F7, F17, F18, F19, F33, F39

								lasted for a week.		
24	XBDong	2021	YPFSHLJZT: 44<br>Control: 44	YPFSHLJZT: 28/16<br>Control: 26/18	YPFSHLJZT: 6.57±1.10<br>Control: 6.48±1.02	Clinical remission stage (NR)<br>TCM syndrome differentiation: Lung and Spleen Qi Deficiency	Montelukast sodium tablets were administered at 4 mg per dose for children aged 3–6 years and 5 mg for children aged 7–12 years once daily. Concurrently, budesonide powder for inhalation was inhaled at 60 μg per dose for children aged 3–6 years and 100 μg per dose for children aged 7–12 years twice daily.	The observation group was additionally administered with YPFSHLJZT, which comprised 9 g each of radix astragali, radix codonopsis, atractylodes rhizome, poria cocos, citrus reticulata peel, pinellia ternata, and divaricate saposhnikovia root, and 6 g of licorice root. The ingredients were decocted with water to obtain a 300 mL decoction, which was taken separately in the morning and evening. Each patient in both groups took 1 dose daily for 3 months.	90	F9, F11, F14, F19, F23

Notes: DQLT: Da Qing Long Tang; LJZTHYPFS: Liu Jun Zi Tang He Yu Ping Feng San (or YPFSHLJZT, Yu Ping Feng San He Liu Jun Zi Tang); LJZT: Liu Jun Zi Tang; MXSGT: Ma Xing Shi Gan Tang; MXSGTHSTW: Ma Xing Shi Gan Tang He Su Ting Wan; RSWWZT: Ren Shen Wu Wei Zi Tang; SGMHT: She Gan Ma Huang Tang (or tailored version); XQLT: Xiao Qing Long Tang; QQHTT: Qing Qi Hua Tan Tang; F1: frequency; F2: severity; F3: forced expiratory volume in 1s (FEV1); F4: peak expiratory flow (PEF); F5: forced vital capacity (FVC); F6: FEV1/FVC; F7: maximal voluntary ventilation (MVV); F8: C-reactive protein (CRP);

F9: tumor necrosis factor alpha (TNF-α); F10: cytokines; F11: TCM syndrome points; F12: FEV1%; F13: FEV1%/FVC; F14: eosinophil (EOS) count in peripheral blood; F15: serum procalcitonin (PCT); F16: Childhood Asthma Control Test (C-ACT) score; F17: $CD4^+$; F18: $CD8^+$; F19: clinical efficacy; F20: pre-treatment symptom score; F21: interleukin 4 (IL-4); F22: tumor necrosis factor gamma (TNF-γ); F23: immunoglobulin E (IgE); F24: time for improvement of clinical symptoms (mean disease course); F25: interleukin 8 (IL-8); F26: urinary leukotrienes; F27: percentage of blood eosinophils (EOS%); F28: forced expiratory flow 50 (FEF50); F29: forced expiratory flow 75 (FEF75); F30: adverse reactions; F31: interferon gamma (IFN-γ); F32: interleukin 10 (IL-10) anti-inflammatory factor; F33: safety comparison; F34: percentage of peak expiratory flow (PEF%); F35: comparative analysis of improvement of clinical symptoms; F36: interleukin 17 (IL-17); F37: leptin; F38: respiratory function (p[O_2]; p[CO_2]; respiratory rate); F39: $CD4^+/CD8^+$ value based assessment model for efficacy in asthma; F40: $CD3^+$; F41: immunoglobulin G (IgG); F42: immunoglobulin A (IgA); F43: immunoglobulin M (IgM); F44: interleukin 6 (IL-6); F45: neutrophil to lymphocyte ratio (NLR); F46: lipoprotein-associated phospholipase A2 (Lp-PLA2). * represents changes in the monarch/minister herb in the decoction.

Supplementary Table S7 Comparison of effect sizes

Treatment	FEV1 MD (95% CrI)			PEF MD (95% CrI)			FVC MD (95% CrI)			IgE MD (95% CrI)			IgG MD (95% CrI)		
	Main analysis	Sensitivity analysis 1	Sensitivity analysis 2	Main analysis	Sensitivity analysis 1	Sensitivity analysis 2	Main analysis	Sensitivity analysis 1	Sensitivity analysis 2	Main analysis	Sensitivity analysis 1	Sensitivity analysis 2	Main analysis	Sensitivity analysis 1	Sensitivity analysis 2
DQLT	MD 0.89; 95% CrI [0.70, 1.10]	MD 0.89; 95% CrI [0.70, 1.10]	MD 0.89; 95% CrI [0.70, 1.1]	N/A	N/A	N/A	N/A	N/A	N/A	N/A	MD -1.0e+02; 95% CrI [-1.3e+02, -75.0]	N/A	N/A	N/A	N/A
LJZT	MD 0.16; 95% CrI [0.11, 0.21]	MD 0.15; 95% CrI [0.094, 0.20]	MD 0.16; 95% CrI [0.11, 0.21]	N/A	MD 0.86; 95% CrI [0.70, 1.0]	N/A	N/A	N/A	N/A	N/A	N/A	N/A	N/A	N/A	N/A
LJZTHYPFS	MD 0.43; 95% CrI [0.40, 0.47]	MD 0.41; 95% CrI [0.37, 0.45]	MD 0.44; 95% CrI [0.40, 0.47]	N/A	MD 0.88; 95% CrI [0.82, 0.94]	N/A	N/A	MD 0.31; 95% CrI [0.28, 0.34]	N/A	N/A	MD -83.0; 95% CrI [-1.1e+02, -57.0]	N/A	N/A	N/A	N/A
MXSGT	MD 0.41; 95% CrI [0.19, 0.63]	N/A	MD 0.41; 95% CrI [0.19, 0.63]	MD 12.00; 95% CrI [8.00, 17.00]	N/A	N/A	N/A	N/A	N/A	N/A	N/A	N/A	N/A	N/A	N/A
MXSGTHSTW	MD 0.87; 95% CrI [0.69, 1.10]	MD 0.87; 95% CrI [0.69, 1.1]	MD 0.87; 95% CrI [0.69, 1.1]	N/A	N/A	N/A	MD 0.72; 95% CrI [0.10, 1.30]	MD 0.72; 95% CrI [0.45, 0.99]	MD 0.72; 95% CrI [0.10, 0.3]	N/A	N/A	N/A	N/A	N/A	N/A
QQHTT	MD 1.20; 95% CrI [1.10, 1.40]	N/A	MD 1.2; 95% CrI [1.1, 1.4]	N/A	N/A	N/A	N/A	N/A	N/A	N/A	N/A	N/A	N/A	N/A	N/A
RSWWZT	N/A	N/A	MD 0.14; 95% CrI [0.030, 0.25]	N/A	N/A	N/A	N/A	N/A	N/A	N/A	N/A	N/A	N/A	MD 1.6; 95% CrI [1.2, 2.0]	N/A

SGMHT	MD 0.26; 95% CrI [0.088, 0.43]	MD 0.26; 95% CrI [0.088, 0.43]	MD 0.26; 95% CrI [0.088, 0.43]	N/A	MD 0.67; 95% CrI [0.46, 0.88]	N/A	MD 0.48; 95% CrI [0.041, 0.92]	MD 0.42; 95% CrI [0.17, 0.67]	MD 0.48; 95% CrI [0.41, 0.92]	N/A	N/A	N/A	N/A	N/A	N/A
XQLT	MD 0.13; 95% CrI [0.015, 0.24]	MD 0.13; 95% CrI [0.015, 0.24]	MD 0.13; 95% CrI [0.015, 0.24]	N/A	N/A	N/A	N/A	N/A	N/A	N/A	MD -32.0; 95% CrI [-43.0, -22.0]	N/A	MD 3.75; 95% CrI [0.25, 7.74]	MD 5.4; 95% CrI [3.6, 7.1]	MD 3.7; 95% CrI [0.25, 7.7]

Supplementary Table S8 Comparison of SUCRA rankings

Treatment	FEV1 SUCRA/% (rank)			PEF SUCRA/% (rank)			FVC SUCRA/% (rank)			IgE SUCRA/% (rank)			IgG SUCRA/% (rank)		
	Main analysis	Sensitivity analysis 1	Sensitivity analysis 2	Main analysis	Sensitivity analysis 1	Sensitivity analysis 2	Main analysis	Sensitivity analysis 1	Sensitivity analysis 2	Main analysis	Sensitivity analysis 1	Sensitivity analysis 2	Main analysis	Sensitivity analysis 1	Sensitivity analysis 2
Control	0.2(9)	0.3(7)	0.2(9)	6.2(7)	0.0(4)	5.61(7)	4.9(5)	0.02(4)	4.9(5)	13.2(4)	0.0(4)	17.0(5)	13.9(4)	0.0(3)	13.9(4)
DQLT	82.1(2)	92.6(1)	84.0(2)	NR	NR	NR	NR	NR	NR	63.1(2)	94.6(1)	52.7(3)	NR	NR	NR
LJZT	23.0(7)	28.3(5)	27.5(6)	44.1(4)	76.2(2)	47.3(4)	37.3(4)	NR	37.3(4)	NR	NR	64.9(2)	NR	NR	NR
LJZTHYPFS	57.0(4)	65.9(3)	61.8(4)	26.4(6)	86.7(1)	24.5(6)	51.4(3)	39.9(3)	51.4(3)	78.8(1)	72.1(2)	65.3(1)	40.1(3)	NR	40.1(3)
MXSGT	53.0(5)	NR	58.0(5)	97.1(1)	NR	93.5(1)	NR	NR	NR	NR	NR	NR	NR	NR	NR
MXSGTHSTW	80.5(3)	90.7(2)	82.7(3)	NR	NR	NR	89.4(1)	98.1(1)	89.4(1)	NR	NR	NR	NR	NR	NR
QQHTT	99.9(1)	NR	99.9(1)	42.8(5)	NR	40.0(5)	NR	NR	NR	NR	NR	NR	NR	NR	NR
RSWWZT	NR	NR	22.8(7)	80.5(2)	NR	89.7(2)	NR	NR	NR	NR	NR	NR	54.4(2)	50.0(2)	54.4(2)
SGMHT	36.6(6)	47.2(4)	42.2(6)	52.8(3)	37.1(1)	49.3(3)	67.0(2)	62.0(2)	67.0(2)	NR	NR	NR	NR	NR	NR
XQLT	17.8(8)	25.0(6)	20.8(8)	NR	NR	NR	NR	NR	NR	44.9(3)	33.3(3)	50.1(4)	91.6(1)	100.0(1)	91.6(1)

Supplementary Table S9 Comparison of the first-ranked treatment in the SUCRA rankings across the main analysis, sensitivity analysis 1, and sensitivity analysis 2

Outcome	Main analysis (SUCRA, %)	Sensitivity analysis 1 (SUCRA, %)	Sensitivity analysis 2 (SUCRA, %)	Stability
FEV_1	QQHTT (99.9)	DQLT (92.6)	QQHTT (99.9)	Changed
FVC	MXSGTHSTW (89.4)	MXSGTHSTW (98.1)	MXSGTHSTW (89.4)	Stable
PEF	MXSGT (97.1)	LJZTHYPFS (86.7)	MXSGT (93.5)	Changed
IgE	LJZTHYPFS (78.8)	DQLT (94.6)	LJZTHYPFS (65.3)	Changed
IgG	XQLT (91.6)	XQLT (100.0)	XQLT (91.6)	Stable
