# Supplementary material for: Efficacy of traditional Chinese medicine decoctions combined with conventional therapy for pediatric asthma: a network meta-analysis
Source: Front Pharmacol. 2026 Jun 25;17:1780354. doi: 10.3389/fphar.2026.1780354 (PMC13346251; doi:10.3389/fphar.2026.1780354)
Supplement: Supplementary file 2 [file Supplementaryfile2.pdf]

Supplementary Material 2 Annotated R Code, DIC, and Diagnostics

1.Using the FEV_1 R script as an example

R4.4.1 scripts

```
install.packages('gemtc')
install.packages('ggplot2') #Line plot for SUCRA
#Continuous outcome variables

#Load required packages
setwd('D:\\工作目录')

library(gemtc)
library(coda)
#Read data and convert to network format
data <- read.csv('D:\\工作目录\\fev1.csv')
network <- mtc.network(data)
# Draw network plot
plot(network)
#Consistency model fitting
model<-mtc.model(network,type="consistency",n.chain=4,likelihood="normal",link="identity",linearModel="fixed")
results <- mtc.run(model, n.adapt = 20000, n.iter = 50000, thin = 1)
summary(results)
#The output includes DIC; the DIC can be compared with that from the inconsistency model.
# A difference in DIC less than 5 suggests that the consistency assumption holds.
# The absolute DIC value has no interpretation and is only useful for relative comparisons.
# The summary also reports the overall heterogeneity parameter I².
#Inconsistency model
modelume<-mtc.model(network,type="ume",n.chain=4,likelihood="normal",link="identity",linearModel="fixed")
#Run the inconsistency model
resultsme <- mtc.run(model, n.adapt = 20000, n.iter = 50000, thin = 1)
summary(resultsme)
#Forest plot
forest(results)
forest(relative.effect(results, "Placebo"),digits=3,xlim = c(-3,1))
#Use "Placebo" as the reference to show differences from each intervention
plot(results)
# Convergence diagnostics and density plots for pairwise comparisons
# (optional; not required for saving figures)
gelman.plot(results)
# Gelman-Rubin convergence diagnostic plot
# (optional; not required for saving figures)
#Rhat
```

```
library(coda)
# results
mcmc_samples <- as.mcmc.list(results)
gelman.diag(mcmc_samples)
# Treatment ranking
ranks <- rank.probability(results,preferredDirection= 1)
plot(ranks)      #Rankogram (may not display all interventions fully)
plot(ranks,beside = TRUE)          #Bar plot of rank probabilities for each rank
sucra(ranks)                             #Surface under the cumulative ranking curve

write.csv(ranks,"fev1ranks.csv")    #Export rank probabilities
#The exported rank data should be formatted as cumulative probabilities for further plotting


#Generate SUCRA line plot using ggplot2
library(ggplot2)
TFrank<- read.csv('D:/工作目录/fev1ranks.csv') #Read rank probability data
P<-ggplot(TFrank,aes(x=rank,y=prob, colour=treatment)) +geom_line(size=2,lty="solid")
#Cumulative probability curves
p1<-P+geom_point(size=2)+labs(x="rank", y="Cumulative probability")+theme(axis.text = element_text(size=12),axis.title=element_text(size=15),panel.background=element_blank(),axis.line = element_line(color="black"),
legend.title=element_text(size=15,color="black"),legend.position="bottom")
#League table
tb<- relative.effect.table(results) #Generate league table
tb1<-round((tb),2)
write.csv(tb1,"fev1_table.csv") #Export league table
#Local inconsistency assessment using node-splitting (only meaningful when closed loops exist in the network)
resultnodesplit <-mtc.nodesplit(network)
# Parameters can be explicitly specified; the simplified version works because
# gemtc automatically detects the continuous outcome type and applies default settings.
b <- summary(resultnodesplit)
print(b)          # Display node-splitting comparison results
plot(b,digits = 5)
# Heterogeneity test
resultanohe <- mtc.anohe(network)        # Heterogeneity assessment
c<-summary(resultanohe)
print(c)
plot(c,digits = 5)
```

Using StataIC 15 (64-bit) to analyze Stata

```
network setup mean stddev samplesize,studyvar(study) trtvar(treatment) format(augment)
network map,improve    # Draw network plot
```

```
#funnel plot
network convert pairs
replace _t1="Control" if _t1=="A"
replace _t1="DQLT" if _t1=="B"
replace _t1="LJZT" if _t1=="C"
replace _t1="LJZTHYPFS" if _t1=="D"
replace _t1="MXSGT" if _t1=="E"
replace _t1="MXSGT+STW" if _t1=="F"
replace _t1="QQHTT" if _t1=="G"
replace _t1="RSWWZT" if _t1=="H"
replace _t1="SGMHT" if _t1=="I"
replace _t1="XEDCT" if _t1=="J"
replace _t1="XQLT" if _t1=="K"

replace _t2="Control" if _t2=="A"
replace _t2="DQLT" if _t2=="B"
replace _t2="LJZT" if _t2=="C"
replace _t2="LJZTHYPFS" if _t2=="D"
replace _t2="MXSGT" if _t2=="E"
replace _t2="MXSGT+STW" if _t2=="F"
replace _t2="QQHTT" if _t2=="G"
replace _t2="RSWWZT" if _t2=="H"
replace _t2="SGMHT" if _t2=="I"
replace _t2="XEDCT" if _t2=="J"
replace _t2="XQLT" if _t2=="K"
netfunnel _y _stderr _t1 _t2, bycomparison    ytitle(Standard error of logor)    addplot(lfit _stderr _ES_CEN)
```

2.DIC and diagnostics

2.1 FEV1

Main analysis:

Consistency model - Model fit (residual deviance): fixed

Dbar	pD	DIC
34.30393	18.97542	53.27935

22 data points, ratio 1.559, I^2 = 39%

Inconsistency model - Model fit (residual deviance): fixed

Dbar	pD	DIC
34.33780	19.00929	53.34709

22 data points, ratio 1.561, I^2 = 39%

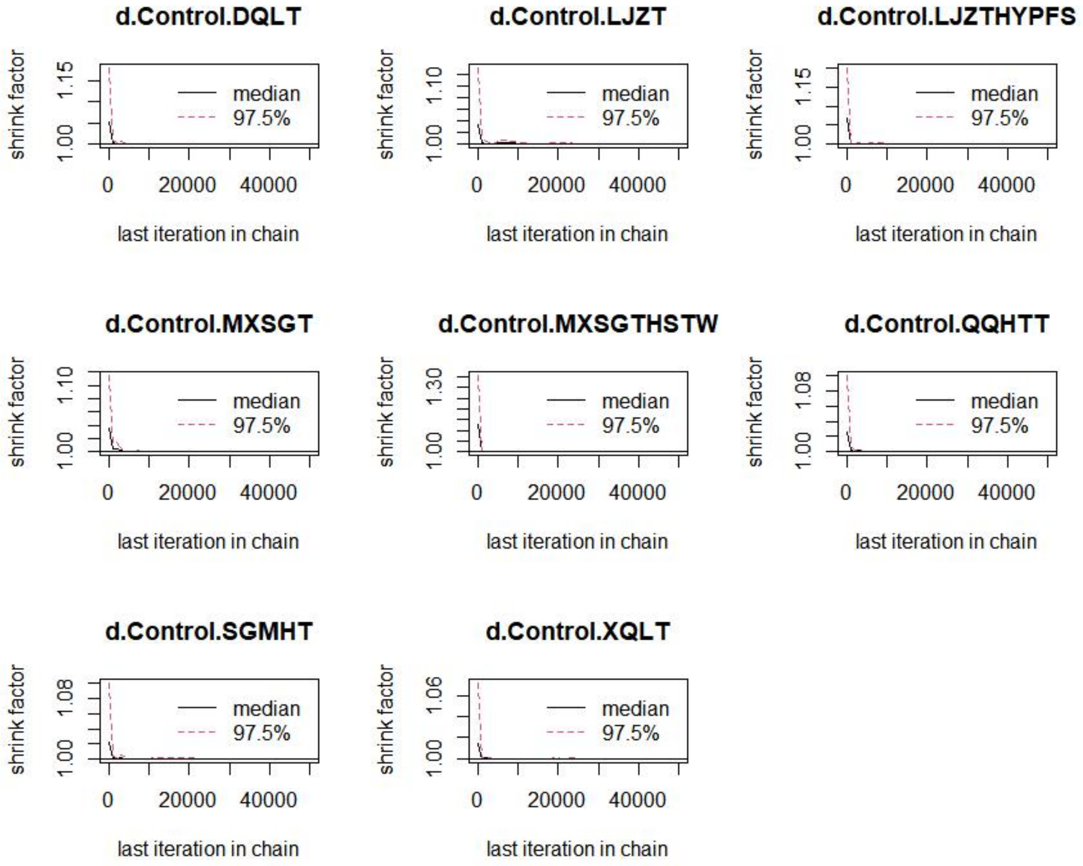
d.Control.DQLT
d.Control.LJZT
d.Control.LJZTHYPFS
d.Control.MXSGT
d.Control.MXSGTHSTW
d.Control.QQHTT
d.Control.SGMHT
d.Control.XQLT
shrink factor
median
97.5%
last iteration in chain

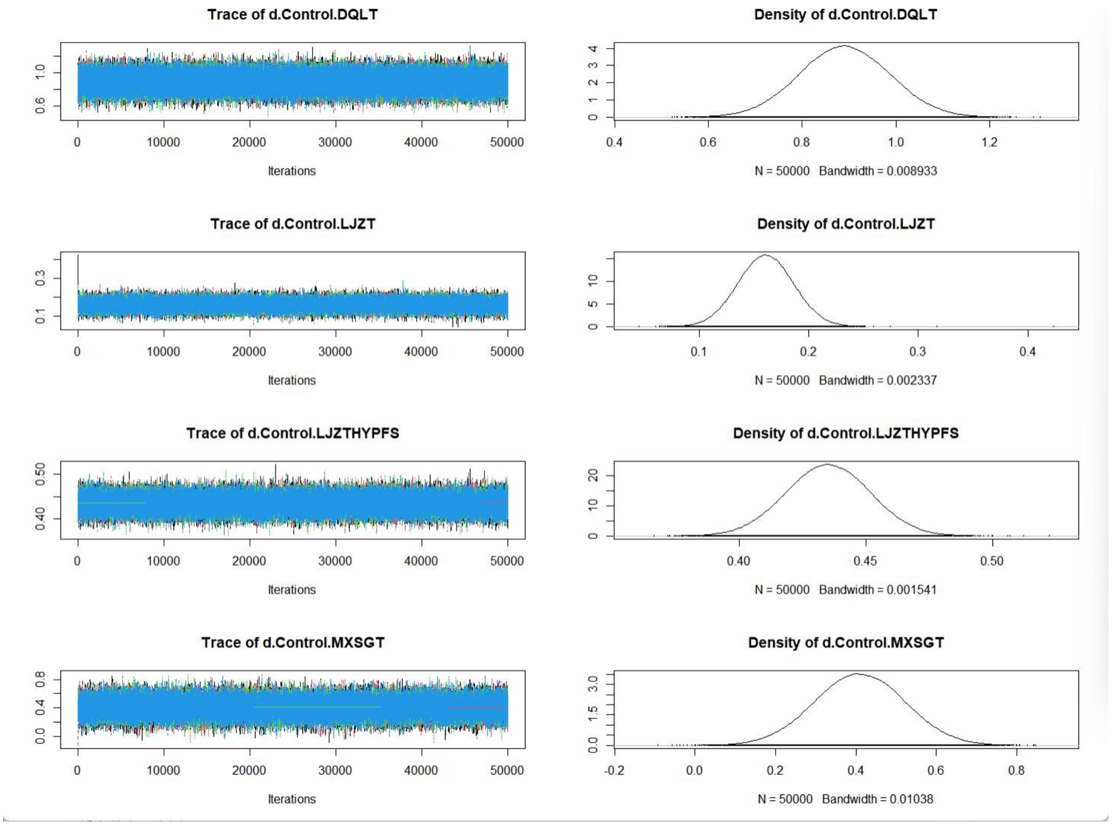
Trace of d.Control.DQLT
Density of d.Control.DQLT
N = 50000 Bandwidth = 0.008933
Trace of d.Control.LJZT
Density of d.Control.LJZT
N = 50000 Bandwidth = 0.002337
Trace of d.Control.LJZTHYPFS
Density of d.Control.LJZTHYPFS
N = 50000 Bandwidth = 0.001541
Trace of d.Control.MXSGT
Density of d.Control.MXSGT
N = 50000 Bandwidth = 0.01038
Iterations

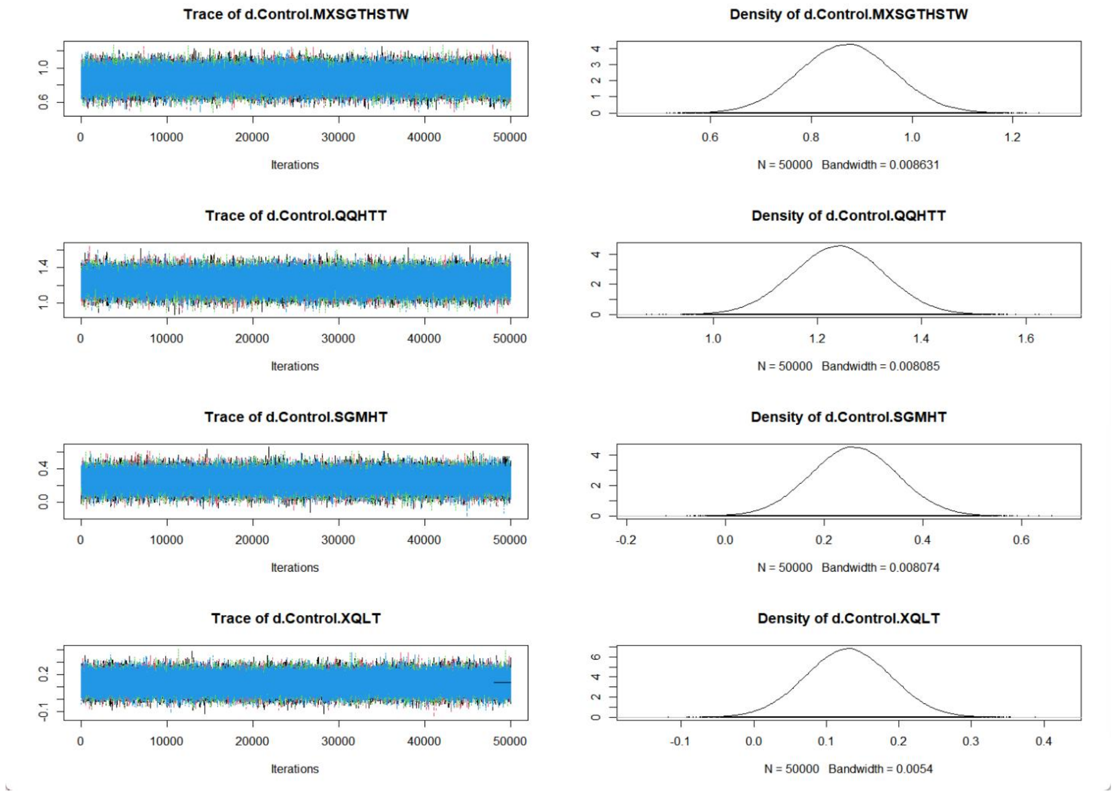

Sensitivity analysis 1:

Consistency model - Model fit (residual deviance): fixed

Dbar	pD	DIC
19.19097	14.01251	33.20348

16 data points, ratio 1.199, I^2 = 22%

Inconsistency model - Model fit (residual deviance): fixed

Dbar	pD	DIC
19.19257	14.01413	33.20670

16 data points, ratio 1.178, I^2 = 22%

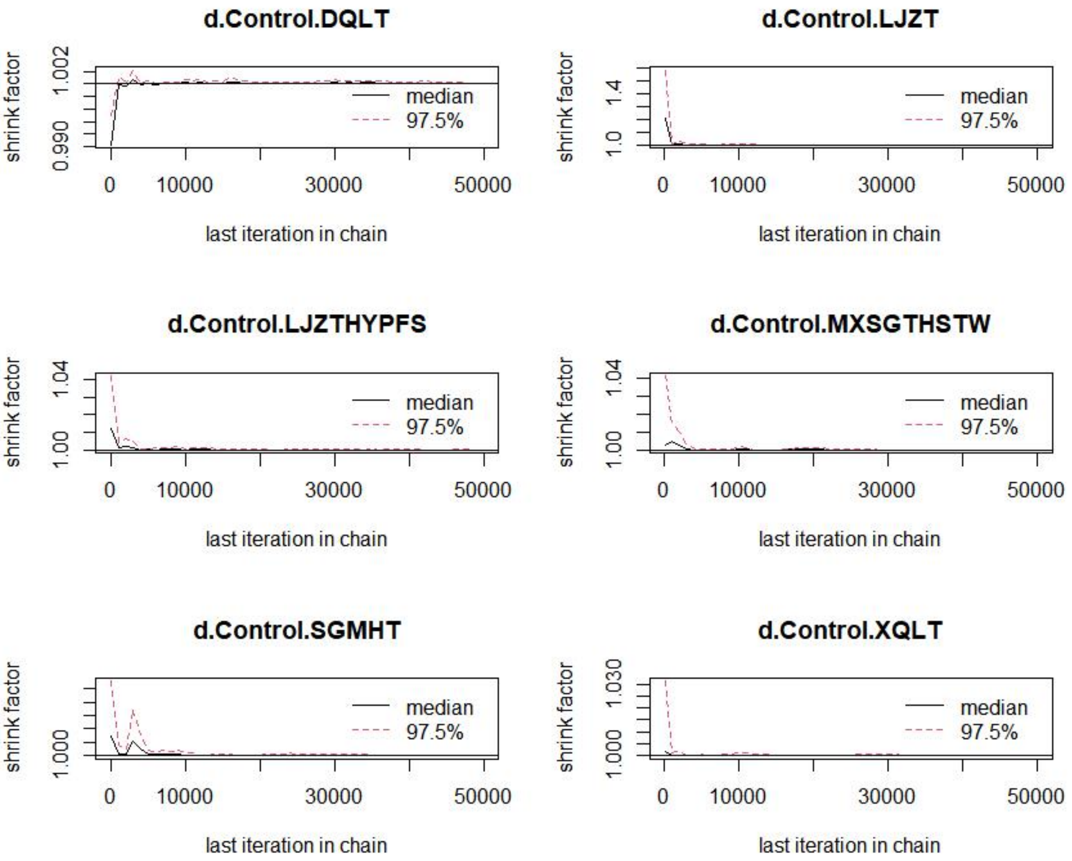
d.Control.DQLT
d.Control.LJZT
d.Control.LJZTHYPFS
d.Control.MXSGTHSTW
d.Control.SGMHT
d.Control.XQLT
shrink factor
last iteration in chain
median
97.5%
0
10000
30000
50000
0.990
1.002
1.0
1.4
1.00
1.04
1.000
1.030

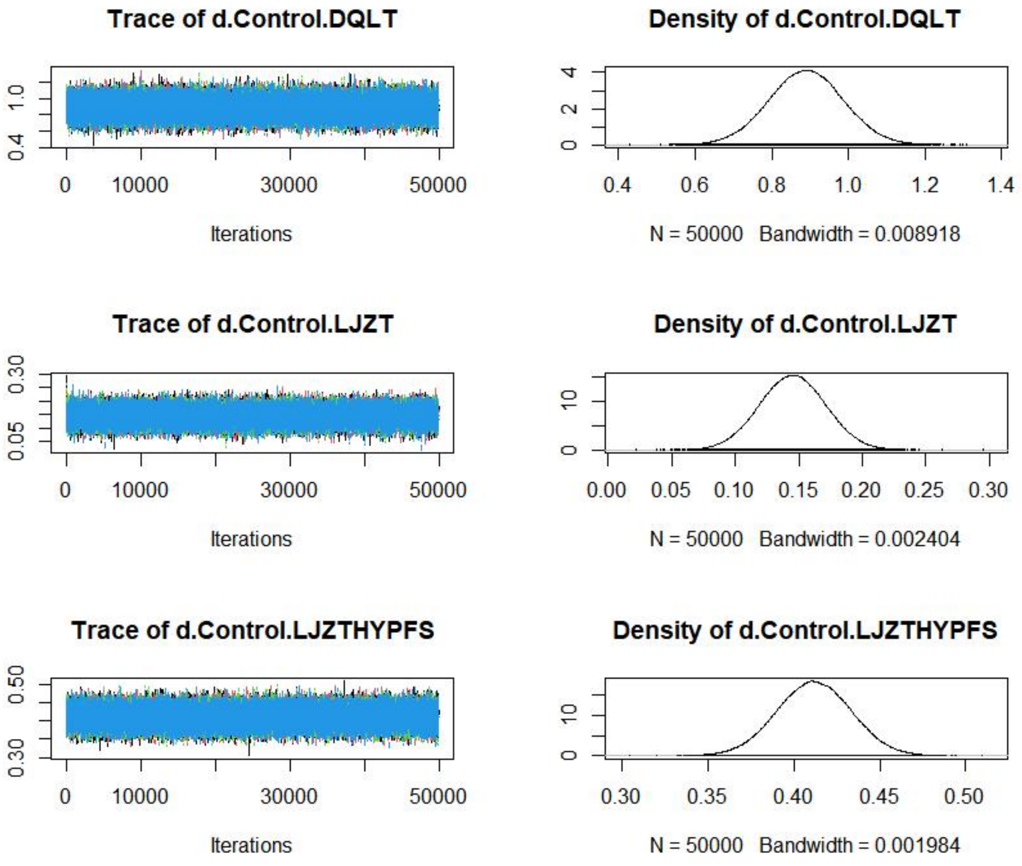
Trace of d.Control.DQLT
Iterations
Density of d.Control.DQLT
N = 50000 Bandwidth = 0.008918
Trace of d.Control.LJZT
Iterations
Density of d.Control.LJZT
N = 50000 Bandwidth = 0.002404
Trace of d.Control.LJZTHYPFS
Iterations
Density of d.Control.LJZTHYPFS
N = 50000 Bandwidth = 0.001984

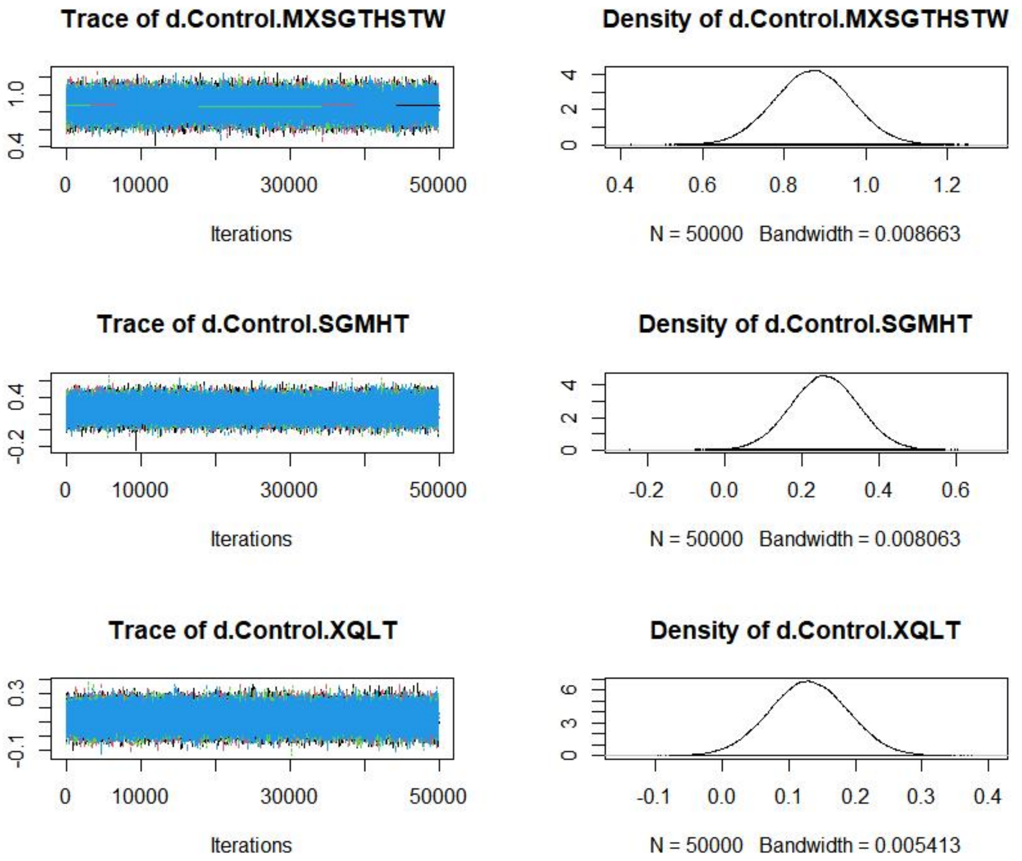

Sensitivity analysis 2:

Consistency model - Model fit (residual deviance): fixed

Dbar	pD	DIC
36.33499	21.00644	57.34143

24 data points, ratio 1.514, I^2 = 37%

Inconsistency model - Model fit (residual deviance): fixed

Dbar	pD	DIC
36.30848	20.97977	57.28825

24 data points, ratio 1.513, I^2 = 37%

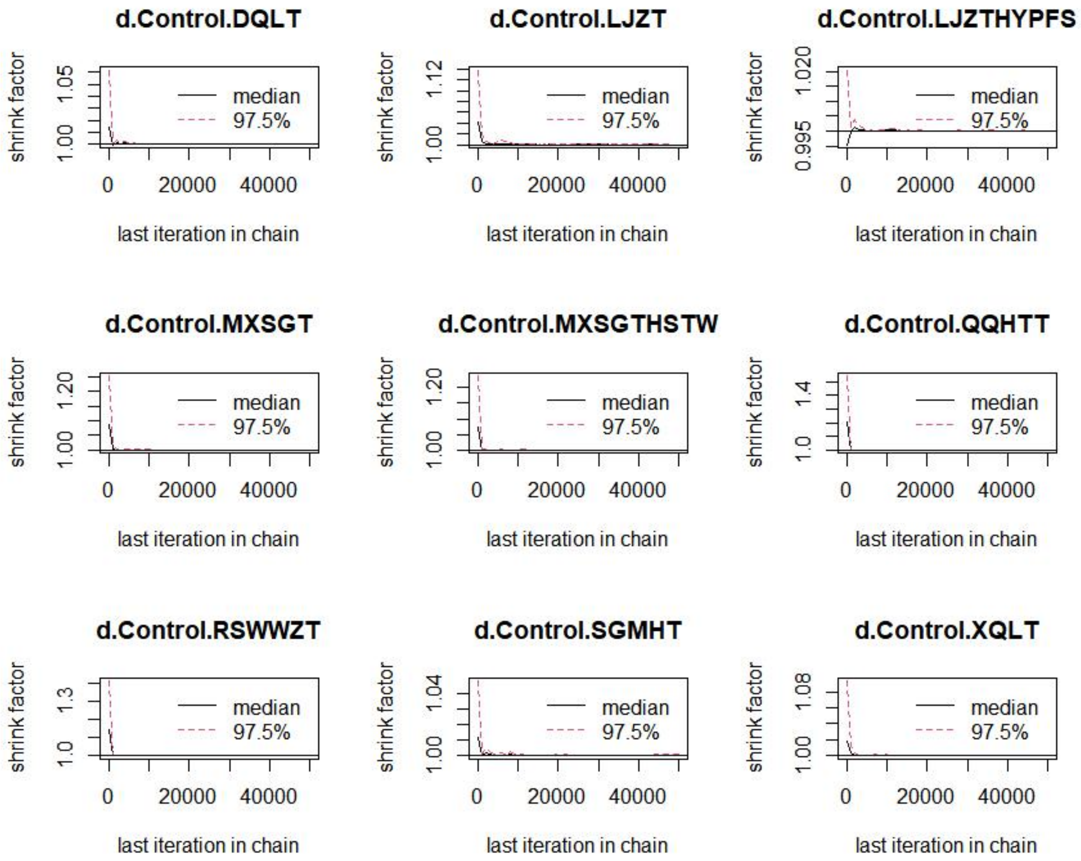
d.Control.DQLT
d.Control.LJZT
d.Control.LJZTHYPFS
d.Control.MXSGT
d.Control.MXSGTHSTW
d.Control.QQHTT
d.Control.RSWWZT
d.Control.SGMHT
d.Control.XQLT
shrink factor
median
97.5%
last iteration in chain
0
20000
40000

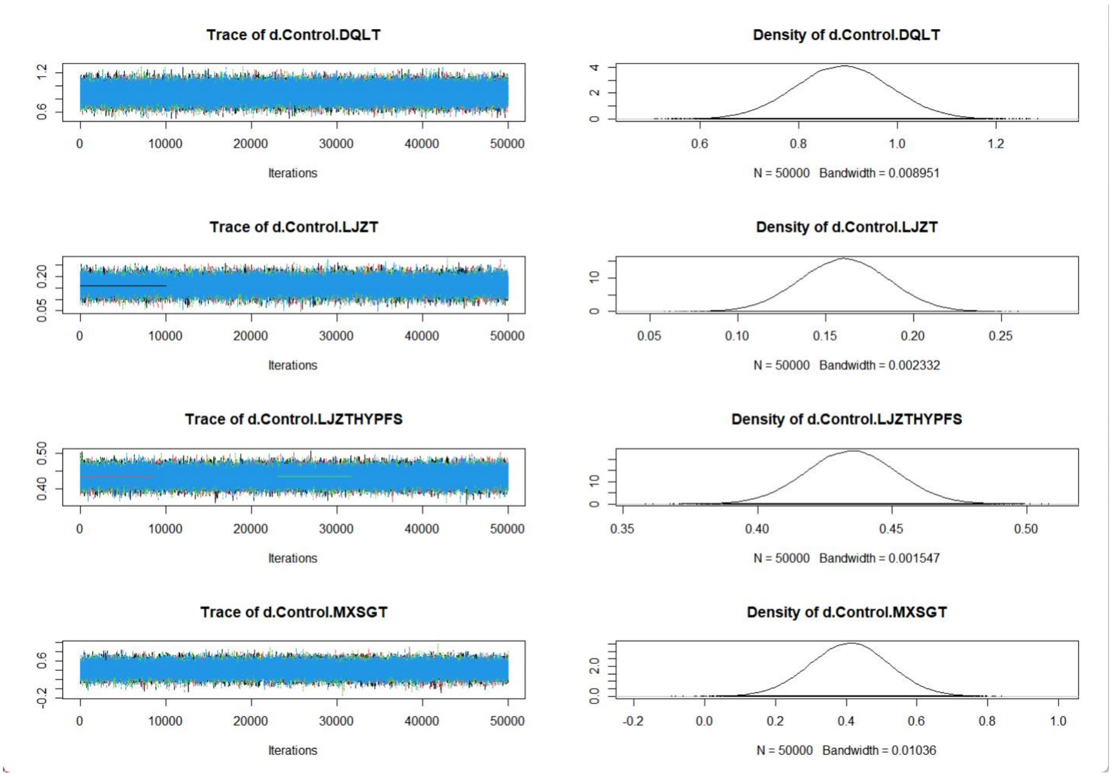
Trace of d.Control.DQLT
Density of d.Control.DQLT
Iterations
N = 50000 Bandwidth = 0.008951
Trace of d.Control.LJZT
Density of d.Control.LJZT
Iterations
N = 50000 Bandwidth = 0.002332
Trace of d.Control.LJZTHYPFS
Density of d.Control.LJZTHYPFS
Iterations
N = 50000 Bandwidth = 0.001547
Trace of d.Control.MXSGT
Density of d.Control.MXSGT
Iterations
N = 50000 Bandwidth = 0.01036

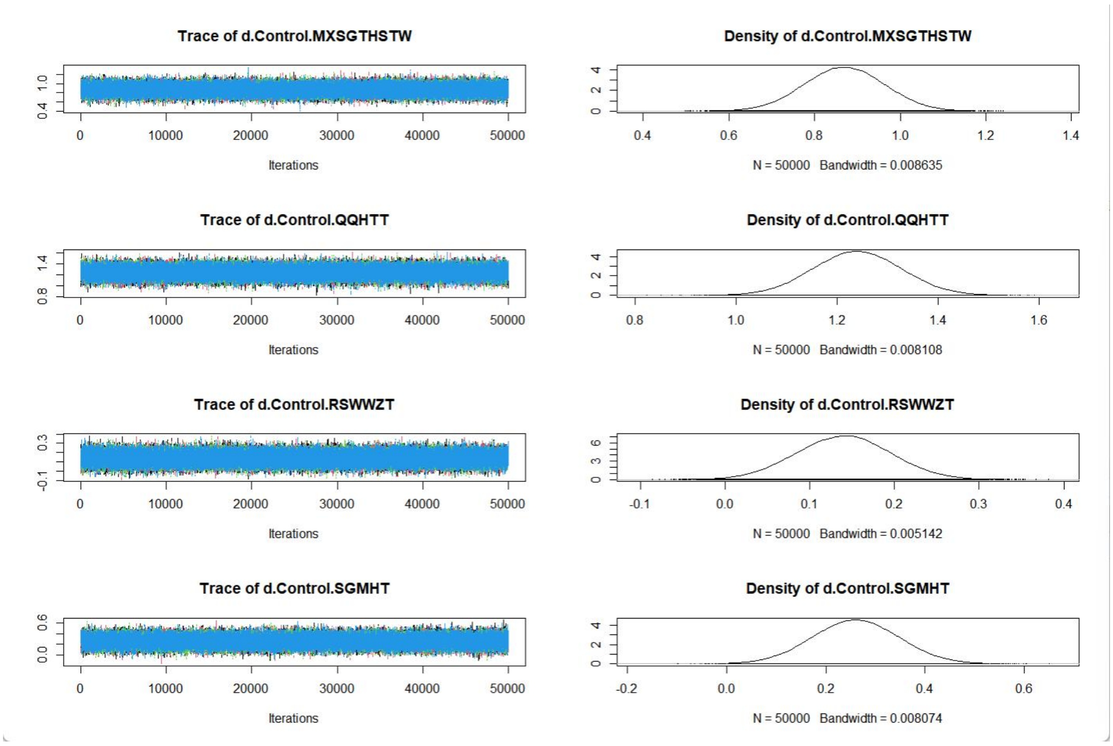

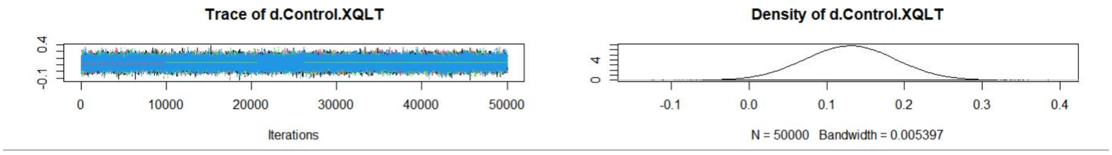

2.2 FVC

Consistency model - Model fit (residual deviance): random

Dbar	pD	DIC
11.73806	11.61402	23.35208

12 data points, ratio 0.9782, I^2 = 6%

Inconsistency model - Model fit (residual deviance): random

Dbar	pD	DIC
11.74094	11.61957	23.36050

12 data points, ratio 0.9784, I^2 = 6%

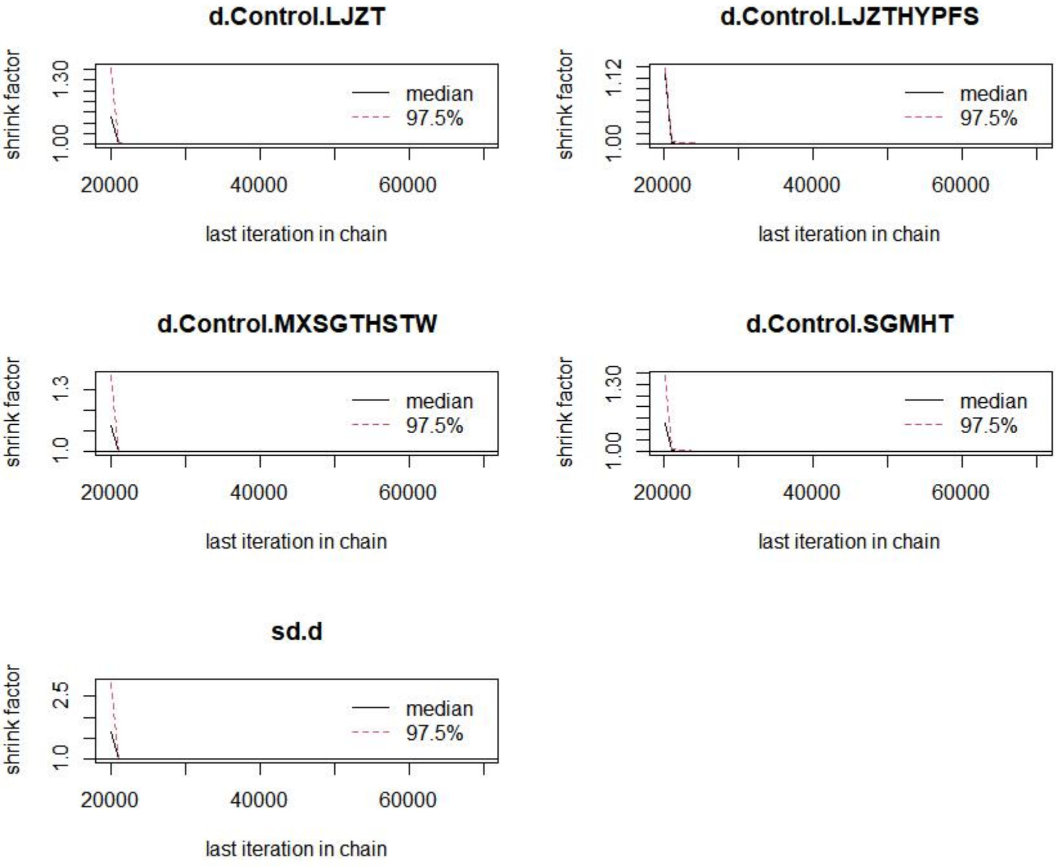
d.Control.LJZT
d.Control.LJZTHYPFS
d.Control.MXSGTHSTW
d.Control.SGMHT
sd.d
shrink factor
last iteration in chain
median
97.5%
20000
40000
60000
1.00
1.30
1.12
1.0
1.3
2.5

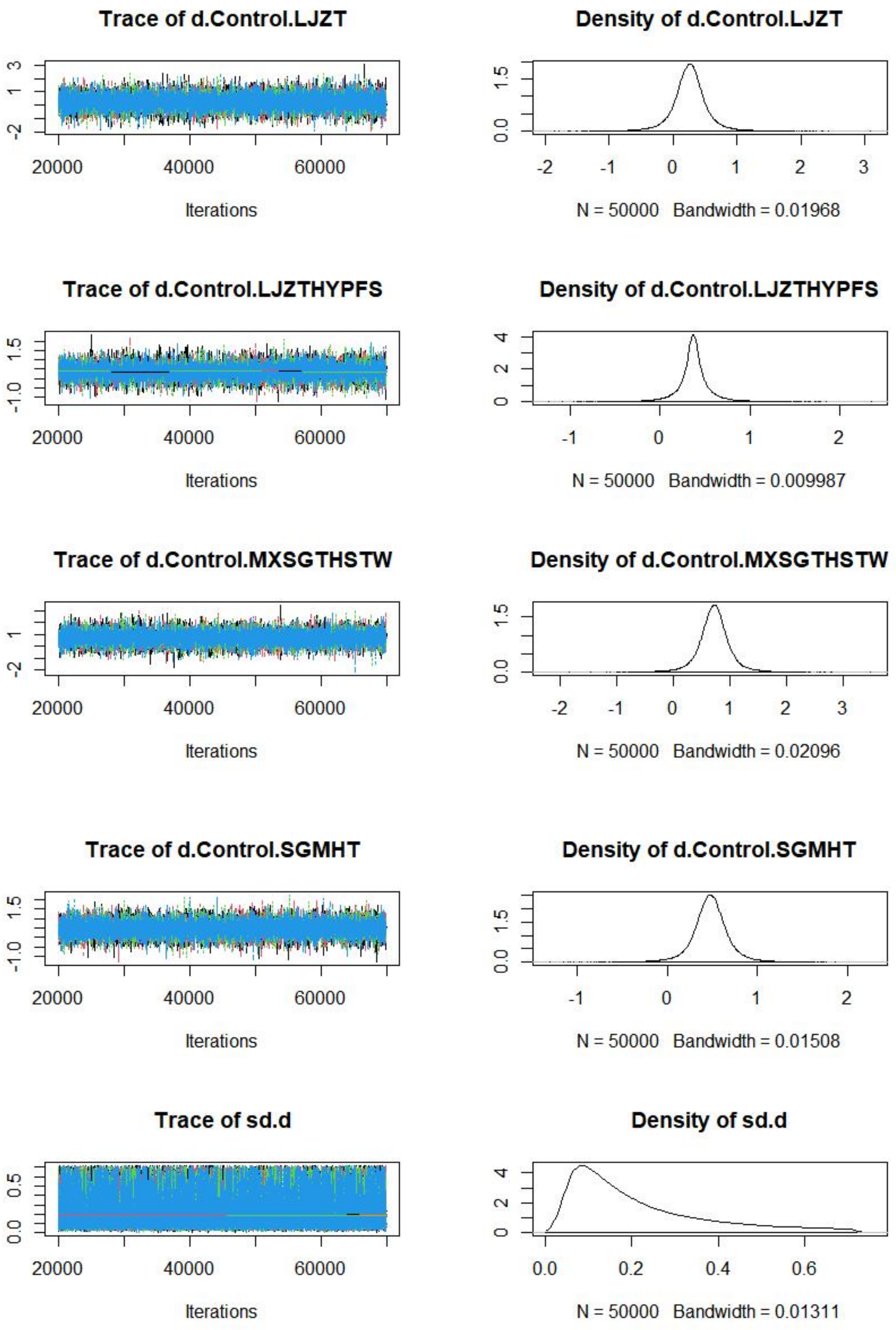

Sensitivity analysis 1

Consistency model - Model fit (residual deviance): fixed

Dbar	pD	DIC
7.162495	6.988442	14.150937

8 data points, ratio 0.8953, I^2 = 2%

Inconsistency model - Model fit (residual deviance): fixed

Dbar	pD	DIC
7.180724	7.006685	14.187410

8 data points, ratio 0.8976, I^2 = 3%

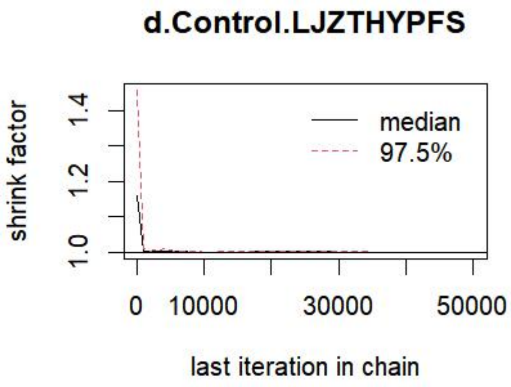

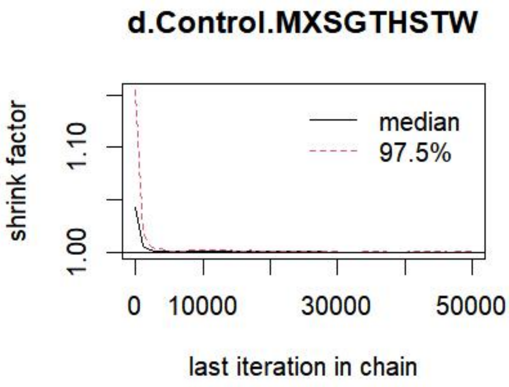

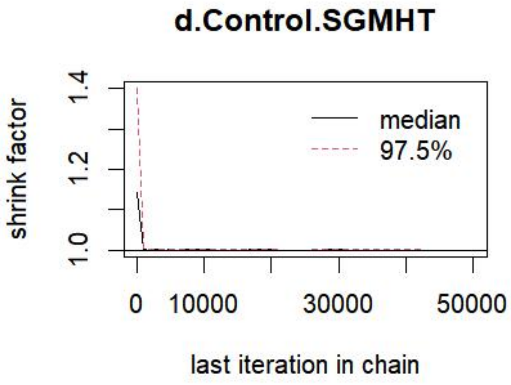

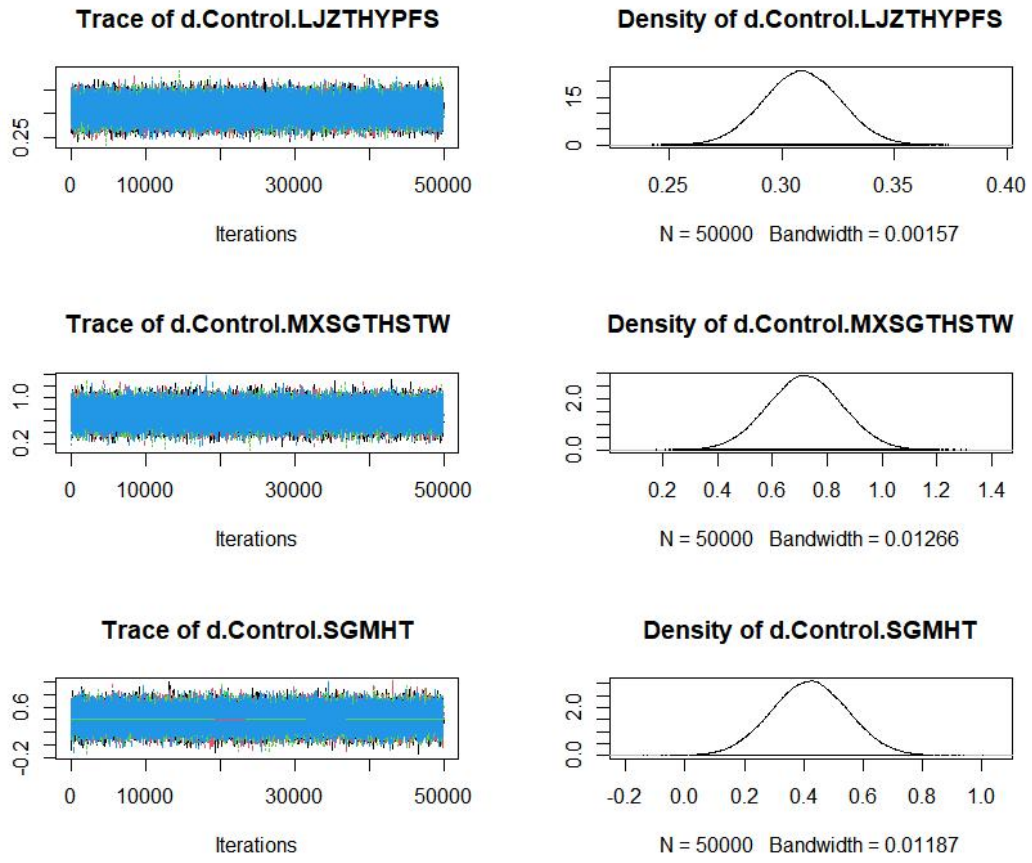

Sensitivity analysis 2:

There were no changes in the RCTs included compared with the main analysis; therefore, this analysis is unnecessary.

2.3 IgE

Consistency model - Model fit (residual deviance): random

Dbar	pD	DIC
10.08892	10.01293	20.10185

10 data points, ratio 1.009, I^2 = 11%

Inconsistency model - Model fit (residual deviance): random

Dbar	pD	DIC
10.08674	10.01276	20.09951

10 data points, ratio 1.009, I^2 = 11%

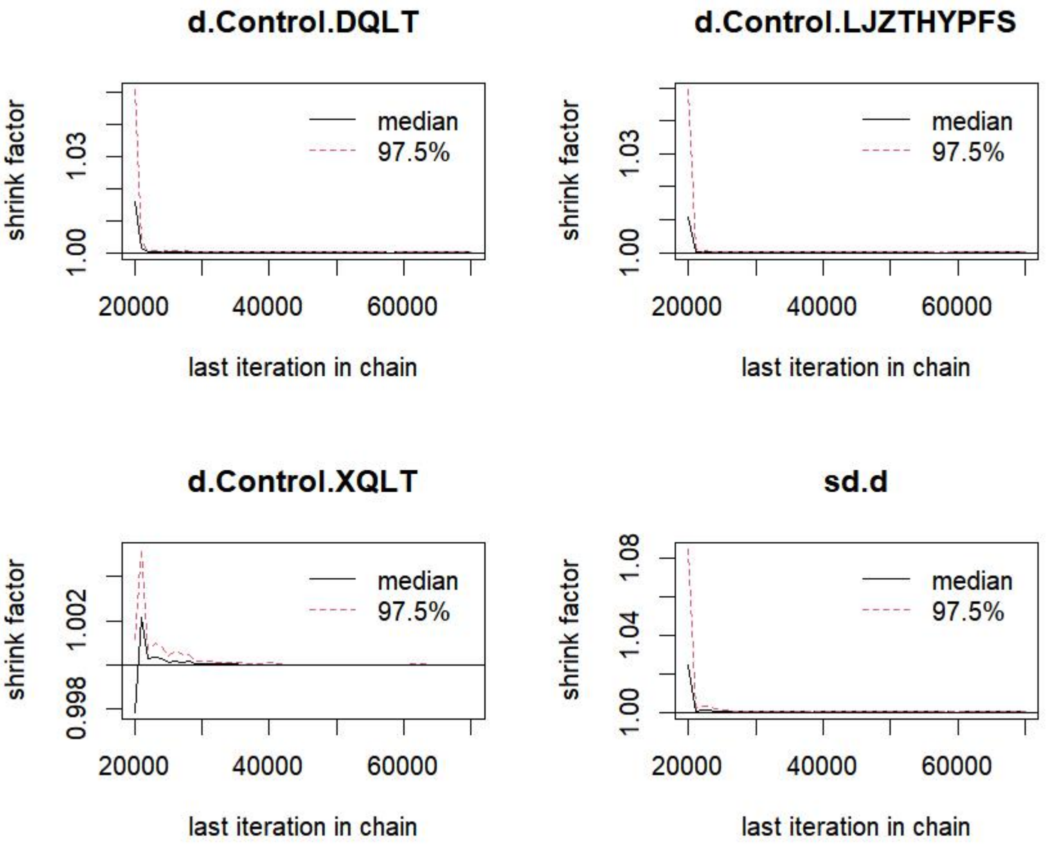
d.Control.DQLT
d.Control.LJZTHYPFS
d.Control.XQLT
sd.d
shrink factor
last iteration in chain
median
97.5%

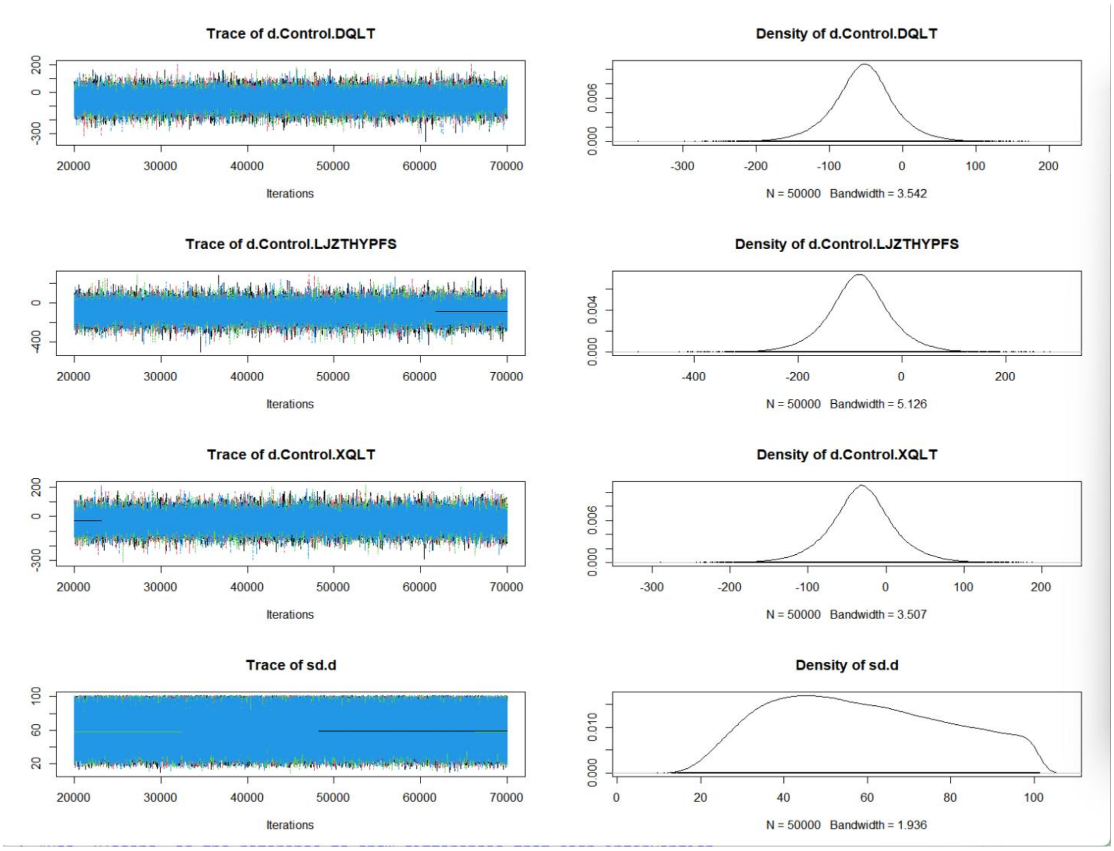
Trace of d.Control.DQLT
Density of d.Control.DQLT
N = 50000 Bandwidth = 3.542
Trace of d.Control.LJZTHYPFS
Density of d.Control.LJZTHYPFS
N = 50000 Bandwidth = 5.126
Trace of d.Control.XQLT
Density of d.Control.XQLT
N = 50000 Bandwidth = 3.507
Trace of sd.d
Density of sd.d
N = 50000 Bandwidth = 1.936
Iterations

Sensitivity analysis 1:

Consistency model - Model fit (residual deviance): fixed

Dbar	pD	DIC
5.992649	5.992583	11.985232

6 data points, ratio 0.9988, I^2 = 17%

Inconsistency model - Model fit (residual deviance): fixed

Dbar	pD	DIC
6.000669	6.000573	12.001241

6 data points, ratio 1, I^2 = 17%

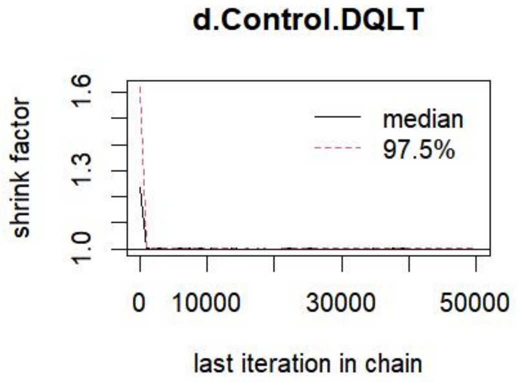

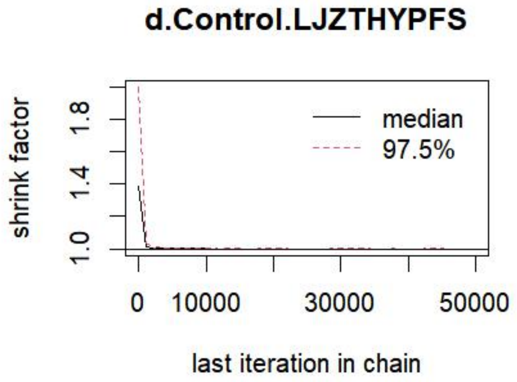

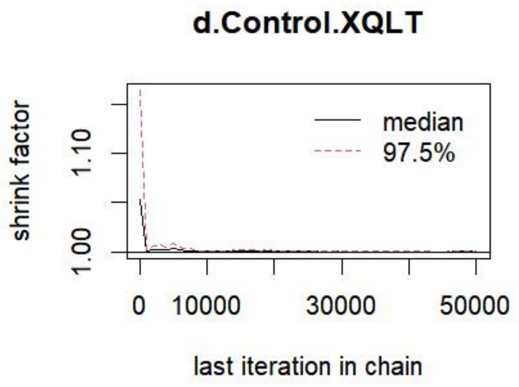

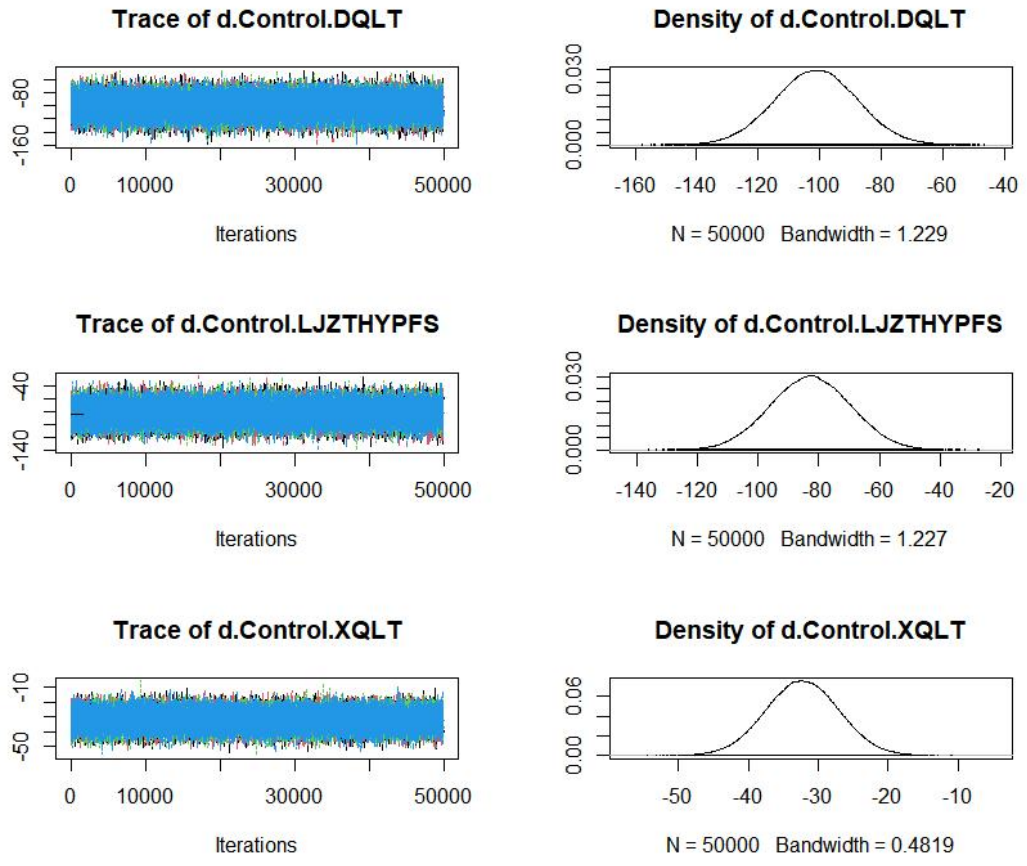

Sensitivity analysis 2:

Consistency model - Model fit (residual deviance): random

Dbar	pD	DIC
14.43726	13.41184	27.84909

14 data points, ratio 1.031, I^2 = 10%

Inconsistency model - Model fit (residual deviance): random

Dbar	pD	DIC
14.47862	13.44267	27.92129

14 data points, ratio 1.034, I^2 = 10%

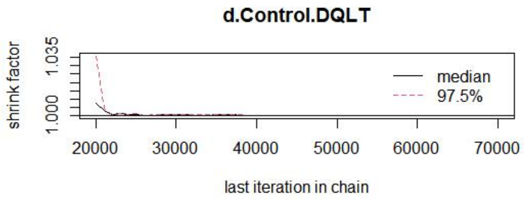
d.Control.DQLT
shrink factor
1.000
1.035
median
97.5%
20000
30000
40000
50000
60000
70000
last iteration in chain

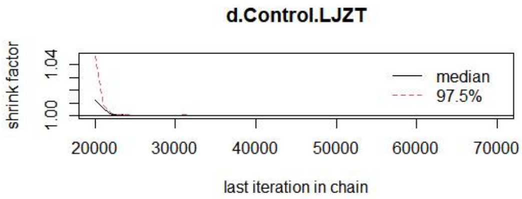
d.Control.LJZT
shrink factor
1.00
1.04
median
97.5%
20000
30000
40000
50000
60000
70000
last iteration in chain

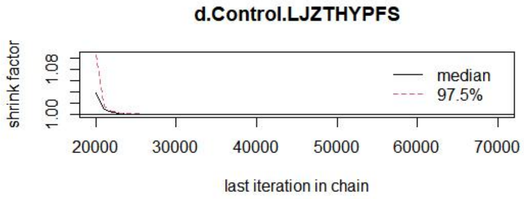
d.Control.LJZTHYPFS
shrink factor
1.00
1.08
median
97.5%
20000
30000
40000
50000
60000
70000
last iteration in chain

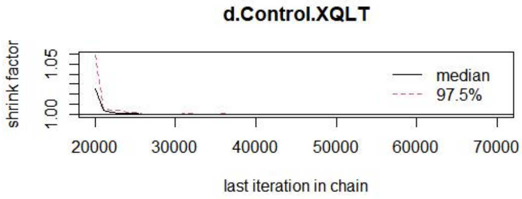
d.Control.XQLT
shrink factor
1.00
1.05
median
97.5%
20000
30000
40000
50000
60000
70000
last iteration in chain

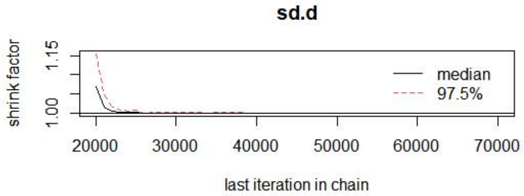
sd.d
shrink factor
1.00
1.15
median
97.5%
20000
30000
40000
50000
60000
70000
last iteration in chain

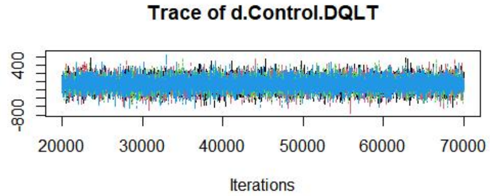
Trace of d.Control.DQLT
-800
400
20000
30000
40000
50000
60000
70000
Iterations

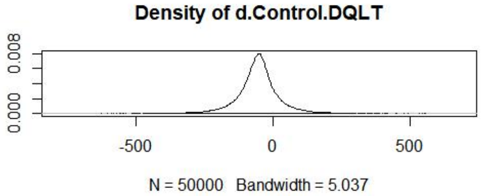
Density of d.Control.DQLT
0.000
0.008
-500
0
500
N = 50000 Bandwidth = 5.037

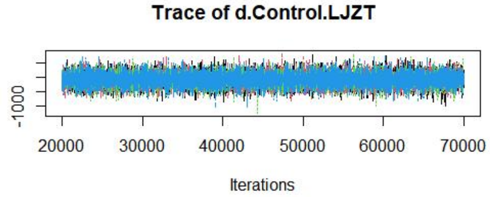
Trace of d.Control.LJZT
-1000
20000
30000
40000
50000
60000
70000
Iterations

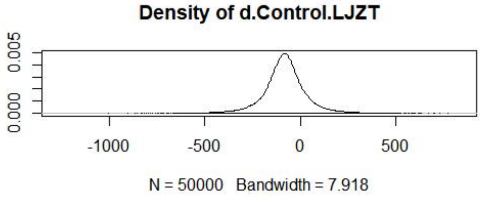
Density of d.Control.LJZT
0.000
0.005
-1000
-500
0
500
N = 50000 Bandwidth = 7.918

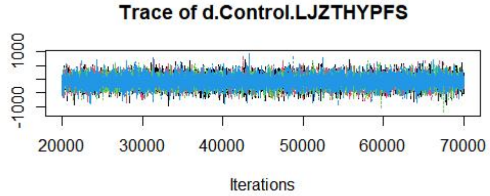
Trace of d.Control.LJZTHYPFS
-1000
0
1000
20000
30000
40000
50000
60000
70000
Iterations

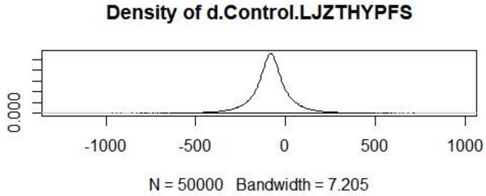
Density of d.Control.LJZTHYPFS
0.000
-1000
-500
0
500
1000
N = 50000 Bandwidth = 7.205

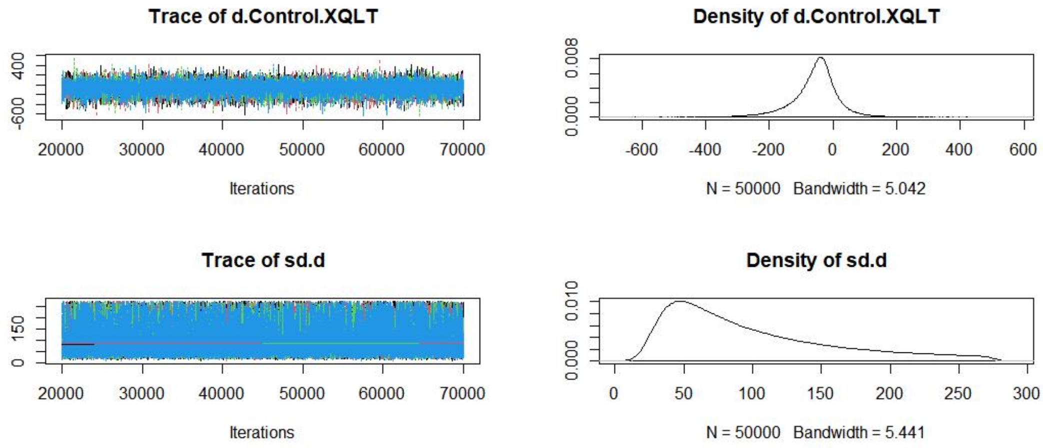

2.4 IgG

Consistency model - Model fit (residual deviance): random

Dbar	pD	DIC
10.51686	10.15331	20.67017

10 data points, ratio 1.052, I^2 = 14%

Inconsistency model - Model fit (residual deviance): random

Dbar	pD	DIC
10.50587	10.14643	20.65230

10 data points, ratio 1.051, I^2 = 14%

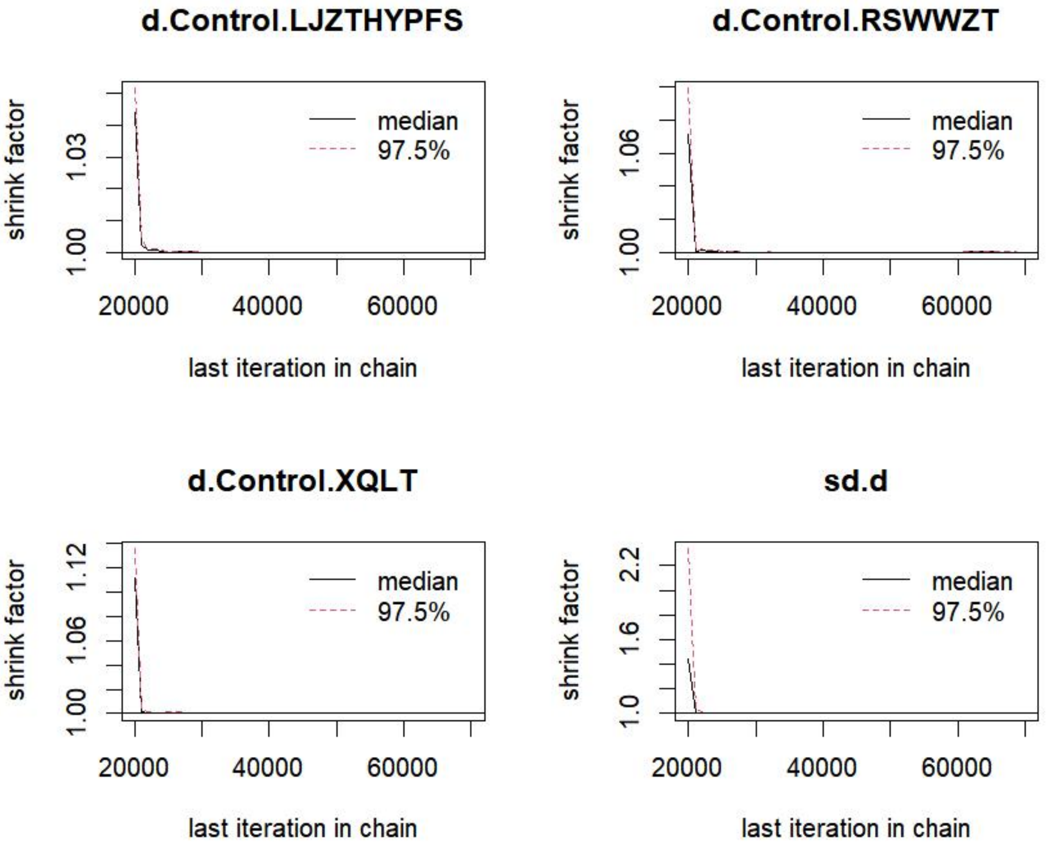

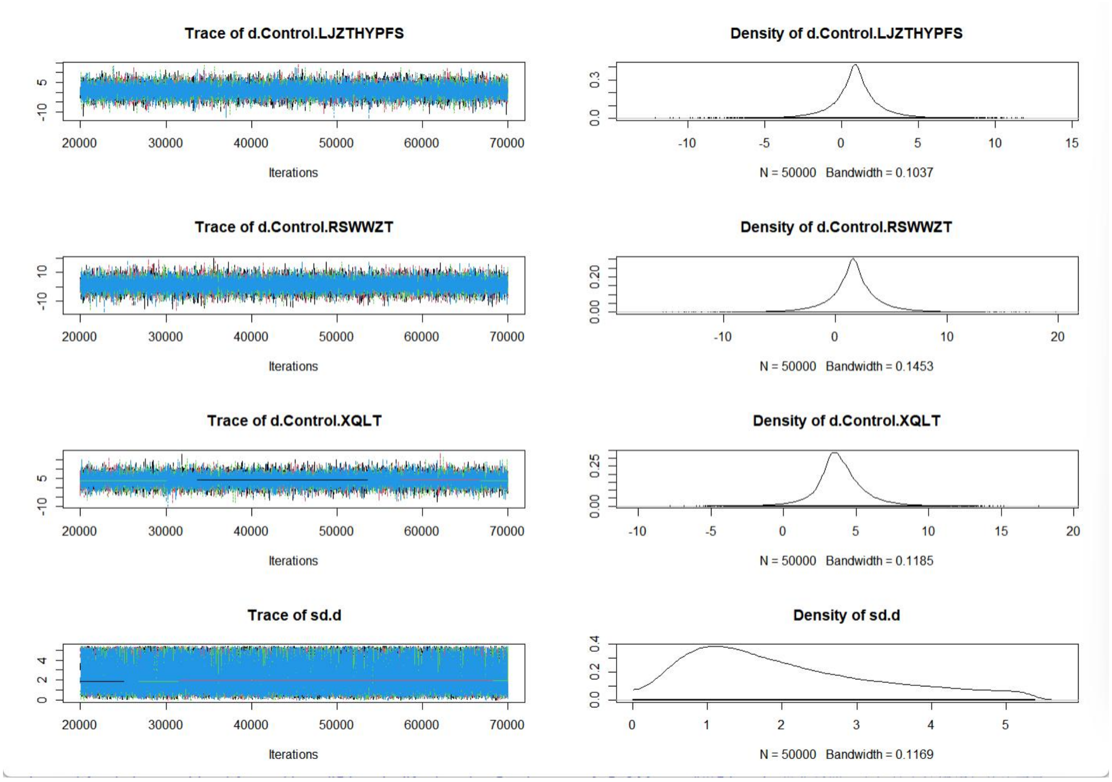

Sensitivity analysis 1

Consistency model - Model fit (residual deviance): fixed

Dbar	pD	DIC
3.998180	3.997993	7.996173

4 data points, ratio 0.9995, I^2 = 25%

Inconsistency model - Model fit (residual deviance): fixed

Dbar	pD	DIC
4.002780	4.002777	8.005557

4 data points, ratio 1.001, I^2 = 25%

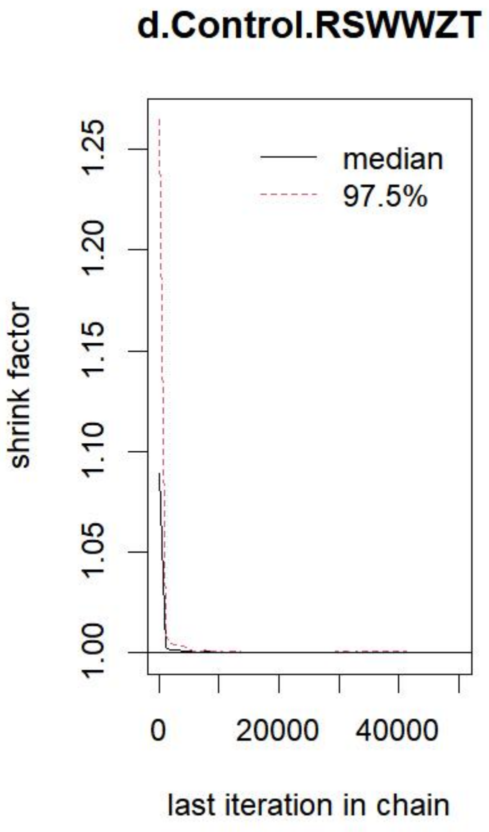

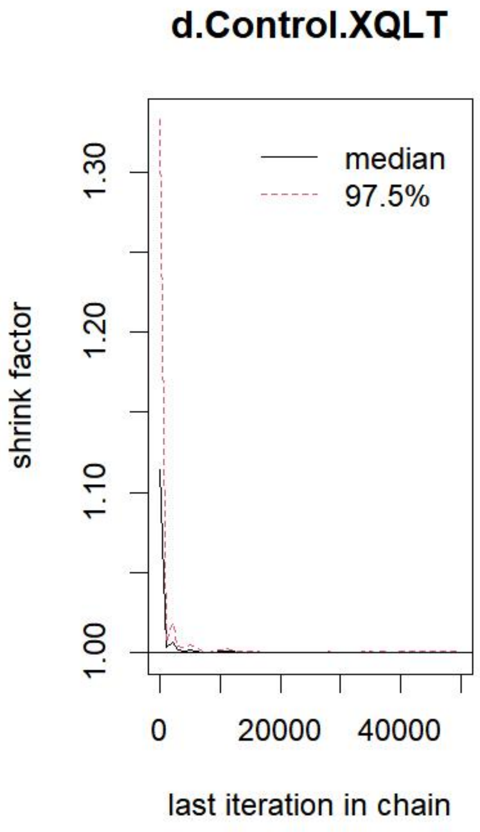

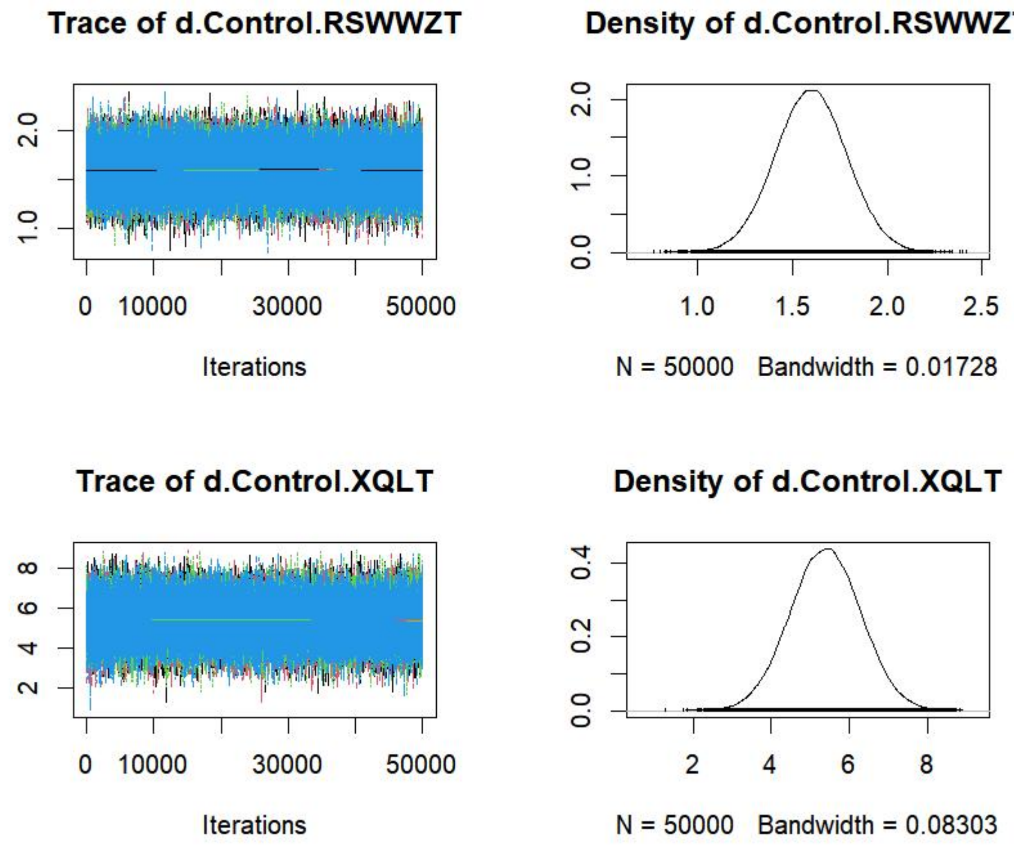

Sensitivity analysis 2:

There were no changes in the RCTs included compared with the main analysis; therefore, this analysis is unnecessary.

2.5 PEF

Consistency model - Model fit (residual deviance): random

Dbar	pD	DIC
20.01961	19.87473	39.89434

20 data points, ratio 1.001, I^2 = 5%

Inconsistency model - Model fit (residual deviance): random

Dbar	pD	DIC
19.96818	19.81797	39.78616

20 data points, ratio 0.9984, I^2 = 5%

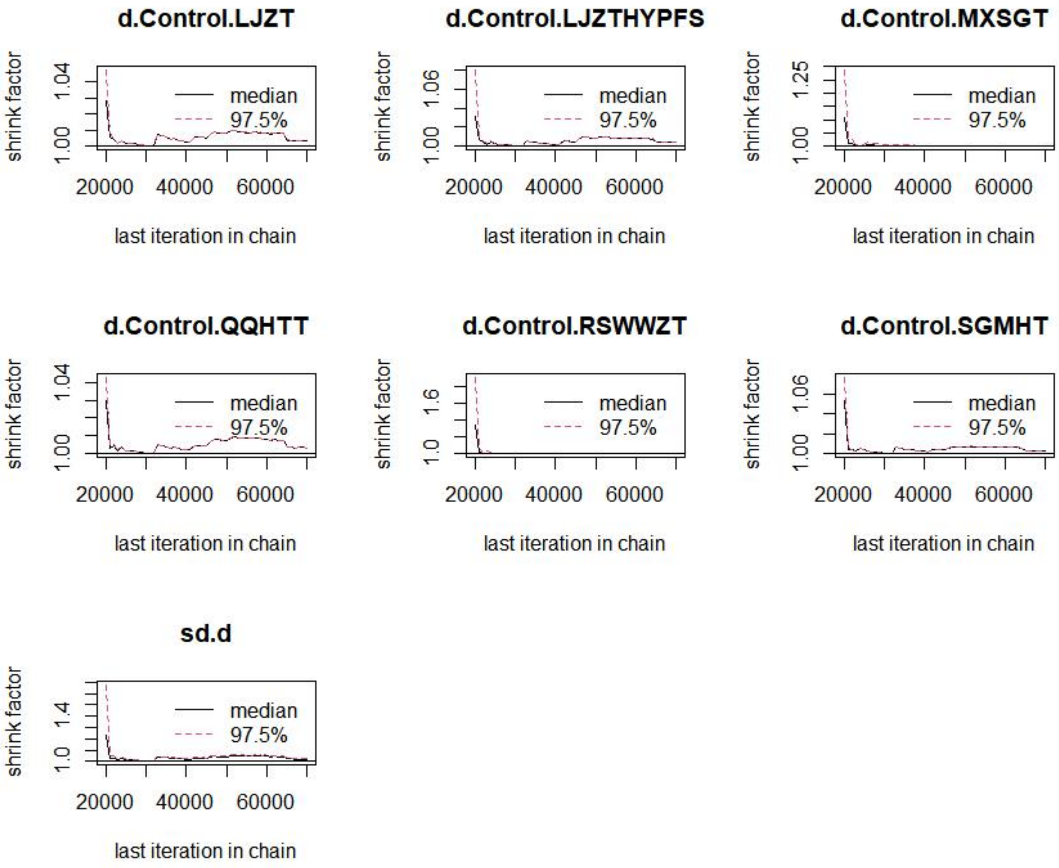
d.Control.LJZT
d.Control.LJZTHYPFS
d.Control.MXSGT
d.Control.QQHTT
d.Control.RSWWZT
d.Control.SGMHT
sd.d
shrink factor
median
97.5%
last iteration in chain
20000 40000 60000

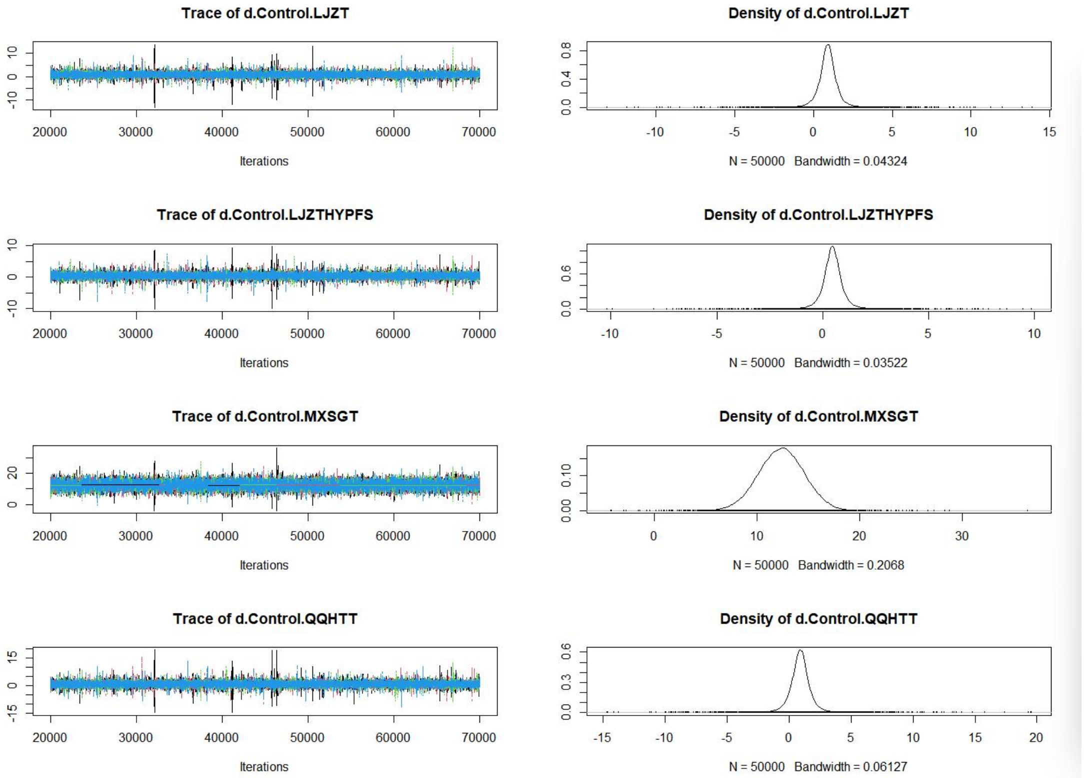
Trace of d.Control.LJZT
Density of d.Control.LJZT
N = 50000 Bandwidth = 0.04324
Trace of d.Control.LJZTHYPFS
Density of d.Control.LJZTHYPFS
N = 50000 Bandwidth = 0.03522
Trace of d.Control.MXSGT
Density of d.Control.MXSGT
N = 50000 Bandwidth = 0.2068
Trace of d.Control.QQHTT
Density of d.Control.QQHTT
N = 50000 Bandwidth = 0.06127
Iterations

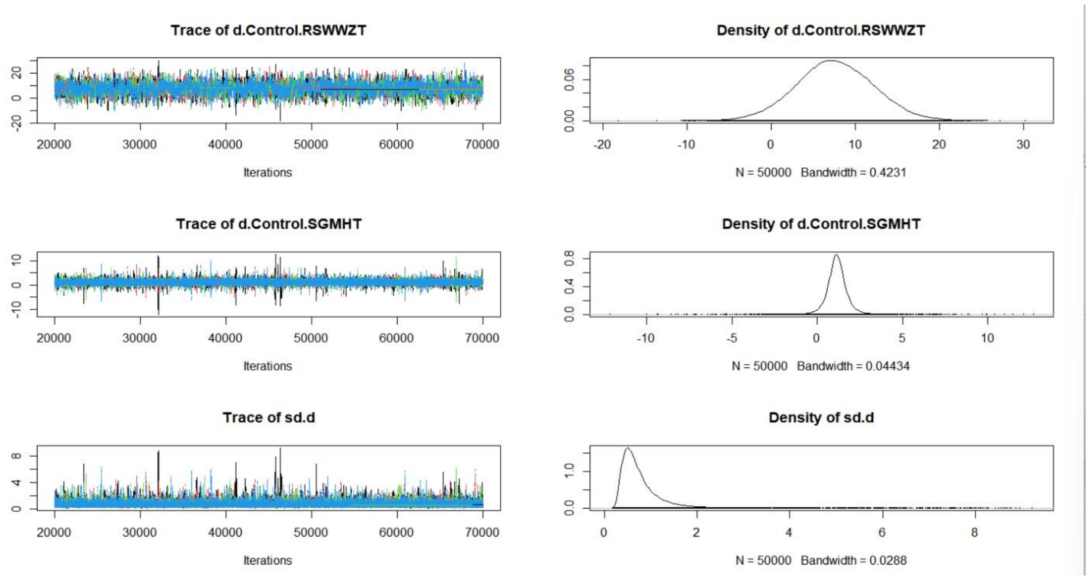

Sensitivity analysis 1:

Consistency model - Model fit (residual deviance): fixed

Dbar	pD	DIC
11.878020	8.001908	19.879928

10 data points, ratio 1.188, I^2 = 24%

Inconsistency model - Model fit (residual deviance): fixed

Dbar	pD	DIC
11.886150	8.009888	19.896038

10 data points, ratio 1.189, I^2 = 24%

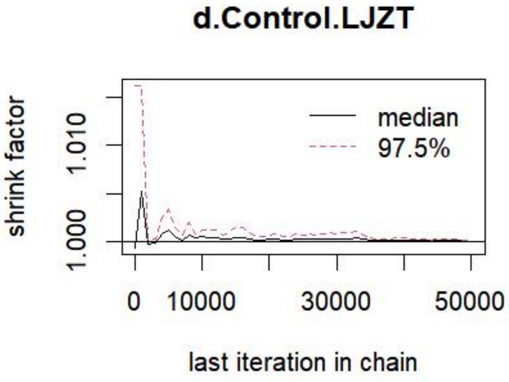
d.Control.LJZT
shrink factor
1.000
1.010
median
97.5%
0
10000
30000
50000
last iteration in chain

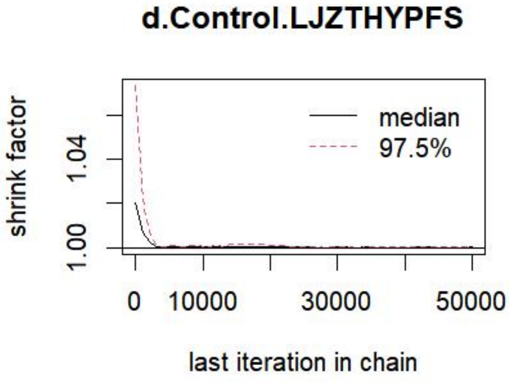
d.Control.LJZTHYPFS
shrink factor
1.00
1.04
median
97.5%
0
10000
30000
50000
last iteration in chain

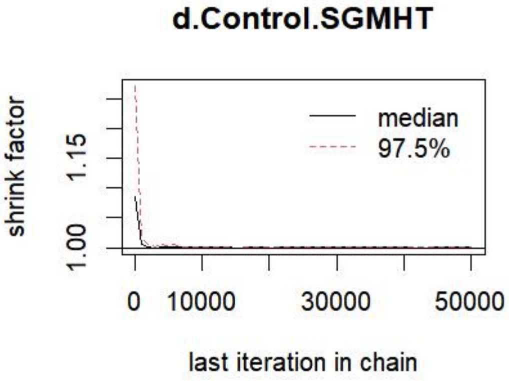
d.Control.SGMHT
shrink factor
1.00
1.15
median
97.5%
0
10000
30000
50000
last iteration in chain

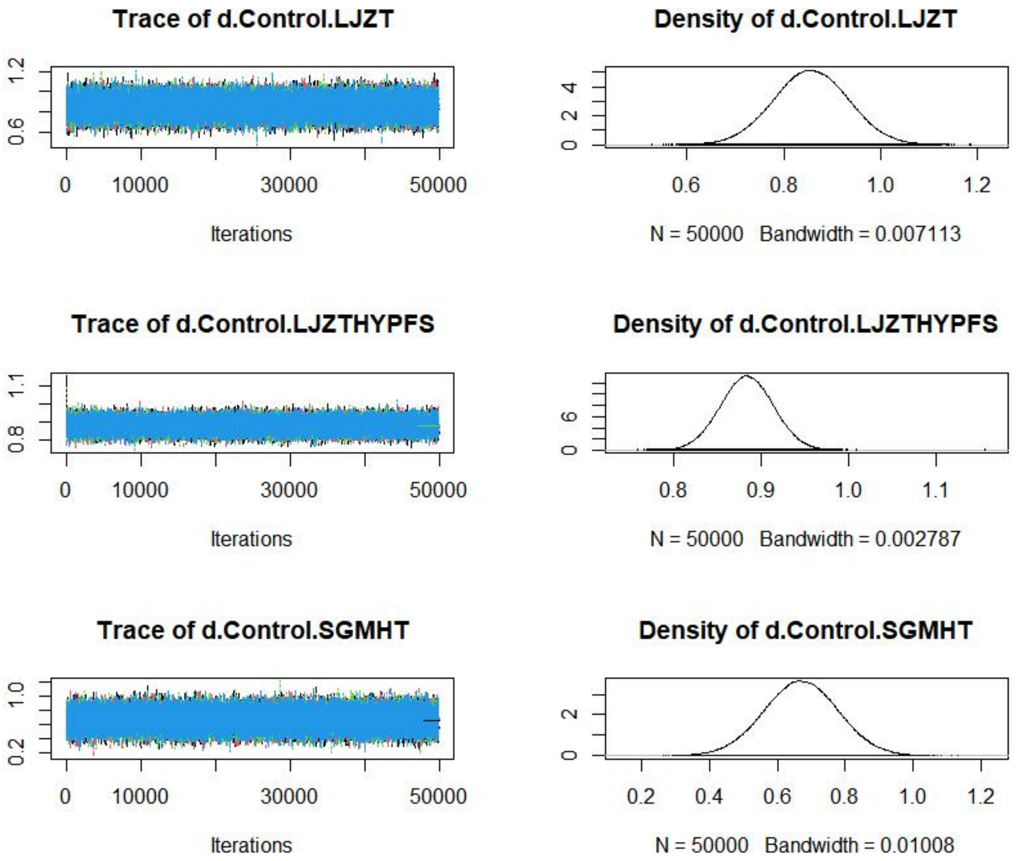

Sensitivity analysis 2

Consistency model - Model fit (residual deviance): random

Dbar	pD	DIC
26.85891	22.11155	48.97046

24 data points, ratio 1.119, I^2 = 14%

Inconsistency model - Model fit (residual deviance): random

Dbar	pD	DIC
26.84907	22.13991	48.98898

24 data points, ratio 1.119, I^2 = 14%

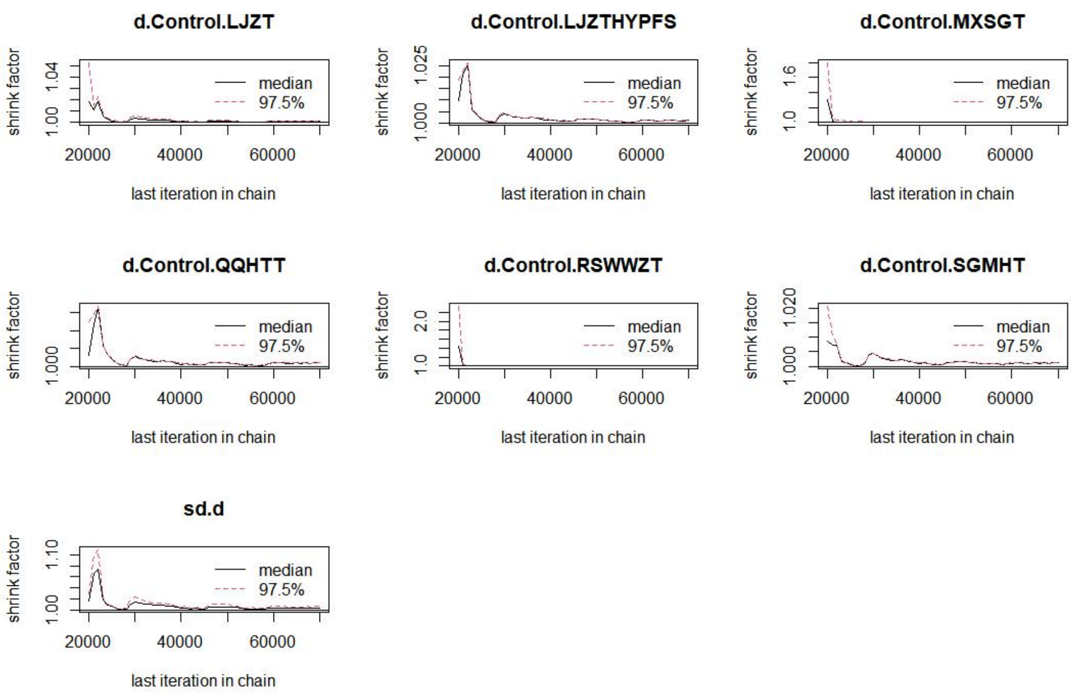
d.Control.LJZT
d.Control.LJZTHYPFS
d.Control.MXSGT
d.Control.QQHTT
d.Control.RSWWZT
d.Control.SGMHT
sd.d
shrink factor
median
97.5%
last iteration in chain

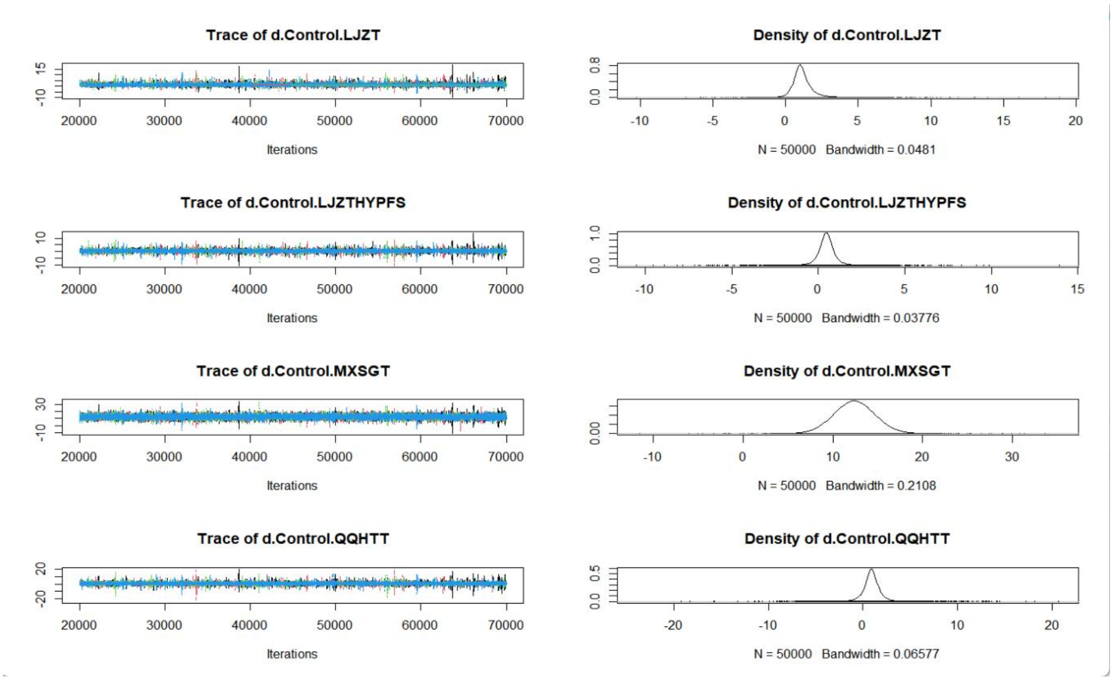
Trace of d.Control.LJZT
Density of d.Control.LJZT
N = 50000 Bandwidth = 0.0481
Trace of d.Control.LJZTHYPFS
Density of d.Control.LJZTHYPFS
N = 50000 Bandwidth = 0.03776
Trace of d.Control.MXSGT
Density of d.Control.MXSGT
N = 50000 Bandwidth = 0.2108
Trace of d.Control.QQHTT
Density of d.Control.QQHTT
N = 50000 Bandwidth = 0.06577
Iterations

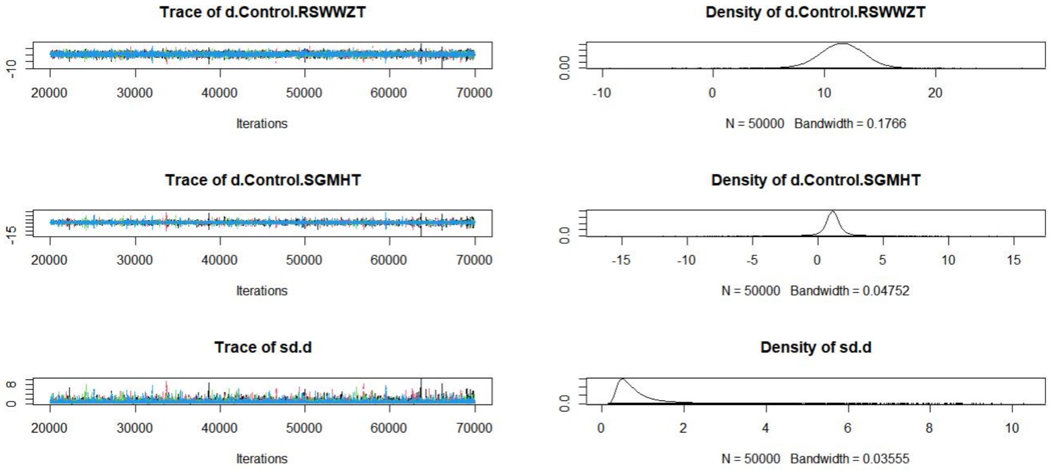
Trace of d.Control.RSWWZT
-10
20000
30000
40000
50000
60000
70000
Iterations
Density of d.Control.RSWWZT
0.00
-10
0
10
20
N = 50000 Bandwidth = 0.1766
Trace of d.Control.SGMHT
-15
20000
30000
40000
50000
60000
70000
Iterations
Density of d.Control.SGMHT
0.0
-15
-10
-5
0
5
10
15
N = 50000 Bandwidth = 0.04752
Trace of sd.d
0
8
20000
30000
40000
50000
60000
70000
Iterations
Density of sd.d
0.0
0
2
4
6
8
10
N = 50000 Bandwidth = 0.03555
